# Splitting the yeast centromere by recombination

**DOI:** 10.1093/nar/gkad1110

**Published:** 2023-11-22

**Authors:** Stanislav G Kozmin, Margaret Dominska, Dao-Qiong Zheng, Thomas D Petes

**Affiliations:** Department of Molecular Genetics and Microbiology, Duke University, Durham, NC, USA; Department of Molecular Genetics and Microbiology, Duke University, Durham, NC, USA; Ocean College, Zhejiang University, Zhoushan, China; Department of Molecular Genetics and Microbiology, Duke University, Durham, NC, USA

## Abstract

Although fusions between the centromeres of different human chromosomes have been observed cytologically in cancer cells, since the centromeres are long arrays of satellite sequences, the details of these fusions have been difficult to investigate. We developed methods of detecting recombination within the centromeres of the yeast *Saccharomyces cerevisiae* (intercentromere recombination). These events occur at similar rates (about 10^−8^/cell division) between two active or two inactive centromeres. We mapped the breakpoints of most of the recombination events to a region of 43 base pairs of uninterrupted homology between the two centromeres. By whole-genome DNA sequencing, we showed that most (>90%) of the events occur by non-reciprocal recombination (gene conversion/break-induced replication). We also found that intercentromere recombination can involve non-homologous chromosome, generating whole-arm translocations. In addition, intercentromere recombination is associated with very frequent chromosome missegregation. These observations support the conclusion that intercentromere recombination generally has negative genetic consequences.

## Graphical Abstract

**Graphical Abstract:**
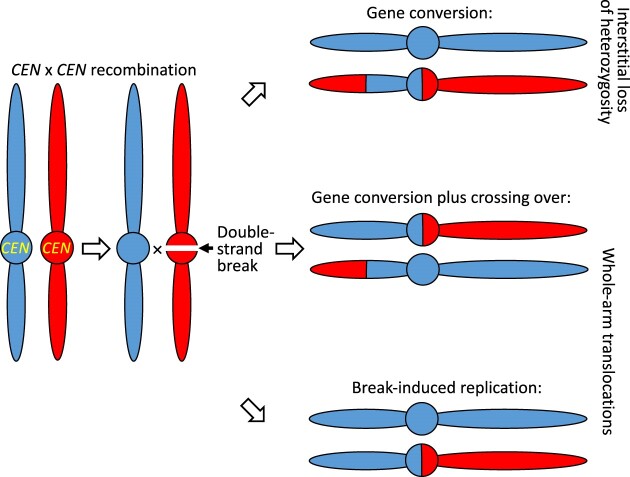

## Introduction

Centromeres are specialized chromosomal regions that serve as sites for kinetochore assembly and microtubule attachment (1). Centromeres are essential for the accurate segregation of chromosomes in mitosis and meiosis. The amount of DNA that makes up the core centromere in eukaryotes varies tremendously from a median length of 118 bp for the ‘point’ centromere of *Saccharomyces cerevisiae* to several Mb for human centromeres.

### Structure of yeast and human centromeres

In *S. cerevisiae*, the sizes of the 16 centromeres in the standard S288c strain vary from 111–120 bp (Saccharomyces Genome Database). Each centromere has three parts: CDEI (8 bp with a consensus of 5′ RTCACRTG), CDEII (76–98% AT-rich region of 78–87 bp) and CDEIII (23 bp) (1–3). Although alterations of CDEI or CDEII substantially elevate chromosome non-disjunction (4–6), only base substitutions in CDEIII completely or nearly completely inactivate centromere function. In particular, any substitution of a second cytosine in the 5′-(T/A)(T/A)C**C**GAA-3′ CDEIII motif that is absolutely conserved in all yeast centromeres (7), causes complete centromere inactivation (2,8,9).

The sequences of the centromeres of the 16 yeast chromosomes are quite diverged. The level of identity among the centromeres of the strain S288c varies between 51% (*CEN6* versus *CEN15*) to 70% (*CEN1* versus *CEN4*) (*Saccharomyces* Genome Database (SGD); yeastgenome.org). The centromeric DNA contains one nucleosome in which the canonical H3 histone is replaced by the H3 variant Cse4, equivalent to CENP-A in humans (1). The assembly of about 50 different proteins at the kinetochore is dependent on the localization of Cse4/CENP-A (1,10). In *S. cerevisiae*, each kinetochore is bound by a single microtubule (11).

In addition to the kinetochore, the chromosome-segregation function of the yeast cells is promoted by the structural properties of the pericentric sequences. In *S. cerevisiae*, the chromatin extending about 25 kb to each side of the centromere has high levels of cohesin that promote the formation of loops (1); these loops produce a ‘bottlebrush’ configuration that helps position the centromeres within the spindle.

In contrast to the tiny *S. cerevisiae* centromeres, the human centromeres consist of very large arrays of simple repetitive DNA sequences. The active centromeric core array has between 340 kb to 4.8 Mb of alpha satellite; the size of this region is homolog-specific (12). The alpha satellite is a 171 bp repeat that is often organized into high-order repeats (HORs). Less than half of the tandemly-repeated alpha satellite repeats within each centromere bind CENP-A (the homolog of Cse4) (12,13). Instead of binding a single microtubule as observed for the point centromere, human centromeres bind an average of 20 microtubules (14). The region of the human centromere consisting primarily of alpha satellite sequences is flanked by large (>1 Mb) pericentric regions consisting of other types of simple repeats (12). Recent sequencing data have revealed the presence of inversions within the satellite sequences of the pericentric region (12,15,16); as discussed below, recombination between such inversions can result in complex chromosome rearrangements. The pericentric regions also have nucleosomes with specific modifications (17). Despite these differences with the point centromeres, the kinetochores of the human centromeres have a similar protein composition as the point centromere of *S. cerevisiae*, and are associated with DNA loops in the pericentric region (1).

### Centromere-associated recombination in yeast and humans

Studies of the effect of the centromere on recombination in *S. cerevisiae* have been done using markers flanking the centromere rather than directly looking at recombination within the centromere. Various degrees of repression of meiotic recombination near centromeres has been observed in various eukaryotic organisms (reviewed in (18)). In *Drosophila melanogaster*, meiotic recombination in the highly-repetitive heterochromatin of the centromere is completely suppressed (19). In contrast, although *S. cerevisiae* centromeres have lower-than-average levels of meiotic recombination, the observed reduction is modest. In experiments in which the centromere was relocated on the chromosomes, Lambie and Roeder (20) found a 3-fold elevation in recombination in the interval from which the centromere was removed, and a four-fold decrease in the interval into which the centromere was inserted. Symington and Petes (21) found that an insertion of *URA3* within one kb of the centromere had a level of meiotic conversion of about 2%, about half the median frequency of conversion for other yeast loci; interestingly, they also noted that about one-third of the conversion events involving *URA3* extended through the centromere, indicating that the centromere did not act as a barrier to the propagation of a conversion tract. Studies of the global distribution of meiotic recombination events in *S. cerevisiae* (for example, (22,23)) confirmed that the level of meiotic recombination near the centromeres was reduced several-fold relative to other chromosomal sequences.

Although mitotic recombination near the yeast centromeres has not been examined in detail, low-resolution maps indicate that mitotic recombination near the centromere is not substantially suppressed (24,25). In addition, 55% of the mitotic gene conversion tracts that involved a *URA3* gene located about one kb from the centromere included a marker within the centromere (26), indicating that in mitosis as in meiosis, the centromere is not a barrier to conversion. The yeast centromere was shown to be a replication-pausing site (27). Since replication pauses in yeast are preferred sites of double-strand break (DSB) formation (25,28), these observations suggest the possibility that yeast centromeres might have elevated rates of mitotic recombination.

Since the human centromeres consist of long arrays of satellites, some aspects of recombination are likely to be different than for yeast centromeres. The lengths of the satellite arrays vary considerably among different individuals, suggesting the intrachromosomal deletions and duplications can occur within the array as a consequence of unequal homologous exchange between sister arrays or events such as single-strand annealing (29). Although allelic meiotic recombination between centromeres is suppressed between human centromeres (18), Jaco *et al.* (30) used fluorescence microscopy to show that mitotic sister-chromatid recombination events between the centromeres of mouse chromosomes are much more frequent (>100-fold) per kb than recombination events involving the non-telomeric chromosome arms.

Crossovers between centromeres of non-homologous chromosomes would be expected to produce whole-arm translocations, whereas intrachromosomal crossovers between inverted satellite sequences would produce isochromosomes. Both types of chromosomal aberrations dramatically increase in a sub-set of cancer cell lines (reviewed in (31)). In oral squamous carcinoma cells, for example, 60% of chromosome aberration breakpoints were located at centromeric regions (32,33).

Whole-arm translocations can also occur in non-cancer cells. The most frequent events (about 1 in 1000 individuals) are Robertsonian translocations (34,35). Such translocations are fusions of the long arms of two acrocentric chromosomes associated with loss of the short arms. Robertsonian translocation breakpoints are located within the pericentromeres, leading to formation of pseudo-dicentric products, in which only one centromere remains active (31). Carriers of Robertsonian translocations are at increased risk of a variety of genetic diseases such as breast cancer, non-Hodgkin lymphoma, and childhood leukemia (34,35). Thus, if whole-arm translocations can occur via homologous recombination between centromeric regions, the study of intercentromeric recombination has obvious clinical importance.

In addition to exchanges between centromeres on homologous chromosomes or non-homologous chromosomes, homologous recombination also occurs within the centromere or between sister chromatids in mammalian cells (30,36). As mentioned above, physical evidence for mitotic exchange between sister chromatid centromeres was demonstrated in mouse embryonic stem cells, and the frequency of sister chromatid exchange (SCE) was strongly elevated at centromeric regions (30).

### Mechanisms of homologous recombination (HR)

Although *S. cerevisiae*, like other eukaryotes, can repair DNA double-strand breaks (DSBs) by mechanisms that involve interaction with a template with shared sequence homology (homologous recombination or HR) or by non-homologous end-joining (NHEJ), our study of centromere-centromere recombination was designed to detect HR. Below, we will briefly outline the HR pathways that occur in yeast. Although there is evidence that mitotic recombination can be stimulated by single-stranded DNA nicks (29), in our discussion, we will emphasize the better characterized pathways resulting from the repair of DSBs.

**Figure 1.**
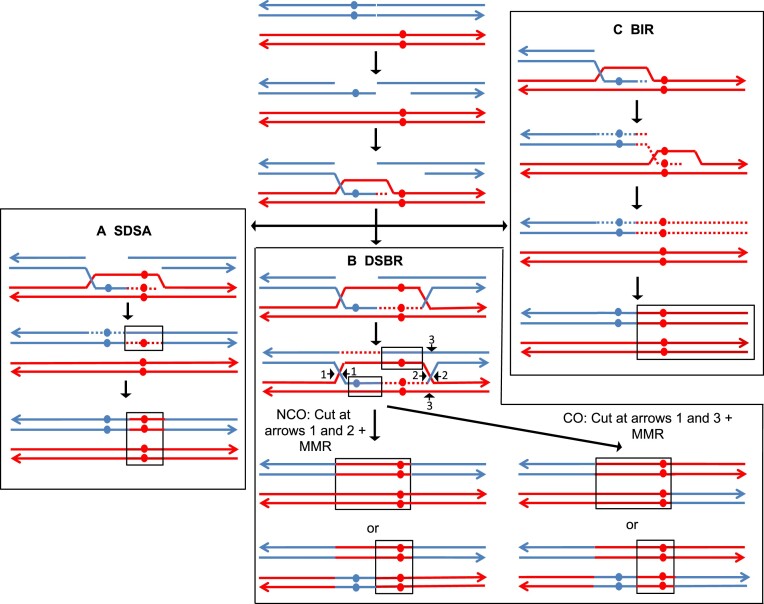
Pathways of homologous recombination. Chromosomes are shown as double-stranded DNA molecules with arrows indicating the 3′ ends with red and blue lines representing the homologs. Polymorphisms between the two homologs are indicated by circles. We show all pathways initiated by a DSB on the blue homolog, followed by 5′ to 3′ processing of the broken ends. One of the 3′ single-stranded ends then invades the unbroken red homolog forming a D-loop. Dotted lines indicate intermediates with newly-synthesized DNA. (**A**) Synthesis-dependent strand annealing (SDSA) pathway. DNA synthesis primed by the invading blue end extends the D-loop. Displacement of the invading strand allows pairing with the second broken end and formation of a mismatch (boxed). Repair of the mismatch using the red strand as a template results in a duplication of the red polymorphism. The net result of these events is a gene conversion event without an associated crossover. (**B**) Double-strand break repair (DSBR) pathway. DNA synthesis from the invading strand results in formation of a double Holliday junction (dHJ) with two mismatched bases. Cleavage of the dHJ as shown in the figure can produce either a non-crossover (NCO) or a crossover (CO) flanking the region of conversion. (**C**) Break-induced replication (BIR) pathway. In this pathway, one broken end is lost, and the other end invades the unbroken homolog, replicating it to the terminus. The net result of this pathway is loss of heterozygosity from the point of invasion to the chromosome end.

For all pathways shown in Figure 1, the ends of the broken chromosome are processed 5′ to 3′ resulting in 3′ single-stranded ‘tails.’ The red and blue circles represent the positions of DNA sequences that are different in the red and blue homologs. In the Synthesis-Dependent-Strand-Annealing (SDSA) (Figure 1A), after invasion of one of the processed ends into the intact homolog followed by DNA synthesis, the invading strand is displaced and reanneals with the second broken end. The heteroduplex region on the upper homolog contains a mismatch that can be repaired to produce a chromosome that has the ‘red’ sequence in both strands. This region of gene conversion is boxed in the figure. The SDSA pathway results in a gene conversion event unassociated with crossing over.

In the classic Double-Strand Break Repair (DSBR) pathway (Figure 1B), the broken end also invades an unbroken homolog, producing a region of heteroduplex. The 3′ end of the invading strand is extended by DNA synthesis resulting in formation of a D-loop and capture of the second broken end. The resulting double Holliday junction (dHJ) is cleaved by structure-specific endonucleases (cleavages indicated by arrows) to yield a non-crossover (cleavages at the arrows marked 1 and 2) or a crossover (cleavages at the arrows marked 1 and 3). In this model, there are heteroduplexes on both sides of the DSB. The mismatches within the heteroduplex can be repaired in the same direction (both to the red allele or both to the blue allele) or in different directions (one to the red allele and one to the blue allele). The pattern of repair will determine whether a gene conversion event will be produced and the extent of the conversion tract. In Figure 1B, we show one pattern of repair that would produce a conversion tract. For crossover events (right side of Figure 1A), the crossover flanks the region of conversion (boxed sequences).

Recombinant chromosomes can also be generated by a non-reciprocal process called Break-Induced Replication (BIR). In this process (Figure 1C), one chromosome fragment resulting from the DSB is lost, and the other end invades the homologous chromosome, setting up a conservative replication event that duplicates sequences to the end of the intact chromosome (37,38). Both DSBR and BIR pathways result in terminal loss of heterozygosity (LOH). In most studies of mitotic recombination, it cannot be determined whether terminal LOH reflects DSBR or BIR because only one of the two daughter cells containing the recombinant products are recovered. In one study of spontaneous mitotic recombination in yeast in which both daughter cells were analyzed, most (>90%) of the terminal LOH events were a consequence of the DSBR pathway (39).

In the present study, we developed a unique experimental system for selecting centromeric recombination events in *S. cerevisiae*. We determined rates of mitotic recombination between centromeres. Our system allowed selection of whole-arm chromosomal translocations resembling those that occur frequently in human cancer cells.

## Materials and methods

### Yeast strains, plasmids, oligonucleotides, and media

The yeast strains used in our study are described in Supplementary Table S1. Details of the strain constructions are in Supplementary Table S1 and in Supp. Materials and Methods. The oligonucleotides used in our analysis are in Supplementary Table S2. As described in Supplementary Table S1, the plasmids pAG25, pAG32, and pFA6-kanMX4 (40,41) were used to insert selectable drug resistance genes during strain constructions.

Yeast strains were grown on rich YEPD or synthetic complete (SC) media (42). To select for transformants with the *hphMX4*, *kanMX4*, and *natMX4* drug resistance genes, we supplemented the medium with 0.3 g/l of hygromycin B, 0.2 g/l of geneticin (G418), or 0.1 g/l of nourseothricin, respectively. SC solid medium without uracil (SC-URA) was used to select Ura^+^ recombinant clones. Medium lacking leucine (SC-LEU) was used to detect the presence of the YJM789-derived *LEU2*-containing chromosome III in diploid hybrids. Medium with 5-fluoroorotic acid (FOA) was used for selection of *ura3* derivatives. 5-FOA medium contained 1 g/l of FOA and 50 mg/l of uracil. Sporulation was induced on plates containing 10 g/l of potassium acetate and 1 g/l of yeast extract.

### Determination of recombination rates

The details of the methods used to calculate recombination rates are given in the Supplemental Materials and Methods. In brief, for each experiment, yeast strains were colony-purified on rich growth medium, and we measured the number of Ura^+^ derivatives and the total number of cells in about 20 colonies. These frequencies were converted to rates using the Lea-Coulson maximum-likelihood method and the 95% confidence limits on the rates were calculated using webSalvador 0.1 software. (https://websalvador.eeeeeric.com/) (43). For each genotype, at least three independent experiments were performed.

### Molecular characterization of the Ura^+^ recombinant clones

**Figure 2.**
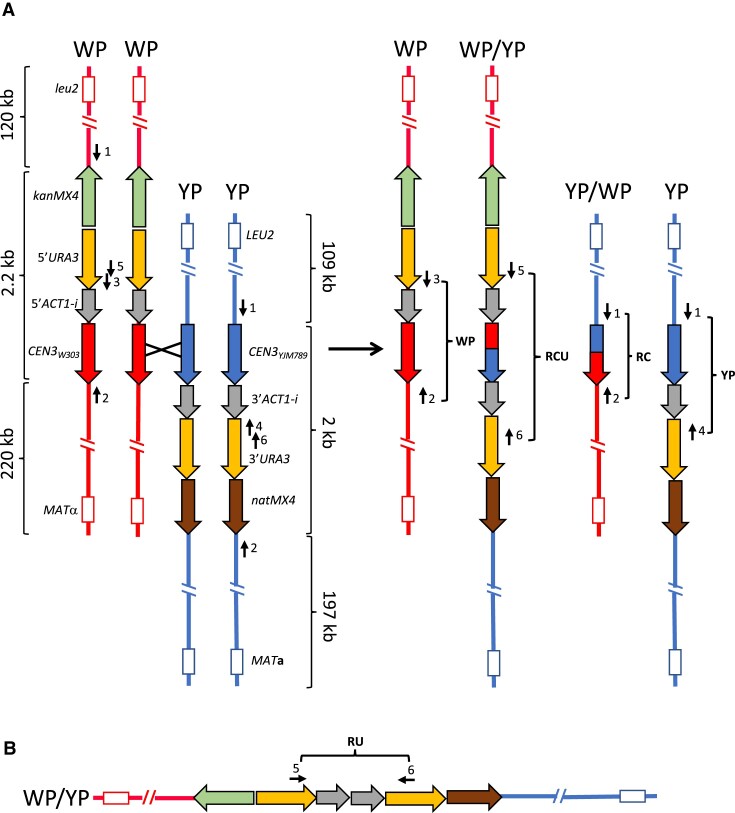
System used to look for *CEN-CEN* recombination with location of primers. (**A**) Structures expected as a consequence of a reciprocal crossover within the centromere. The W303- and YJM789-derived chromosome III homologs are shown in red and blue, respectively. WP and YP indicate chromatids that have the parental centromere configurations for the W303- and YJM789-derived homologs, respectively. WP/YP and YP/WP indicate recombinant centromere configurations. On the W303-derived homolog, *CEN3_w303_* is adjacent to the 5′ end of the *ACT1* intron that is inserted downstream of the 5′ portion of *URA3* whereas the YJM789-derived homolog has *CEN3_YJM789_* adjacent to the 3′ end of the *ACT1* intron that is next to the 3′ end of *URA3*. In addition, each homolog contains a different drug resistance marker. A reciprocal crossover within the centromeric sequences (shown as an X) would result in an intact *ACT1* intron containing a recombinant centromere within a complete *URA3* gene. Assuming that the intron with the centromeric sequences can be spliced from the *URA3* mRNA, this strain should be Ura^+^. The other recombinant product will contain an intact centromere without the *URA3* gene or flanking drug resistance markers. The use of PCR primers to determine the structure of the parental and recombinant centromeres is described in the text, and the locations of these primers are shown in the figure. The code for the primers as named in the text is: 1 (cen3-verF), 2 (cen3-verR), 3 (ura3-intF3), 4 (ura3-intR3), 5(ura3-intF2), and 6 (ura3-intR2). The diagnostic non-recombinant PCR products are labeled WP and YP, and the diagnostic recombinant products are RCU and RC. As discussed in the text, none of the Ura^+^ strains derived from SGK169 had the RC product. (**B**) Structure of a centromere that has a recombinant *URA3* gene lacking a centromere (RU). As discussed in the text, this recombinant gene is likely the result of a strand-switching recombination event involving the *ACT1* gene.

Isolates with recombinant centromeres were characterized by PCR, and the recombinant centromeres were sequenced. In addition, a fraction of the isolates was examined by whole-genome sequencing and/or clamped-homogenous-electric-field (CHEF) gel analysis. For the PCR and sequencing analyses, we used only a single Ura^+^ isolate per culture to ensure their independence. The details of these methods are described in Supplemental Materials and Methods. In brief, genomic DNA from independent isolates was isolated using the method described by Rose *et al.* (42). These samples were examined by PCR using the primers shown in Figure 2. This analysis allowed us to determine whether the chromosomes in the isolate contained centromere sequences of the unrecombined parental chromosome in addition to the recombinant configuration. The PCR products containing the recombinant centromeres were sequenced by the Genewiz (Azenta Life Sciences). Whole-genome sequencing analysis was done as described in Zheng *et al.* (44) and Sui *et al.* (45). Single-nucleotide polymorphism (SNP) microarrays were performed by the methods described in St. Charles *et al.* (46), and CHEF gels were done as outlined by McCulley and Petes (47). Primers used to prepare probes for Southern analysis are in Supplementary Table S2.

### Determination of CEN3-modified chromosome III stability

In our experiments, we made several modifications (including insertion of drug resistance genes) within one kb of the centromere of chromosome III (Figure 2). To determine whether these insertions affected the chromosome loss rate of III, we constructed derivatives of the strains with these markers that also contained a wild-type *URA3* gene 57 kb from *CEN3* on the left arm of III (details in Supplemental Materials and Methods). We measured the rate of 5-FOA-resistant derivatives of these strains (indicating loss of *URA3*) and determined that these derivatives had also lost heterozygous markers on the right arm of chromosome III. The secondary screen is necessary to show that the 5-FOA-resistance reflects chromosome loss rather than mitotic recombination or *ura3* mutations (additional details in Supplemental Materials and Methods).

## Results

### Description of experimental system

The diploids used in our study (SGK169 and isogenic derivatives) were constructed by mating two sequence-diverged haploids, W303-1B and YJM789 (46). The resulting strain was heterozygous for about 55 000 single-nucleotide polymorphisms (SNPs); chromosome III is heterozygous for 807 SNPs as well as being heterozygous for several Ty insertions (48). As will be described below, these SNPs allowed us to map the position of centromere-associated recombination breakpoints at high-resolution by DNA sequencing, and to infer the recombination mechanism. Of particular importance, there are seven SNPs that are heterozygous within *CEN3* in the diploid, allowing us to map the position of exchange within the centromeres.

The system used to select centromere-centromere (*CEN-CEN*) recombinants is shown in Figure 2; it is similar to a system developed previously by Datta *et al.* (49) to select recombinants between diverged DNA sequences. The centromere on the W303 homolog is adjacent to the 5′ portion of the *ACT1* intron that is inserted adjacent to a 5′ portion of *URA3*; the *kanMX4* marker was inserted centromere-distal to the *URA3* promoter on the left arm of III. On the YJM789-derived homolog, the YJM789 *CEN3* is adjacent to the 3′ portion of the *ACT1* intron that is inserted adjacent to the 3′ portion of *URA3*; the *natMX4* marker is located centromere-distal to *URA3* on the right arm of III. The *kanMX* and *natMX* genes share promoter and terminator sequences; these genes are oriented in opposite directions on chromosome III. In addition, all of the *URA3* sequences are located in the split genes without additions or deletions of nucleotides. Because the *URA3* gene is split between the two homologs, the starting diploid is Ura^−^. When a recombination event occurs between the centromeres, a wild-type *URA3* gene with an intron containing the centromere will be generated (Figure 2). Since the intron within the centromere can be spliced from the *URA3* mRNA, the strain should be Ura^+^; we confirmed this expectation by showing that a haploid strain with the *URA3* fusion gene and the intron with the embedded centromere (SGK31, Supplementary Table S1) was Ura^+^.

### Recombination between allelic centromeres on chromosome III in the wild-type diploid yeast strain SGK169

By measuring the frequency of Ura^+^ derivatives in multiple independent cultures and converting these frequencies to rates by fluctuation analysis (details in Materials and Methods), we determined that the rate of *CEN3-CEN3* recombination was about 10^−8^/cell division (1 ± 0.14 × 10^−8^, 95% CL). To confirm that these Ura^+^ isolates were *CEN-CEN* recombinants, we examined about 100 independent Ura^+^ derivatives in detail.

Two different types of analysis were done with all Ura^+^ derivatives. First, we used PCR to map events within and near the centromere sequences. Second, we examined the linkage of two markers located >20 kb from the centromere on the left (*LEU2*) and right (*MAT*) arms of chromosome III; the purpose of this analysis was to determine whether there was a crossover associated with the recombination event at the centromere. Third, we sequenced the recombinant centromeres to identify the recombination breakpoint. Finally, we examined 18 of the 102 Ura^+^ isolates by whole-genome sequencing to analyze complex recombination events.

### PCR analysis of CEN-CEN recombinants

The PCR analysis was performed with four sets of primers from within the centromeric region (Figure 2). The parental homolog with the 5′ end of *URA3* (W303-derived) produced a 274 bp PCR fragment (WP, W303-Parental) with primers cen3-verR and ura3-intF3 (primers 2 and 3 in Figure 2), whereas the parental homolog with the 3′ end of *URA3* (YJM789-derived) produced a 462 bp fragment (YP, YJM789-Parental) with primers cen3-verF and ura3-intR3 (primers 1 and 4 in Figure 2). The Ura^+^ recombinant centromere generated a 489 bp fragment (RCU, Recombinant Centromere and *URA*3) with the primers ura3-intF2 and ura3-intR2 (primers 5 and 6 in Figure 2), and the expected reciprocal product of a crossover resolved in the centromere would generate a product of 270 bp (RC, Recombinant Centromere;) with primers cen3-verF and cen3-verR (primers 1 and 2 in Figure 1). Since the Ura^+^ isolates are derived from diploid strains, we anticipated that most of these isolates would contain products with more than one pair of PCR primers, representing the centromeres located on the two different homologs (Supplementary Figure S1).

All of the Ura^+^ isolates were expected to have the RCU fragment, since the Ura^+^ phenotype requires formation of a fusion between the 5′ and 3′ portions of the *URA3* gene. Of 102 Ura^+^ isolates, 99 had this fragment. With the same primers, four isolates had an unexpected fragment of 372 bp; sequence analysis showed that this fragment contained a recombinant *URA3* gene but lacked a centromere (RU; Recombinant *URA3*).

**Figure 3.**
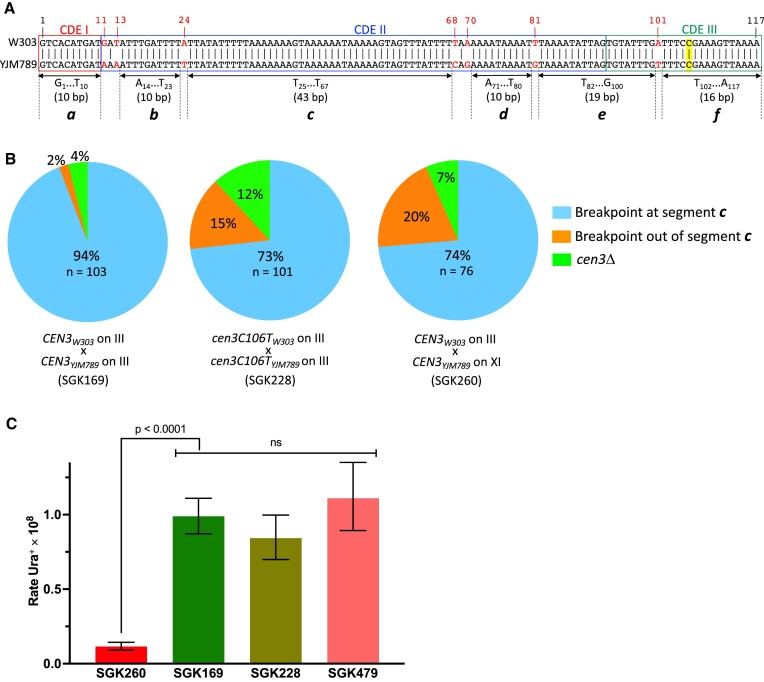
Sequences of parental and recombinant centromeres. (**A**) Sequences of W303 (top line) and YJM789 (bottom line) chromosome III centromeres. The locations of three previously-described centromere domains (CDEI-III, (2)) are shown, as are seven SNPs that distinguish *CEN3* from YJM789 and W303 (red numbers). Mutations of the C nucleotide located at position 106 (highlighted in yellow) inactivate centromere function; a mutation of C to T was used in some of the strains to examine the effect of centromere function on recombination. (**B**) Pie charts showing breakpoints within recombinant centromeres in different strains. The breakpoints for strains SGK169 (*CEN3_W303_* and *CEN3_YJM789_* at normal positions on III), SGK228 (non-functional centromeres *cen3-C106T_W303_* and *cen3-C106T_YJM789_*), and SGK260 (*CEN3_W303_* on chromosome III and *CEN3_YJM789_* on chromosome XI). For all strains, most of the recombinant centromeres had a breakpoint within the 43 bp AT-rich tract near the middle of the centromere. (**C**) Relative rates of *CEN-CEN* recombination (assayed by the rate of Ura^+^ derivatives) in strains SGK169, SGK228 and SGK260 (relevant genotypes in Figure 3B). Strain SGK497 is a derivative of SGK169 that lacks the *MATalpha* locus.

All of the PCR fragments representing recombinant centromeres were sequenced. The SNPs that distinguish the W303-1B and YJM789 chromosome III centromeres are at positions 11, 13, 24, 68, 70, 81 and 101 (Figure 3A). In 97 of the 99 isolates with the RCU fragment, the recombination breakpoint was between the SNPs at positions 24 and 68 (Figure 3B). The 43 bp between these SNPs region is the longest region of uninterrupted homology between the two centromeres. The four Ura^+^ samples with the RU fragment of 372 bp had identical sequences in which the left and right portions of the *ACT1* intron were fused, and there was a perfect deletion of the centromeric sequences. As we will discuss further below, this structure is likely a consequence of ectopic recombination between the *ACT1* intron sequences flanking the centromeres of chromosome III and the wild-type *ACT1* gene located on chromosome VI.

The results from the PCR analysis of the SGK169 isolates are shown in Supplementary Table S3. Although almost all of the Ura^+^ strains contained the RCU PCR fragment diagnostic of one *CEN-CEN* recombinant product (the fusion between the 5′ and 3′ portions of the *URA3* gene), none had the 270 bp RC PCR product expected for the reciprocal crossover (Figure 2). If all of the recombinants were produced by the pathway shown in Figure 2, assuming both recombinant products segregate into the same daughter cell 50% of the time (50), half of Ura^+^ isolates would contain the RC product. Since the reciprocal product was not recovered in any of the SGK169 isolates, we conclude that these Ura^+^ recombinants are not formed by reciprocal exchange within the centromere. However, as will be discussed below, we found five isolates in which the recombinant centromeres were associated with a reciprocal crossover of flanking markers.

Two of the 102 Ura^+^ isolates had genetic alterations that prevented their complete analysis. Based on the PCR analysis, the remaining 100 Ura^+^ isolates were divided into five groups (Table 1): Class 1 (WP + RCU), Class 2 (YP + RCU), Class 3 (WP + RU or YP + RU), Class 4 (RCU, monosome), and Class 5 (WP + YP + RCU, trisome). Classes 1–3 isolates (73 of 100) have two copies of chromosome III, one with the recombinant centromere and one with a parental centromere.

**Table 1.** Classes of CEN-CEN recombinants in SGK169

Class	PCR products	Chromosome configurations	CO/NCO	# of Isolates
1A	WP, RCU	A + L	NCO	23
1B	WP, RCU	A + M	CO	2
1C	WP, RCU	A + K	NCO	1
1D	WP, RCU	C + L	CO	2
1E	WP, RCU	E + M	CO	1
1F	WP, RCU	C + M	CO	1
1G	WP, RCU	E + N	CO	1
**Total Class 1**				31
2A	YP, RCU	B + K	NCO	14
2B	YP, RCU	B + M	CO	5
2C	YP, RCU	D + M	CO	3
2D	YP, RCU	B + L	NCO	8
2E	YP, RCU	B + N	CO	5
2F	YP, RCU	D + K	CO	1
2G	YP, RCU	D + L	CO	2
2H	YP, RCU	D + N	CO	1
**Total Class 2**				39
3A	WP, RU	A + O	CO	1
3B	YP, RU	D + O	CO	1
3C	YP, RU	B + P	NCO	1
**Total Class 3**				3
4A	RCU	L	NCO	3
4B	RCU	M	CO	5
4C	RCU	K	NCO	1
**Total Class 4**				9
5	WP, YP, RCU or WP, YP, RU	Not determined	Not determined	18
**Total Isolates**				100

**Figure 4.**
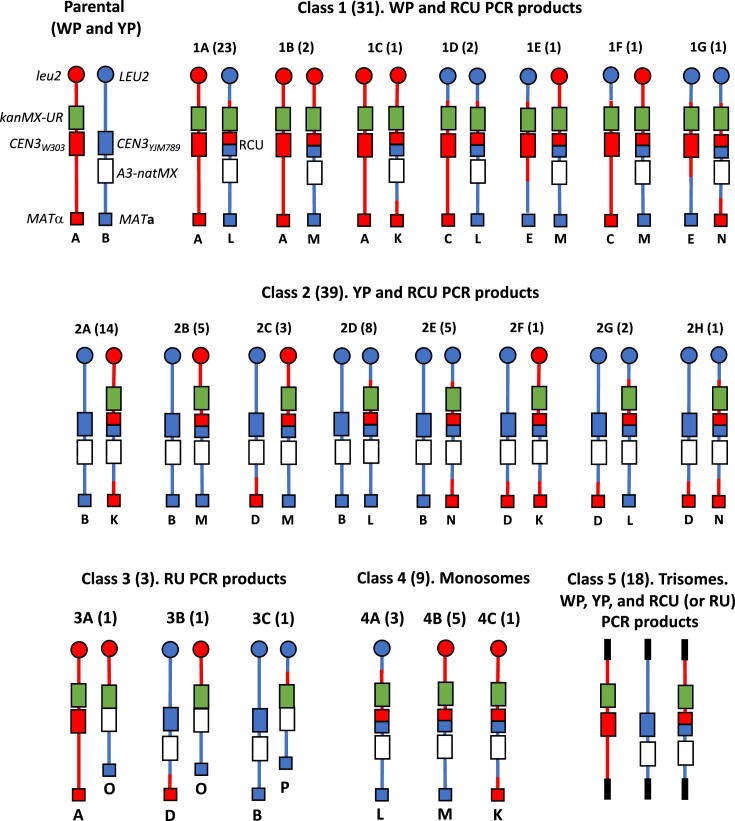
Schematic diagrams of recombinant chromosomes in SGK169 for all classes and sub-classes. The top left part of the figure shows the arrangement of markers in the starting SGK169 strain. Based on our PCR analysis of Ura^+^ isolates, isolates were grouped into five classes. The abbreviations used to describe the PCR fragments were: WP (parental W303 pattern), YP (parental YJM789 pattern), RCU (recombinant centromere and *URA3* gene) and RU (recombinant *URA3* gene without a centromere). Although most of the isolates (Classes 1–3) had two PCR fragments, indicating the presence of two chromosomes, Classes 4 and 5 were monosomic and trisomic for chromosome III, respectively. Based both on PCR analysis and on the linkages of the centromere-flanking markers *LEU2* and *MAT*, individual chromosomes were classified into 11 categories that were given letters in the figure A–E and K–P (Supplementary Figure S2). Twenty-two sub-classes were assigned (1A–1G, 2A–2H, 3A–3C, 4A–4C and 5). The numbers in parentheses after each sub-class indicate the number of isolates observed.

The various classes shown in the first column are depicted in Figure 4. The abbreviations in the second column represent the following centromere configurations (as defined by PCR) on chromosome III: WP (parental W303 centromere), YP (parental YJM789 centromere), RCU (recombinant centromere and recombinant *URA3* gene), RU (recombinant *URA3* gene lacking a centromere). The letters in the third column (A-E and K-P) represent various classes of single chromosomes. These classes (defined in Figure 4 and Supplementary Figure S2) are based on the structure of the centromere and the linkages of flanking markers located on the left and right arms of chromosome III. Based on the arrangement of markers that flank the centromere, in the fourth column, we interpret each class as representing a crossover (CO) or non-crossover (NCO) associated with centromere recombination. In strains with three chromosomes (Class 5), this classification was not done because of ambiguities of interpretation.

### Analysis of the coupling of markers flanking the centromeres

For Classes 1–4, we also determined the coupling of the *LEU2* and *MAT* markers on the homologs. The purpose of this analysis was to determine whether centromere recombination was associated with reciprocal crossing over. In the SGK169 strain, before selection of the Ura^+^ recombinant, the *leu2* gene is linked to *MATalpha* on the W303-derived chromosome and the *LEU2* allele is linked to *MAT***a** (Figure 2). For Classes 1–3 isolates, we isolated monocentric derivatives of each isolate by selecting 5-FOA-resistant derivatives. This procedure selects against the chromosome with the recombinant centromere. Thus, by determining whether these derivatives are Leu^+^ or Leu^−^ using omission media, and *MAT***a** or *MATalpha* using PCR analysis (details in Supp. Materials and Methods), we can determine the marker coupling in the 5-FOA-resistant derivative. By analyzing the same markers in the diploid isolates, we can infer the coupling of markers in the RCU or RU homolog containing the recombinant product. For the monosomic Class 4, we could determine the linkages of the flanking markers directly since there was only a single chromosome. For the Class 5 trisomic strains, because most of the 5-FOA-resistant isolates retained two chromosomes, we did not attempt to infer the linkages of the flanking markers.

These data are summarized in Table 1 and Supplementary table S3. All of the Class 1–3 strains had one chromosome with the parental centromere (WP or YP) and one chromosome with a recombinant centromere (RCU) or a recombinant *URA3* gene lacking a centromere (RU). Based on the analysis of the flanking markers, we divided Classes 1–4 into multiple sub-classes: Classes 1A-1G, 2A-2H, 3A-3B and 4A-4C. The arrangements of markers on individual homologs (labeled A-E and K-P) are shown in Supplementary Figure S2, and the pairs of homologs that define each sub-class are shown schematically in Figure 4.

**Figure 5.**
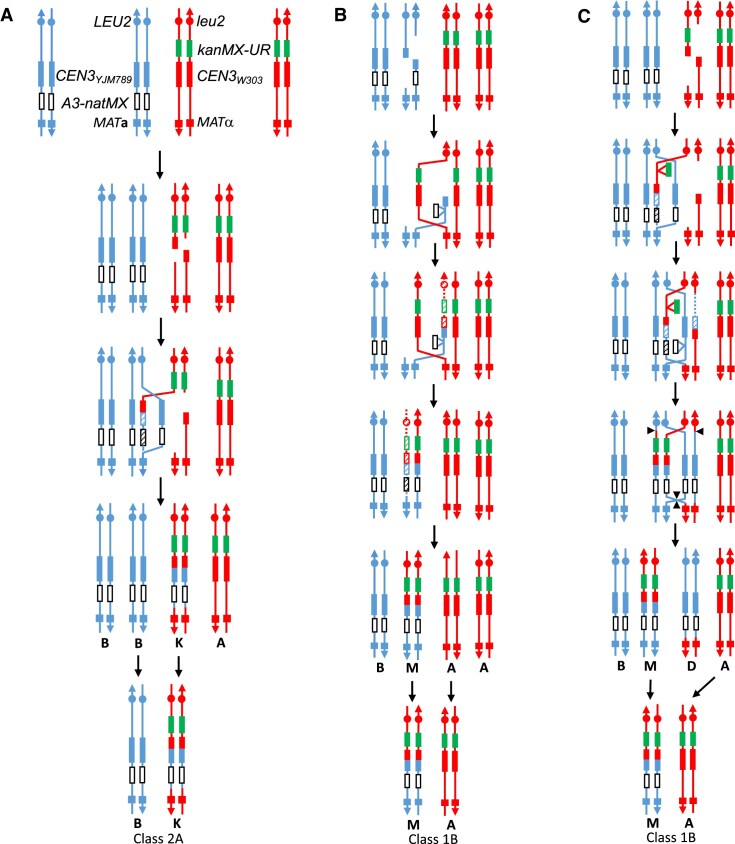
Generation of recombinant isolates by SDSA, BIR and DSBR pathways. We show the duplicated copies of chromosome III, with the red and blue lines representing the W303- and YJM789-derived homologs, respectively. The *ACT1* intronic sequences immediately adjacent to the centromeric sequences (Figure 2) are not shown in this diagram. The recombination events in both panels A and C are initiated by a DSB on one of the W303-derived homologs, whereas the BIR event (panel B) is initiated by a DSB on the YJM789-derived homolog. Newly-synthesized DNA sequences are shown as dotted lines and dashed rectangles. Classes 2A and 1B are shown schematically in Figure 4. (**A**) Class 2A. In this class, the Ura^+^ isolate has one non-recombinant YJM789 chromosome, and one W303-derived chromosome with a recombinant centromere in which the flanking *LEU2* and *MAT* markers are in the parental configuration. This pattern is most simply explained as resulting from an SDSA event in which the chromatids labeled B and K disjoin into the same cell. (**B**) Class 1B. In this class, there is one non-recombinant W303-derived chromosome, and one chromosome with a recombinant centromere and crossing-over of the flanking markers. This pattern can be explained by a BIR event in which the initiating event is a DSB on the YJM789-derived homolog, followed by loss of one of the resulting chromosome fragments. The DSB is repaired by copying the W303-derived homolog by conservative DNA replication. (**C**) Class 1B. The final recombinant products are the same as observed in Figure 5B, but are generated by formation of a double Holliday junction, an associated gene conversion, and cleavage of the junction (shown as triangles) to produce a crossover. In this event, the crossover product marked M co-segregates with the unrecombined W303 chromosome (A).

Several generalizations can be made about the information in Table 1 and Figure 4. First, the most common recombination events for isolates that were not aneuploid were those that contained one unrearranged homolog and one that contained a recombinant centromere with the structure expected for a gene conversion event unassociated with a crossover (Classes 1A and 2A; 37 of 73 events). It is likely that these events are generated by the SDSA pathway (Figures 1 and 5A). Although conversions unassociated with crossovers can also be produced by processing of a dHJ, this pattern of dHJ processing is infrequent (51). Second, isolates in which one or both homologs had a crossover of flanking markers (Classes 1B, 1D, 1E–G, 2B, 2C, 2E–2H, 3A, 3B) were also common (26 of 73 events). These events (crossovers associated with conversions) are consistent with the canonical DSBR model (Figures 1 and 5B) or could be explained by BIR. Third, aneuploidy of the isolates was very common (27 of 100 isolates); 9 isolates were monosomic (Class 4) and 18 were trisomic (Class 5). Of the monosomic isolates, 5 of 9 were associated with a crossover. Lastly, although most of the Ura^+^ isolates (97 of 100) had a recombinant centromere, three Class 3 isolates and one Class 5 isolate had a recombinant *URA3* gene that lacked a centromere; a model to explain this event will be described below.

Mechanistic pathways to generate all of the Class 1 and Class 2 isolates are shown in Figure 5 and Supplementary Figures S3-S15. Although most of the recombinant classes can be explained by the models shown in Figure 1, two types of exceptions were observed. In the standard model of mitotic recombination, sister chromatids disjoin into different daughter cells. Some of the events (Classes 1C, 2B and 2D) are most easily explained by two sister chromatids segregating into the same cell (for example, Supplementary Figure S4). In addition, although many of the events can be explained by the repair of a single DSB, several classes (1D, 1E, 2F, 2G and 2H) appear to reflect the repair of two chromatids broken at the same position (for example, Supplementary Figure S5). Such events are likely initiated by a DSB in one of the homologs, followed by replication of the broken homolog to yield two broken chromatids. Such events have been commonly observed in other studies of mitotic recombination in yeast (52).

Lastly, we point out that although Class 1 and 2 isolates with one crossover were common, isolates in which the homolog pair has reciprocal events were rare (five of 70 strains, Classes 1F, 2C, 3B). This result indicates that either *CEN-CEN* recombinant chromatids tend to segregate into different daughter cells or that many of the terminal LOH events reflect BIR rather than reciprocal crossovers (53). As explained in the Discussion, for most of the recombination events, our experiments cannot distinguish between the DSBR and BIR mechanisms.

### Mechanism leading to formation of acentric recombinant chromosomes in Ura + recombinants of SGK169 (Class 3)

As described above, three of the Ura^+^ isolates contained a *URA3* gene that retained the *ACT1* intron but deleted the *CEN3* sequences. Since the chromosome with the deleted centromere contains replication origins and since we select for strains that are phenotypically Ura^+^, these isolates would retain the recombinant chromosome despite the lack of the centromere. The likely mechanism for generating the acentric chromosome is shown in Supplementary Figure S16. We suggest that the chromosome end resulting from a DSB in the centromere is processed to yield an end within intronic sequences, allowing a strand invasion into the intron of the *ACT1* gene located on chromosome VI. After limited DNA synthesis, the invading end is displaced and recombines with the intron in the chromosome III homolog. This mechanism is similar to SDSA (as shown in Figure 1A) except that a non-homologous chromosome is involved. Recombination events involving similar switches between multiple chromosomes have been observed previously in yeast (54,55).

We estimated the rate of loss of the acentric chromosome by the following procedure. Cells were pre-grown on medium lacking uracil (forcing retention of the acentric chromosome) were streaked for single cells on rich growth medium. The resulting colonies were replica-plated onto medium lacking uracil. Of the 550 scored colonies, 469 were unsectored Ura^+^, 60 were sectored colonies in which the Ura^+^ and Ura^−^ sectors were of similar sizes. Assuming that the 60 colonies with similar-sized sectors were the result of chromosome loss at the first division following plating, the rate of loss is about 0.11/cell division. The rate is likely an underestimate, since some of the Ura^+^ strains may have had more than one chromosome with the *URA3* gene. Our result is consistent with the previous study of Clarke and Carbon (56) who found that only 5–10% of diploid strains with an acentric chromosome retained that chromosome after 10 generations of non-selective growth.

### CHEF gel and DNA sequence analyses of 18 independent Ura + isolates derived from SGK169

We analyzed genome DNA from the following 18 isolates by gels and whole-genome sequencing: 1, 5, 15, 17–19, 22, 32, 35, 46, 47, 62, 64, 71, 77, 81, 87 and 89; a summary of our conclusions based on this analysis are given in Supplementary Table S4. Because of different numbers of Ty elements (48), the parental W303- and YJM789-derived chromosome III homologs have different sizes, about 340 and 310 kb, respectively. Chromosomes resulting from a crossover between the two homologs may be of intermediate sizes depending on the location of the crossover. Crossovers that occur between the centromere and the pair of heterozygous Ty elements located about 30 kb from the centromere on the left arm and pairs of Ty elements located about 35 kb on the right arm would result in chromosomes of about 320 kb; each individual Ty1 or Ty2 element is about 6 kb. Based on the sizes determined by CHEF gels, coupled with Southern analysis using probes derived from chromosome III, we estimated the sizes of chromosome III in the 18 isolates. These data are summarized in Supplementary Table S4. It should be noted that some of these isolates may have more than one chromosome III of the same size.

**Figure 6.**
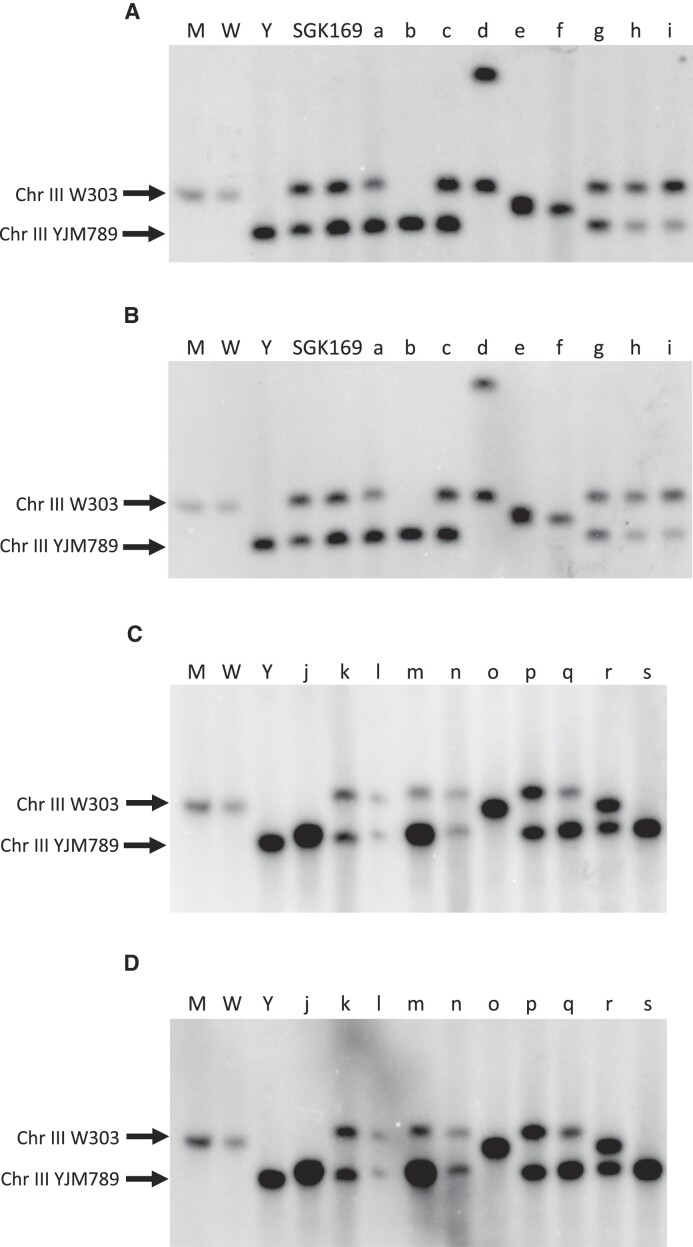
Sizes of chromosome III in SGK169 derivatives with *CEN-CEN* recombination. In 19 independent Ura^+^ derivatives (lanes a to s), we isolated DNA from cells grown in rich medium, and examined the sizes of chromosome III by CHEF gel electrophoresis. Following electrophoresis, the DNA was transferred to nitrocellulose membranes and hybridized to probes from the left (6A and 6C) and right arms (6B and 6D) of chromosome III, respectively. The sizes of the chromosome III markers are approximately 310 kb (YJM789) and 340 kb (W303). (**A**) The samples in the various lanes are labeled M (DNA size marker; Bio-Rad), W (haploid isogenic with W303), Y (haploid isogenic with YJM789), and two lanes with SGK169 DNA. The isolate numbers (Supplementary Table S3) in each lane are: 1(a), 4 (b), 5(c), 15(d), 17(e), 18(f), 19(g), 22(h) and 32(i). The Ura^+^ sample in lane b is a meiotic product as discussed in the text. The hybridization probe is a PCR fragment (generated using primers SPB1 and SPB2; Supplementary Table S2) derived from a region located near SGD coordinate 32 kb on the left arm of III. (**B**) In this gel, the lanes have the same samples as in Figure 6A. The probe, however, is a PCR fragment (generated using primers FIG2F and FIG2R; Supplementary Table S2) from the right arm of III near the SGD coordinate 268 kb. (**C**) This gel includes the isolates: 35 (j), 46(k), 47(l), 62(m), 64(n), 71(o), 77(p), 81(q), 87(r) and 89(s). The hybridization probe was the same as used in Figure 6A. (**D**) This gel includes the sample isolates as in Figure 6C, and the hybridization probe is that used in Figure 6B.

The observed sizes of the chromosomes were, in general, in good agreement with those expected from Figure 4. For example, isolate 19 was a Class 1A strain and expected to have a non-recombinant W303 homolog (340 kb) and a non-crossover YJM789 homolog with a gene conversion event at the centromere (310 kb); chromosomes of these sizes were observed by Southern analysis (Figure 6). The only isolate with a chromosome size different from 310 kb, 320 kb or 340 kb was isolate 15 which had a chromosome of about 430–440 kb. As discussed in Supplementary Table S4, this chromosome contained an intrachromosomal duplication of about 100 kb (Supplementary Figure S17).

The same 18 isolates were also examined by whole-genome sequencing (WGS). We examined the sequence coverage of every SNP in the genome, concentrating on the SNPs on chromosome III, and these data are shown in Supplementary Table S5 and Supplementary Figure S18. In these depictions, the SGD coordinates are shown on the X-axis and the Y-axis has the normalized sequence coverage (NSC). The NSC is calculated by dividing the coverage of the YJM789- or W303-derived individual SNP by the average of the coverage of the sum of all of the W303- and YJM789-derived SNPs in the genome. Thus, if there is one copy of a specific W303-derived SNP in the diploid, the NSC will be 0.5. In isolate 19, for example, the sequence analysis indicates that there is one chromosome that has only W303-derived SNPs (present in one copy) with a 5 kb region of W303-derived SNPs at the centromere that is present in multiple copies. There is another chromosome that is composed primarily of YJM789-derived SNPs with a small (5 kb) region derived from W303; this chromosome is present in multiple copies. This pattern is consistent with a gene conversion event unassociated with a crossover initiated by a DSB on the YJM789-derived homolog (Supplementary Figure S3, Class 1A), followed by amplification of the chromosome containing the conversion tract. The length of the conversion tract is defined by the size of the interstitial duplication/deletion. A diagram of all conversion events derived from SGK169 isolates is shown in Supplementary Figure S19.

Several generalizations can be made concerning the WGS data. First, the analysis supports our earlier conclusions based on PCR and coupling of markers shown in Figure 4. Second, of the 17 mitotic conversion events shown in Supplementary Figure S19, most of the conversion tracts (13 of 17) extend in one direction from the centromere (unidirectional) and the SNPs within the tract are converted from a single homolog (non-patchy). Four had contributions from both homologs. The median size of the unidirectional tracts (not including the size of the heterozygous insertions) was 5.5 kb, similar to the size of spontaneous gene conversions unassociated with crossovers in unselected wild-type yeast cells (3 to 6 kb; (45,57); the minimum, maximum, and mean tract lengths were 2, 29 and 9.8 kb, respectively. Three of the fourteen of the isolates that could be unambiguously classified (non-trisomic) had a crossover associated with the conversion event. In all 11 strains in Supplementary Figure S19 with two chromosomes that had one recombinant centromere and one non-recombinant centromere, the sequence coverage of the recombinant centromere exceeded the coverage of the non-recombinant centromere (Supplementary Figure S18). The likely explanation of this result is that the *URA3* fusion gene is expressed relatively poorly and, consequently, the chromosome containing this gene tends to get amplified in medium lacking uracil.

Since BIR is associated with high levels of mutagenesis, we examined the LOH regions on III for *de novo* mutations. No mutations on III were observed in the LOH regions, and there were only ten mutations observed in all 18 isolates. These observations do not confirm or refute the hypothesis that the LOH regions are generated by BIR, since we calculated (based on the mutation rate estimates of Elango *et al.* (58)) that only 0.2 mutations would have been expected in the LOH regions of the 18 samples (total of 560 kb).

Among the sequenced isolates resulting from mitotic recombination, we also detected multiple aneuploidy events and recombination events involving homologs other than III (Supplementary Table S6). Specifically, we observed 3 of 18 strains were trisomic for either IV, X, XII or XVI; trisomy for VIII was also observed in most of the isolates, likely the result of an event that occurred prior to selection of the *CEN-CEN* recombinant. One isolate was monosomic for chromosome X. In addition, we observed 12 different terminal LOH events and 4 different interstitial LOH events. Based on our analysis of these types of events in vegetatively growing wild-type cells (45), we calculate that the rates of aneuploidy and LOH events are elevated about 90-fold and 5-fold, respectively, in the *CEN-CEN* recombinants.

In addition to the 18 isolates described above in which the *CEN-CEN* recombinants were formed in diploid cells, we found a 19th isolate in which the recombinant centromere was in a haploid cell. In this strain, based on the sequence coverage of SNPs, all chromosomes had meiotic levels of recombination and were present in one copy per cell. We did not include the meiotic isolate in any of our subsequent analyses. In previous studies, Sherman and Roman (59) showed that induction of meiosis-specific recombination genes could occur in diploid cells, followed by a return to mitotic growth. To rule out this pathway in our experiments, we constructed a derivative of SGK169 (SGK479) that was deleted for *MATalpha*; such strains are unable to express meiosis-specific genes (60). The rate of Ura^+^ isolates in SGK479 was 1.1 × 10^−8^ (CL 0.9–1.4 × 10^−8^) /cell division, indistinguishable from the rate in the wild-type SGK169 (1.0 ± 0.14 × 10^−8^), demonstrating that the rate of *CEN-CEN* recombination does not require meiosis-specific functions.

### Aneuploidy associated with CEN-CEN recombination

27 of the Ura^+^ isolates of SGK169 that were aneuploid for chromosome III (Table 1). Class 4 isolates (9 strains) were monosomic for chromosome III containing only the RCU fragment. Class 5 isolates (18 strains) were trisomic with the PCR fragments WP and YP fragments (characteristic of the parental centromere configurations) as well as the RCU recombinant fragment. The frequencies of Class 4 and 5 isolates were 0.09 and 0.18, respectively. The very high frequency of chromosome III aneuploidy in the Ura^+^ isolates (0.27) is much higher than expected for wild-type diploids in strains in which *CEN-CEN* recombination is not selected. Sui *et al.* (45) observed a rate of aneuploidy of 4 × 10^−6^/ cell division for chromosome III in an isogenic wild-type diploid.

The high rate of chromosome non-disjunction could reflect poorly functioning centromeres of the parental chromosomes of SGK169 or the recombinant centromeres, or a disruption of the normal pattern of segregation in strains during formation of the recombinant centromere. Accordingly, we measured the rates of chromosome loss in strains with wild-type centromeres and in strains with the centromeres with the WP or YP constructs (Supplementary Figure S20). The rate measurements (details in Supp. Materials and Methods) were done by constructing strains with a wild-type *URA3* gene on the W303- or YJM789 derived chromosome III. We measured the rate of 5-FOA-resistant derivatives of these strains, and determined whether these derivatives had also lost additional heterozygous markers on chromosome III; such strains should be chromosome III monosomes (see Materials and Methods).

The rates of chromosome loss in strains with the wild-type centromeres were very low and similar for the two strains: 6.4 × 10^−6^ (CL 3.7–7.5 × 10^−6^) for the W303-derived chromosome (SGK252; Supplementary Figure S20B), and 2.0 × 10^−6^ (CL1-2.8 × 10^−6^) for the YJM789-derived chromosome (SGK276; Supplementary Figure S20C). For strains with the split *URA3* gene adjacent to the centromere, the chromosome loss rates were elevated more than 100-fold compared with the strains with the wild-type centromere: 7.4 × 10^−4^ (CL 6.0–8.6 × 10^−4^) for the W303-derived chromosome (SGK274; Supplementary Figure S20D), and 1.3 × 10^−3^ (CL1.1–1.6 × 10^−3^) for the YJM789-derived chromosome (SGK268; Supplementary Figure S20E). Although these rates of chromosome loss are very high, they are not high enough to explain the rates of loss in strains selected for centromere recombination (2.7 × 10^−1^).

We also examined the rates of chromosome loss in strains in which the chromosome had a recombinant (RCU) centromere; we found that the chromosome was further destabilized. When the recombinant centromere was on the W303-derived homolog (SGK319, Supplementary Figure S20F), the chromosome loss rate was 4.3 × 10^−2^ (CL 3.4–5.3 × 10^−2^) and the loss rate when the recombinant centromere was on the YJM789-derived chromosome (SGK325; Supplementary Figure S20G) also very high, 4.2 × 10^−2^ (CL 3.4–5.8 × 10^−2^). This observation is consistent with our sequence analysis (described above) that demonstrates that the chromosomes with the recombinant centromeres tend to become amplified relative to the monocentric chromosomes.

Although the rate of loss of chromosomes with the recombinant centromeres was very high, this observation is not sufficient to explain the high rate of aneuploidy associated with *CEN-CEN* recombination, since we select recombination events in medium lacking uracil that forces retention of the recombinant centromere. In summary, our experiments indicate a connection between *CEN-CEN* recombination and chromosome non-disjunction. Possible reasons for this association will be given in the Discussion.

### The rate of recombination between non-functional centromeres is similar to the rate observed between functional centromeres

Since the centromeres bind many different cellular proteins necessary for accurate chromosome segregation, it is possible that functional centromeres will have a different rate of breakage (higher or lower) than non-functional centromeres. To test whether the functional state of the centromere affected the rate of recombination, we constructed a diploid strain (SGK228; Supplementary Figure S20H) in which non-functional centromeres with the same flanking markers (split *URA3* gene and drug resistance genes) were inserted on chromosome III at SGD coordinate 206 kb, about 90 kb from the active *CEN3*. For this experiment, we inactivated the centromere by mutating base 106 in *CEN3* (using the centromere base numbering of Figure 3A); this substitution inactivates the centromere (2). The mutant centromere (*cen3-C106T)* was homozygous in SGK228.

The rate of recombination between inactive centromeres was 0.84 ± 0.12 × 10^−8^ recombination events/cell generation, a rate very similar to that observed for centromeres in the normal *CEN3* location (1 ± 0.14 × 10^−8^) (Figure 3C). We examined 101 Ura^+^ derivatives of SGK228 by PCR using the same primers employed previously. In general, the distribution of PCR products and the positions of breakpoints within the centromeric repeats were similar to those observed in SK169 (Figure 3B). All of the Ura^+^ isolates had the RCU (89 isolates) or RU (12 isolates) PCR fragments and most (82%) had, in addition, either the parental WP (26 of 101), YP (55 of 101) or both (2 of 101) PCR fragments. As observed for SK169, no isolate had both reciprocal recombinant products. Although both SGK169 and SGK228 had a substantial level of chromosome III aneuploidy, monosomy was much more common than trisomy in SGK228 (18 monosomes versus 2 trisomes), whereas the reverse was observed in SGK169 (10 monosomes and 18 trisomes). This difference is significant (*P* < 0.001) by the Fisher exact test).

We also sequenced the recombinant centromeres from SGK228. As we observed previously in SGK169, most (74 of 101, 73%) of the recombination breakpoints were in the 43 bp region of *CDEII* that has perfect homology between the centromere IIIs of W303 and YJM789 (Figure 3A). In summary, the rates of recombination and the types of recombination products were not substantially affected by centromere function.

### Recombination between centromeres on non-homologous chromosomes

Although recombination between centromeres on homologs can result in LOH events that include the entire arm of the chromosome, these events will not have subsequent negative effects. In contrast, recombination between the centromeres of non-homologous chromosomes will generate translocations. Heterozygous translocations in yeast lead to a high frequency of spore inviability (61–63). To examine *CEN-CEN* recombination between non-homologous chromosomes, we constructed a diploid strain (SGK260; Supplementary Figure S20I) with the *kanMX4-5*′ *ura3-5*′ *act1i-CEN3_W303_* fusion on the W303-derived homolog of III (identical to the position in SGK169), but with the *CEN3_YJM789_-3*′ *act1i-3*′ *ura3-natMX4* fragment on chromosome XI on the YJM789-derived homolog replacing the native *CEN11*. In this experiment, the centromeric sequences on III and XI are the same combination of *CEN3* sequences as used in SGK169, both derived from chromosome III. SGK260 also contained one copy each of chromosomes III and XI with the wild-type centromeres. The *CEN-CEN* recombination rate in SGK260 was 1.2 × 10^−9^ (CL 0.9–1.4 × 10^−9^), about ten-fold reduced compared to SGK169 (Figure 3C). This reduction could reflect non-random co-localization of homologs within the cell or local effects of chromosome context on the frequency of recombination.

The distribution of PCR products observed among the 76 Ura^+^ recombinants for SGK260 was substantially different than that observed in the wild-type strain in several ways (Supplementary Table S7). First, Class 1 and 2 recombinants were under-represented compared to SGK169. Second, there were three-fold more trisomic recombinants (Class 5) than observed in SGK169. Third, we found three Ura^+^ recombinants that had both products of reciprocal recombination within the centromere, resulting in balanced translocations (Figure 2). These classes were not observed in SGK169 or any of the other strains used in our study. Although the explanation for the differences between the distributions of various events in SGK260 and SGK169 is not clear, we have previously noted other differences between ectopic and allelic homologous recombination. For example, the association between gene conversion and crossovers is weaker for ectopic recombination than for allelic recombination (57).

**Figure 7.**
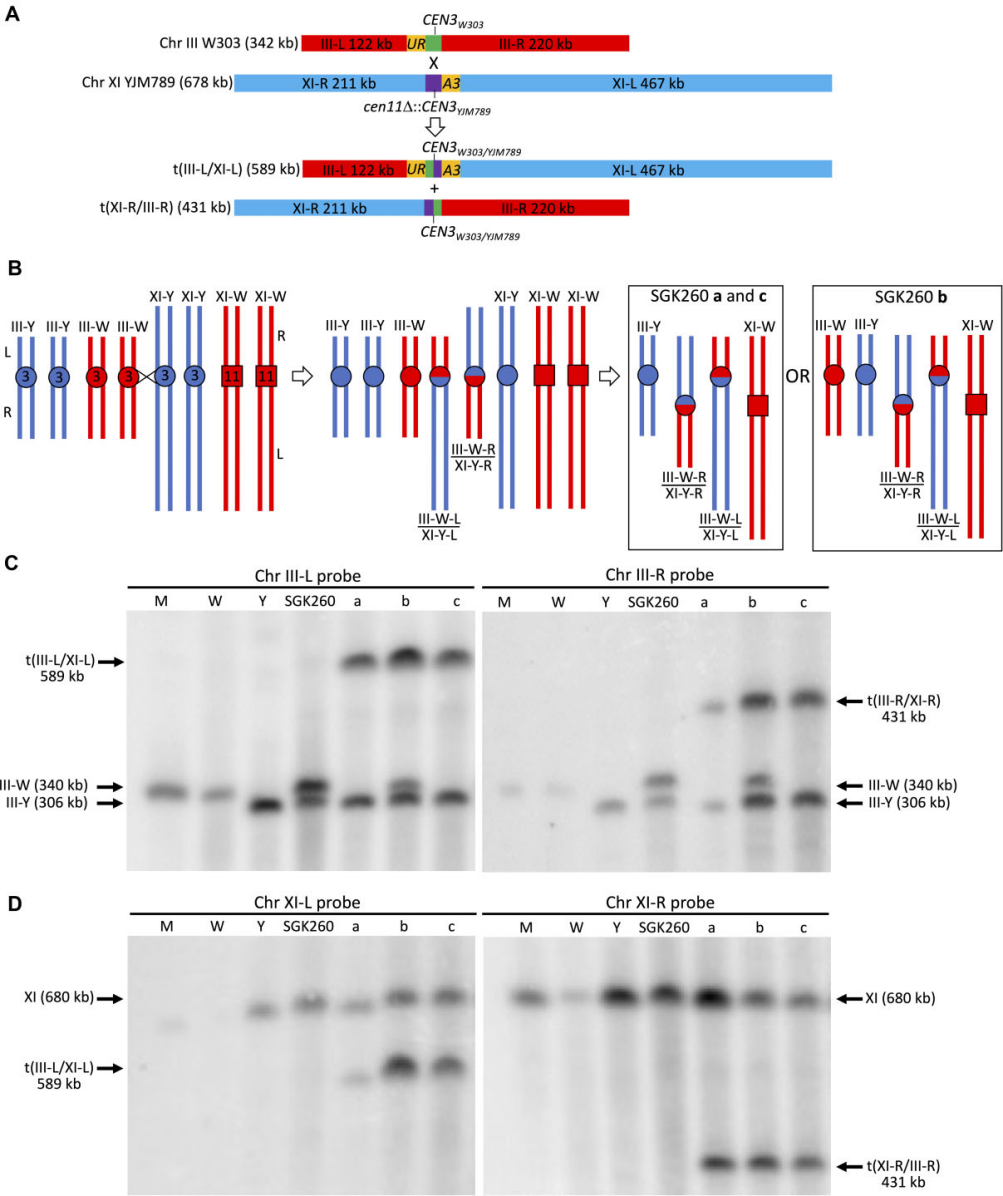
Analysis of whole-arm translocations in Ura^+^ isolates of SGK260. Ura^+^ isolates were selected in a diploid in which the W303-derived chromosome III homolog contained the 5′ end of *URA3* adjacent to *CEN3_W303_* and in which the YJM789-derived chromosome XI homolog had the 3′ end of *URA3* adjacent to *CEN3_YJM789_*. By PCR analysis, three of 76 isolates (SGK260-a, SGK260-b, and SGK260-c) had the products expected for a reciprocal crossover between the two centromeres. (**A**) Expected sizes of translocations resulting from a crossover between centromeres on chromosomes III and XI. (**B**) Depiction of recombination events that yield reciprocal translocations. Following the exchange, we show on the right side of the figure two different chromosome compositions (boxed), depending on the segregation of the chromosomes. For SGK260-a and SGK260-c, the isolates contain two reciprocal recombinants and two parental chromosomes. The SGK260-b product has both reciprocal products, one copy of the parental XI-W chromosome, and two copies of unrecombined chromosome IIIs (III-W and III-Y). Thus, this strain had a non-disjunction event in addition to the recombination. (**C**) CHEF gel and Southern analysis of putative translocations using probes for chromosome III. The hybridization probes were located near *CEN3* on the left side (left panel) and the right side (right panel), and were generated by PCR with primers cen3-L-F and cen3-L-R (left side), and cen3-R-F and cen3-R-R (right side) (Supplementary Table S2). The code for the lanes is: M (marker chromosomes, BioRad), W (isogenic with W303), Y (isogenic with YJM789), SGK260 (diploid before isolation of Ura^+^ derivatives), lanes a, b, and c (three independent Ura^+^ derivatives of SGK260, SGK260-a, SGK260-b, and SGK260-c, respectively). The gel in the left panel was probed with the Chr III-L fragment, and then re-probed with the Chr XI-R fragment (right panel of D). The gel in the right panel was probed with the Chr III-R fragment, and then re-probed with the Chr XI-L fragment (left panel of D). (**D**) CHEF gel and Southern analysis of putative translocations using probes for chromosome XI. Hybridizations to probes for the left arm (generated with primers cen11-L-F and cen11-L-R) and right arm (generated with primers cen11-R-F and cen11-R-R) are shown in the left and right panels, respectively. We used the same code for the gel lanes as in Figure 7C.

The observation of balanced translocations in some of the isolates of SGK260 was confirmed by CHEF gel analysis (Figure 7). We also examined the recombination breakpoints in SGK260 (Figure 3B). As observed in most of the other strains, most (about three-fourths) of the breakpoints were in the 43 bp region of CDEII that is identical in *CEN3* of both W303 and YJM789. In two of the three isolates with reciprocal translocations, the breakpoints in both products of the translocation were in the same 43 bp of identity in the centromeres. In the third isolate, one breakpoint was between the polymorphisms at positions 70 to 81, and the other was between positions 81 and 101 (Figure 3A). In summary, our analysis indicates that recombination between two *CEN3* centromeres on non-homologous chromosomes occurs at rates about ten-fold reduced from allelic *CEN-CEN* recombination.

We also examined recombination between a *CEN3* centromere on III, and a *CEN11* centromere on XI in the diploid SGK516 (Supplementary Figure S20J); as in other experiments, we selected Ura^+^ recombinants using the same method diagrammed in Figure 2 (details in Supp. Materials and Methods). The rate of Ura^+^ derivatives of SGK516 was very low, 5.0 × 10^−11^/cell division (CL 1–16 × 10^−11^). Only two of these derivatives were analyzed by PCR, and both contained the recombinant *URA3* gene without a centromere. To determine whether the low rate was the result of the non-allelic location of the genes, we also examined a diploid (SGK520; Supplementary Figure S20K) in which the *CEN3* and *CEN11* constructs were at allelic positions on III. The rate of Ura^+^ derivatives in this strain was also very low, 3.3 × 10^−10^ (CL 1.8–5.4 × 10^−10^). The rate of *CEN-CEN* recombination was less than the rate of Ura^+^ isolates, since all of the 16 isolates that had a recombinant *URA3* gene were acentric.

The W303- and YJM789-derived *CEN3* sequences are diverged by 6%. To test whether this degree of sequence divergence substantially reduced the rate of *CEN-CEN* recombination, we constructed a diploid isogenic with SGK169 in which the centromeric sequences were identical (SGK470). The rate of recombination in this strain was elevated about seven-fold (6.5 [5.9–7.0] × 10^−8^/division) compared to SGK169. Thus, the level of inter-centromere recombination is not greatly reduced by the low level of sequence divergence between *CEN3_W303_* and CEN3*_YJM789_*.

In summary, we failed to detect any recombination between *CEN3* and *CEN11*, likely because of sequence divergence. *CEN3* and *CEN11* have 34% sequence divergence, and the longest stretch of uninterrupted homology is 11 bp. Our finding is consistent with the lack of whole-arm translocations observed in genomic sequencing of diverse *S. cerevisiae* strains obtained from the wild (64).

## Discussion

Our analysis of *CEN-CEN* recombination in yeast has led to a number of conclusions: 1. The rate of inter-centromeric recombination between homologs in mitotically-dividing cells is about 10^−8^/cell division, 2. Most of the recombination events represent gene conversion events unassociated with crossovers, 3. Centromeric recombination is associated with very high levels of aneuploidy, 4. The rates of centromeric recombination are similar for functional and non-functional centromeres, and 5. Recombination between *CEN3_W303_* and *CEN3_YJM789_* occurs at ten-fold lower rates when these sequences are on non-homologs as on homologs. Each of these findings will be discussed below.

Although there have been few studies of the rate of mitotic recombination as a function of the length of homology, for sequences sharing 285 bp of homology with one mismatch, Jinks-Robertson *et al.* (65) found a rate of about 10^−9^/division, a rate roughly ten-fold less than we observed for the 117 bp centromeric repeats. Since the extrapolated rate of recombination for non-centromeric repeats shorter than 200 bp in the Jinks-Robertson *et al.* study (65) was zero, the centromeres have an elevated rate of recombination relative to small regions of homology analyzed previously; however, we cannot exclude the possibility that the different genetic backgrounds and/or methods used in the two studies affected the relative rates of recombination. In addition, we showed that this elevated rate does not require a functional centromere and is, therefore, more likely to be a feature of the centromeric DNA sequence. Almost all of the *CEN-CEN* recombinants had a breakpoint within the centromeric sequences (Figure 3). Although the simplest interpretation of this result is that the event was initiated by a break within the centromeric sequences, an alternative possibility is that the event was initiated in adjacent non-centromeric sequences and terminated within the centromere. We favor the first interpretation, since our previous study indicated that gene conversion events that included a marker adjacent to the centromere can be propagated through the centromere without terminating (21).

The efficiency of homologous recombination between two centromeres (46 kb apart) in a dicentric chromosome is much greater than recombination between centromeres on homologs. About 70% of dicentric chromosomes are repaired by homologous recombination (66), an efficiency more than seven orders of magnitude greater than that observed between homologs. This huge difference could be a consequence of an increased production of DSBs resulting from microtubule-induced stretching of the centromeric sequences or the creation of DSBs by cytokinesis (67). Dicentric-associated DSBs mapped physically (67) or genetically (68) occur near or at the centromeres, consistent with the observations of the current study.

None of the Ura^+^ isolates of SGK169 contained the reciprocal products of *CEN-CEN* crossovers resolved within the centromere. This result is expected since previous studies of mitotic recombination in which the recombination event is forced to occur between closely-spaced markers (for example, heteroallelic markers) are usually the consequence of gene conversion rather than crossing-over (69). In addition, in order to produce recombinant chromosomes with the crossover resolved within the centromere, the crossover would have to occur within the 117 bp homology between the interacting centromeres; short conversion tracts are rarely associated with reciprocal exchange (70). However, we point out that three Ura^+^ isolates derived from SGK260 (centromere constructs located on different homologs) had reciprocal centromeric crossovers within one cell, indicating that recombination events can (rarely) be initiated and resolved within the centromere.

An unresolved issue in our study is whether chromosomes with alterations in the coupling of flanking markers primarily reflect DSBR or BIR. Of the 25 isolates of SGK169 that had at least one chromosome with an alteration in the coupling of flanking markers, the reciprocal crossover products were observed in five strains and twenty strains had one recombinant and one non-recombinant chromosome. The isolates with reciprocal products were very likely generated by the DSBR pathway. The other isolates could be a consequence of either BIR or DSBR. If the production of crossovers is reciprocal and segregation of the recombinant products is random (50), then one would expect about half of the isolates with one crossover chromosome would have the reciprocal crossover chromosome. Since our observed distribution is different from this expectation, BIR may explain the discrepancy.

We found that about one-quarter of the *CEN-CEN* recombinants in SGK169 were aneuploid for chromosome III with high levels of both monosomes and trisomes. In two previous yeast studies of gene conversion of markers located 20–30 kb from the centromere, elevated aneuploidy was also observed (50,71); it is possible, therefore, that recombination events within the pericentric region of the chromosome may also elevate non-disjunction. Although we showed that the centromeric constructions used to detect recombination and the recombinant centromeres had elevated levels of chromosome loss, this effect does not account for the level of aneuploidy observed in the recombinants. There are several non-exclusive possibilities. First, the act of recombination may reduce *CEN3* function locally, resulting in aneuploidy. Second, the abnormal structure formed during *CEN-CEN* recombination results in a malfunction of the pericentromere of chromosome III. Since the pericentromeres of all of the chromosomes interact (bottlebrush model, discussed in (1)), a disruption of one pericentromere may destabilize the composite structure. One argument in favor of the second hypothesis is that whole-genome analysis of 18 Ura^+^ isolates identified four independent aneuploid events not involving chromosome III, a frequency about 90-fold more frequent than expected for a wild-type strain (45). It is possible that the aneuploidy affecting chromosomes other than chromosome III could affect cell viability, leading to an underestimate of the frequency of aneuploidy associated with *CEN-CEN* recombination. Lastly, it is possible that there is a sub-set of cells in a population that has a chromosome-specific abnormality that predisposes the chromosome to both recombination and non- disjunction.

In experiments in which we compared the rate of recombination between chromosome III centromeres derived from W303 and YJM789 (94% identical) on homologs and non-homologs, the rate on the non-homologs was only ten-fold less (10^−9^ compared to 10^−8^). We detected no recombination between *CEN3* and *CEN11* (66% identical) located on homologs or non-homologs (<10^−11^). This result argues that whole-arm translocations will occur very infrequently among the natural yeast chromosomes, a selective advantage since such translocations would lead to spore inviability in diploids heterozygous for such translocations. Consequently, the elevated rate of mutations observed in yeast centromeres (72) may have a selective advantage, producing centromeres on non-homologs that are extremely unlikely to recombine.

In summary, we showed that mitotic recombination can occur between centromeres, and characterized some important features of these events. Our development of a system to detect these events will allow detailed studies of their genetic regulation.

## Supplementary Material

gkad1110_Supplemental_Files

## Data Availability

The raw whole-genome sequencing data are available at the National Center for Biotechnology Information (NCBI) Sequence Read Archive (SRA) under BioProject PRJNA416056.

## References

[1] Bloom K. , Costanzo V. Centromere structure and function. Prog. Mol. Subcell. Biol. 2017; 56:515–539. 28840251 10.1007/978-3-319-58592-5_21 PMC6535225

[2] Hegemann J.H. , Shero J.H., Cottarel G., Philippsen P., Hieter P. Mutational analysis of centromere DNA from chromosome VI of *Saccharomyces cerevisiae*. Mol. Cell. Biol. 1988; 8:2523–2535. 3043181 10.1128/mcb.8.6.2523-2535.1988 PMC363453

[3] Gordon J.L. , Bryne K.P., Wolfe K.H. Mechanism of chromosome number evolution in yeast. PLoS Genet. 2011; 7:e1002190. 21811419 10.1371/journal.pgen.1002190 PMC3141009

[4] Carbon J. , Clarke L. Structural and functional analysis of a yeast centromere (*CEN3*). J. Cell. Sci. Suppl. 1984; 1:43–58. 6397474 10.1242/jcs.1984.supplement_1.4

[5] Gaudet A. , Fitzgerald-Hayes M. Alterations in the adenine-plus-thymine-rich region of *CEN3* affect centromere function in *Saccharomyces cerevisiae*. Mol. Cell. Biol. 1987; 7:68–75. 3550426 10.1128/mcb.7.1.68-75.1987 PMC365042

[6] Gaudet A. , Fitzgerald-Hayes M. Mutations in *CEN3* cause aberrant chromosome segregation during meiosis in *Saccharomyces cerevisiae*. Genetics. 1989; 121:477–489. 2653962 10.1093/genetics/121.3.477 PMC1203634

[7] Hegemann J.H. , Fleig U.N. The centromere of budding yeast. Bioessays. 1993; 15:451–460. 8379948 10.1002/bies.950150704

[8] McGrew J. , Diehl B., Fitzgerald-Hayes M. Single base-pair mutations in centromere element III cause aberrant chromosome segregation in *Saccharomyces cerevisiae*. Mol. Cell. Biol. 1986; 6:530–538. 3537689 10.1128/mcb.6.2.530-538.1986 PMC367543

[9] Jehn B. , Niedenthal R., Hegemann J.H. *In vivo* analysis of the *Saccharomyces cerevisiae* centromere CDEIII sequence: requirements for mitotic chromosome segregation. Mol. Cell. Biol. 1991; 11:5212–5221. 1922041 10.1128/mcb.11.10.5212-5221.1991 PMC361563

[10] Yamagishi Y. , Sakuno T., Goto Y., Watanabe Y. Kinetochore composition and its function: lessons from yeast. FEMS Microbiol. Rev. 2014; 38:185–200. 24666101 10.1111/1574-6976.12049

[11] Winey M. , Mamay C.L., O’Toole E.T., Mastronarde D.N., Giddings T.H., McDonald K.L., McIntosh J.R. Three-dimensional ultrastructural analysis of the *saccharomyces cerevisiae* mitotic spindle. J. Cell Biol. 1995; 129:1601–1615. 7790357 10.1083/jcb.129.6.1601 PMC2291174

[12] Altemose N. , Logsdon G.A., Bzikadze A.V., Sidhwani P., Langley S.A., Caldas G.V., Hoyt S.J., Uralsky L., Ryabov F.D., Shew C.J. et al. Complete genomic and epigenetic maps of human centromeres. Science. 2022; 376:eabl4178. 35357911 10.1126/science.abl4178 PMC9233505

[13] Sullivan L.L. , Boivin C.D., Mravinac B., Song I.Y., Sullivan B.A. Genomic size of the CENP-A domain is proportional to total alpha satellite array size at human centromeres and expands in cancer cells. Chromosome Res. 2011; 19:457–470. 21484447 10.1007/s10577-011-9208-5 PMC3565466

[14] Sundararajan K. , Straight A.F. Centromere identity and the regulation of chromosome segregation. Front. Cell Dev. Biol. 2022; 10:914249. 35721504 10.3389/fcell.2022.914249 PMC9203049

[15] Rudd M.K. , Willard H.F. Analysis of the centromeric regions of the human genome assembly. Trends Genet. 2004; 20:529–533. 15475110 10.1016/j.tig.2004.08.008

[16] Nurk S. , Koren S., Rhie A., Rautiainen M., Bzikadze A.V., Mikheenko A., Vollger M.R., Altemose N., Uralsky L., Gershman A. et al. The complete sequence of a human genome. Science. 2022; 376:44–53. 35357919 10.1126/science.abj6987 PMC9186530

[17] Sullivan B.A. , Karpen G.H. Centromeric chromatin exhibits a histone modification pattern that is distinct from both euchromatin and heterochromatin. Nat. Struct. Biol. 2004; 11:1076–1083. 10.1038/nsmb845 PMC1283111 15475964

[18] Nambiar M. , Smith G.R. Repression of harmful meiotic recombination in centromeric regions. Semin. Cell Dev. Biol. 2016; 54:188–197. 26849908 10.1016/j.semcdb.2016.01.042 PMC4867242

[19] Hartmann M. , Umbanhowar J., Sekelsky J. Centromere-proximal meiotic crossovers in *Drosophila melanogaster* are suppressed by both highly repetitive heterochromatin and proximity to the centromere. Genetics. 2019; 213:113–125. 31345993 10.1534/genetics.119.302509 PMC6727794

[20] Lambie E.J. , Roeder G.S. Repression of meiotic crossing over by a centromere (*CEN3*) in *Saccharomyces cerevisiae*. Genetics. 1986; 114:769–789. 3539697 10.1093/genetics/114.3.769 PMC1203013

[21] Symington L.S. , Petes T.D. Meiotic recombination within the centromere of a yeast chromosome. Cell. 1988; 52:237–240. 2830024 10.1016/0092-8674(88)90512-0

[22] Gerton J.L. , DeRisi J., Shroff R., Lichten M., Brown P.O., Petes T.D. Global mapping of meiotic recombination hotspots and coldspots in *Saccharomyces cerevisiae*. Proc. Natl. Acad. Sci. U.S.A. 2000; 97:11383–11390. 11027339 10.1073/pnas.97.21.11383 PMC17209

[23] Mancera E. , Bourgon R., Brozzi A., Huber W., Steinmetz L.M. High-resolution mapping of meiotic crossovers and non-crossovers. Nature. 2008; 454:479–485. 18615017 10.1038/nature07135 PMC2780006

[24] Malone R.E. , Golin J.E., Esposito M.S. Mitotic versus meiotic recombination in *Saccharomyces cerevisie*. Curr. Genet. 1980; 1:241–248. 24189665 10.1007/BF00390950

[25] Charles J. St. , Petes T.D. High-resolution mapping of spontaneous mitotic recombination hotspots on the 1.1 mb arm of yeast chromosome IV. PLoS Genet. 2013; 9:e1003434. 23593029 10.1371/journal.pgen.1003434 PMC3616911

[26] Liebman S.W. , Symington L.S., Petes T.D. Mitotic recombination within the centromere of a yeast chromosome. Science. 1988; 241:1074–1077. 3137657 10.1126/science.3137657

[27] Greenfeder S.A. , Newlon C.S. Replication forks pause at yeast centromeres. Mol. Cell. Biol. 1992; 12:4056–4066. 1508202 10.1128/mcb.12.9.4056-4066.1992 PMC360298

[28] Cha R.S. , Kleckner N. ATR homolog Mec1 promotes fork progression, thus averting breaks in replication slow zones. Science. 2002; 297:602–606. 12142538 10.1126/science.1071398

[29] Symington L.S. , Rothstein R., Lisby M. Mechanisms and regulation of mitotic recombination in *Saccharomyces cerevisae*. Genetics. 2014; 198:795–835. 25381364 10.1534/genetics.114.166140 PMC4224172

[30] Jaco I. , Canela A., Vera E., Blasco M.A. Centromere mitotic recombination in mammalian cells. J. Cell Biol. 2008; 181:885–892. 18541703 10.1083/jcb.200803042 PMC2426939

[31] Barra V. , Fachinetti D. The dark side of centromeres: types, causes and consequences of structural abnormalities implicating centromeric DNA. Nat. Commun. 2018; 9:4340. 30337534 10.1038/s41467-018-06545-y PMC6194107

[32] Hermsen M.A. , Joenje H., Arwert F., Welters M.J., Braakhuis B.J., Bagnay M., Westerveld A., Slater R. Centromeric breakage as a major cause of cytogenetic abnormalities in oral squamous cell carcinoma. Genes Chromosomes Cancer. 1996; 15:1–9. 8824719 10.1002/(SICI)1098-2264(199601)15:1<1::AID-GCC1>3.0.CO;2-8

[33] Hermsen M. , Snijders A., Guervós M.A., Taenzer S., Koerner U., Baak J., Pinkel D., Albertson D., van Diest P., Meijer G. et al. Centromeric chromosomal translocations show tissue-specific differences between squamous cell carcinomas and adenocarcinomas. Oncogene. 2005; 24:1571–1579. 15674345 10.1038/sj.onc.1208294

[34] Kim S.R. , Shaffer L.G. Robertsonian translocations: mechanisms of formation, aneuploidy, and uniparental disomy and diagnostic considerations. Genet. Test. 2002; 6:163–168. 12490055 10.1089/109065702761403315

[35] Poot M. , Hochstenbach R. Prevalence and phenotypic impact of Robertsonian translocations. Mol. Syndromol. 2021; 12:1–11. 33776621 10.1159/000512676 PMC7983559

[36] Warburton P.E. , Willard H.F. PCR amplification of tandemly repeated DNA: analysis of intra- and interchromosomal sequence variation and homologous unequal crossing-over in human alpha satellite DNA. Nucleic Acids Res. 1992; 20:6033–6042. 1461735 10.1093/nar/20.22.6033 PMC334470

[37] Donnianni R.A. , Symington L.S. Break-induced replication occurs by conservative DNA synthesis. Proc. Natl. Acad. Sci. U.S.A. 2013; 110:13475–13480. 23898170 10.1073/pnas.1309800110 PMC3746906

[38] Saini N. , Ramakrishnan S., Elango R., Ayyar S., Zhang Y., Deem A., Ira G., Haber J.E., Lobachev K.S., Malkova A. Migrating bubble during break-induced replication drives conservative DNA synthesis. Nature. 2013; 502:389–392. 24025772 10.1038/nature12584 PMC3804423

[39] Jinks-Robertson S. , Petes T.D. Mitotic recombination in yeast: what we know and what we don’t know. Curr. Opin. Genet. Dev. 2021; 71:78–85. 34311384 10.1016/j.gde.2021.07.002 PMC8671248

[40] Goldstein A.L. , McCusker J.H. Three new dominant drug resistance cassettes for gene disruption in *Saccharomyces cerevisiae*. Yeast. 1999; 15:1541–1553. 10514571 10.1002/(SICI)1097-0061(199910)15:14<1541::AID-YEA476>3.0.CO;2-K

[41] Wach A. , Brachat A., Pohlmann R., Philippsen P. New heterologous modules for classical or PCR-based gene disruptions in *Saccharomyces cerevisiae*. Yeast. 1994; 10:1793–1808. 7747518 10.1002/yea.320101310

[42] Rose M.D. , Winston F., Hieter P. Methods in Yeast Genetics: A Laboratory Course Manual. 1990; NY Cold Spring Harbor Laboratory Press.

[43] Zheng Q. rSalvador: an R package for the fluctuation experiment. G3. 2017; 7:3849–3856. 29084818 10.1534/g3.117.300120 PMC5714482

[44] Zheng D.Q. , Zhang K., Wu X.C., Mieczkowski P.A., Petes T.D. Global analysis of genomic instability caused by DNA replication stress in *Saccharomyces cerevisiae*. Proc. Natl. Acad. Sci. U.S.A. 2016; 113:e2119588119. 10.1073/pnas.1618129113 PMC5167146 27911848

[45] Sui Y. , Qi L., Wu J.K., Wen X.P., Tang X.X., Ma Z.J., Wu X.C., Zhang K., Kokoska R.J., Zheng D.Q. et al. Genome-wide mapping of spontaneous genetic alterations in diploid yeast cells. Proc. Natl. Acad. Sci. U.S.A. 2020; 117:28191–28200. 33106417 10.1073/pnas.2018633117 PMC7668089

[46] Charles J. St. , Hazkani-Covo E., Yin Y., Andersen S.L., Dietrich F.S., Greenwell P.W., Malc E., Mieczkowski P., Petes T.D. High-resolution genome-wide analysis of irradiated (UV and gamma-rays) cells reveals a high frequency of genomic loss of heterozygosity (LOH) events. Genetics. 2012; 190:1367–1284. 10.1534/genetics.111.137927 PMC3316642 22267500

[47] McCulley J.L. , Petes T.D. Chromosome rearrangements and aneuploidy in yeast strains lacking both Tel1p and Mec1p reflect deficiencies in two different mechanisms. Proc. Natl. Acad. Sci. U.S.A. 2010; 107:11465–11470. 20534547 10.1073/pnas.1006281107 PMC2895065

[48] Qi L. , Sui Y., Tang X.X., McGinty R.J., Liang X.Z., Dominska M., Zhang K., Mirkin S.M., Zheng D.Q., Petes T.D. Shuffling the yeast genome using CRISPR/Cas9-generated DSBs that the target transposable Ty1 elements. PLoS Genet. 2023; 19:e1010590. 36701275 10.1371/journal.pgen.1010590 PMC9879454

[49] Datta A. , Adjiri A., New L., Crouse G.F., Jinks-Robertson S. Mitotic crossovers between diverged sequences are required for mismatch repair proteins in *Saccharomyces cerevisiae*. Mol. Cell. Biol. 1996; 16:1085–1093. 8622653 10.1128/mcb.16.3.1085 PMC231091

[50] Chua P. , Jinks-Robertson S. Segregation of recombinant chromatids following mitotic crossing over in yeast. Genetics. 1991; 129:359–369. 1660426 10.1093/genetics/129.2.359 PMC1204629

[51] Mitchel K. , Zhang H., Welz-Voegele C., Jinks-Robertson S. Molecular structures of crossover and noncrossover intermediates during gap repair in yeast: implications for recombination. Mol. Cell. 2010; 38:211–222. 20417600 10.1016/j.molcel.2010.02.028 PMC2865147

[52] Lee P.S. , Petes T.D. Mitotic gene conversion events initiated in G1-synchronized yeast cells by gamma rays are similar to spontaneous conversion events. Proc. Natl. Acad. Sci. U.S.A. 2010; 107:7383–7388. 20231456 10.1073/pnas.1001940107 PMC2867710

[53] Liu L. , Malkova A. Break-induced replication: unraveling each step. Trends Genet. 2022; 38:752–765. 35459559 10.1016/j.tig.2022.03.011 PMC9197877

[54] Ray A. , Machin N., Stahl F.W. A DNA double chain break stimulates triparental recombination in *Saccharomyces cerevisiae*. Proc. Natl. Acad. Sci. U.S.A. 1989; 86:6225–6229. 2668958 10.1073/pnas.86.16.6225 PMC297810

[55] Anand R.P. , Tasponina O., Greenwell P.W., Lee C.S., Du W., Petes T.D., Haber J.E. Chromosome rearrangements via template switching between diverged repeated sequences. Genes Dev. 2014; 28:2394–2406. 25367035 10.1101/gad.250258.114 PMC4215184

[56] Clarke L. , Carbon J. Genomic substitutions of centromeres in *Saccharomyces cerevisiae*. Nature. 1983; 305:23–28. 6350891 10.1038/305023a0

[57] Yim E. , O’Connell K.E., Charles J. St., Petes T.D High-resolution mapping of two types of spontaneous mitotic gene conversion events in *Saccharomyces cerevisae*. Genetics. 2014; 198:181–192. 24990991 10.1534/genetics.114.167395 PMC4174931

[58] Elango R. , Osia B., Harcy V., Malc E., Mieczkowski P.A., Roberts S.A., Malkova A. Repair of base damage within break-induced replication intermediates promotes kataegis associated with chromosome rearrangements. Nucleic Acids Res. 2019; 47:9666–9684. 31392335 10.1093/nar/gkz651 PMC6765108

[59] Sherman F. , Roman H. Evidence for two types of allelic recombination in yeast. Genetics. 1963; 48:255–261. 13977170 10.1093/genetics/48.2.255 PMC1210465

[60] Kassir Y. , Simchen G. Regulation of mating and meiosis in yeast by the mating-type region. Genetics. 1976; 82:187–206. 770230 10.1093/genetics/82.2.187 PMC1213452

[61] Sherman F. , Helms C. A chromosomal translocation causing overproduction of iso-2-cytochrome *c* in yeast. Genetics. 1978; 88:689–707. 206486 PMC1213813

[62] Chaleff D.T. , Fink G.R. Genetic events associated with an insertion mutation in yeast. Cell. 1980; 21:227–237. 6250712 10.1016/0092-8674(80)90130-0

[63] Mikus M.D. , Petes T.D. Recombination between genes located on non-homologous chromosomes in *Saccharomyces cerevisiae*. Genetics. 1982; 101:369–404. 6757052 10.1093/genetics/101.3-4.369 PMC1201867

[64] Strope P.K. , Skelly D.A., Kozmin S.G., Mahadevan G., Stone E.A., Magwene P.M., Dietrich F.S., McCusker J.H. The 100-genomes strains, an *S. cerevisiae* resource that illuminates its natural phenotypic and genotypic variation and emergence as an opportunistic pathogen. Genome Res. 2015; 25:762–774. 25840857 10.1101/gr.185538.114 PMC4417123

[65] Jinks-Robertson S. , Michelitch M., Ramcharan S. Substrate length requirements for efficient mitotic recombination in *Saccharomyces cerevisiae*. Mol. Cell. Biol. 1993; 13:3937–3950. 8321201 10.1128/mcb.13.7.3937-3950.1993 PMC359934

[66] Cook D. , Long S., Stanton J., Cusick P., Lawrimore C., Yeh E., Grant S., Bloom K. Behavior of dicentric chromosomes in budding yeast. PLoS Genet. 2021; 17:e1009442. 33735169 10.1371/journal.pgen.1009442 PMC8009378

[67] Lopez V. , Barinova N., Onishi M., Pobiega S., Pringle J.R., Dubrana K., Marcand S. Cytokinesis breaks dicentric chromosomes preferentially at pericentromeric regions and telomere fusions. Genes Dev. 2015; 29:322–336. 25644606 10.1101/gad.254664.114 PMC4318148

[68] Song W. , Gawel M., Dominska M., Greenwell P.W., Hazkani-Covo E., Bloom K., Petes T.D. Nonrandom distribution of interhomolog recombination events induced by breakage of a dicentric chromosome in *Saccharomyces cerevisiae*. Genetics. 2013; 194:69–80. 23410835 10.1534/genetics.113.150144 PMC3632482

[69] Roman H. Studies of gene mutation in *saccharomyces*. Cold Spring Harbor Symp. Quant. Biol. 1956; 21:1032–1034. 10.1101/sqb.1956.021.01.015 13433590

[70] Aguilera A. , Klein H.L. Yeast intrachromosomal recombination: long gene conversion tracts are preferentially associated with reciprocal exchange and require the *RAD1* and *RAD3* gene products. Genetics. 1989; 123:683–694. 2558957 10.1093/genetics/123.4.683 PMC1203881

[71] Campbell D.A. , Fogel S. Association of chromosome loss with centromere-adjacent mitotic recombination in a yeast disomic haploid. Genetics. 1977; 85:573–585. 324869 10.1093/genetics/85.4.573 PMC1213642

[72] Bensasson D. Evidence for a high mutation rate at rapidly evolving yeast centromeres. BMC Evol. Biol. 2011; 11:211–221. 21767380 10.1186/1471-2148-11-211 PMC3155921

