# Supplementary material for: Splitting the yeast centromere by recombination
Source: Nucleic Acids Res. 2023 Nov 22;52(2):690–707. doi: 10.1093/nar/gkad1110 (PMC10810202; doi:10.1093/nar/gkad1110)
Supplement: gkad1110_Supplemental_Files [file gkad1110_supplemental_files.zip › Revised Supplemental Information.pdf]

Supplementary data for

Splitting the yeast centromere by recombination

Stanislav G. Kozmin[1], Margaret Dominska[1], Dao-Qiong Zheng[2*], Thomas D. Petes[1*]

[1]Department of Molecular Genetics and Microbiology, Duke University, Durham, NC, USA

[2]Ocean College, Zhejiang University, Zhoushan, China

Supplemental Materials and Methods

Strain constructions

The diploids used in our study were derived by crosses of haploids isogenic with W303-1A (Thomas and Rothstein, 1989) and YJM789 (Wei *et al*., 2007); the wild-type diploid is SGK169. The resulting diploids are heterozygous for approximately 55,00 SNPs enabling the high-resolution mapping of recombination events (St. Charles and Petes, 2013). In order to select recombination events within the centromere, we altered the centromeres on chromosome III as shown in Fig. 2. The W303-derived homolog had the *kanMX4* drug resistant gene, adjacent to the 5' end of *URA3* (including the promoter), with the 5' end of the *ACT1* intron adjacent to centromere sequences derived from W303 ($CEN3_{W303}$). On the YJM789-derived homolog, we had centromere sequences derived from YJM789 ($CEN3_{YJM789}$), the 3' end of the *ACT1* intron, the 3' end of *URA3*, and the *natMX4* drug resistance gene. Because the *URA3* gene is split between the two homologs, the diploid is Ura^-. Recombination between the centromeric sequences produces an intact *URA3* gene with an *ACT1* intron containing the recombinant centromere. Cells with this hybrid centromere are Ura^+.

The SGK169 diploid was constructed in multiple steps outlined in Table S1. One of the first steps was synthesis of a 1649-bp DNA fragment containing the W303-specific *URA3* promoter, the *URA3* gene disrupted with the *ACT1* intron that had an insertion of the 117bp $CEN3_{W303}$ plus 50 bp of flanking sequence embedded within the intron. This gene was synthesized and cloned into the pMK-RQ vector by Thermo Fisher Scientific (USA); the resulting plasmid was pMK-RQ-CEN3URA3. The position of the insertion of *CEN3* within the *ACT1* intron was the same as that used by Aksenova *et. al*. (2013) for the insertion of yeast telomeric DNA.

In SGK169, in the W303-derived copy of chromosome III, the 5' portion of *URA3* (coordinates 115927-116603 of V) was fused to the 5' portion of the *ACT1* intron (coordinates 54623-54686 of VI) which was fused to *CEN3* (117 bp *CEN3* plus 50 bp of adjacent sequence; coordinates 11435-114501 of III). The YJM789-derived chromosome III homolog of SGK169 had *CEN3* plus 50 bp of flanking sequence (coordinates 11435-114501 on III) fused to the 3' *ACT1* intron (54378-54622 on VI) fused to the 3' end of *URA3* (coordinates 116604-117099 on V). In the strain in which a non-functional centromere was inserted ectopically on III (SGK228), the position of the insertion was between coordinates 205999 and 206000. In strains in which *CEN3* sequences were used to replace *CEN11* sequences or *vice versa* (SGK260, SGK516, and SGK520), the coordinates for *CEN11* were 440246-440129 on XI.

Subsequent steps in the construction of the wild-type diploid SGK169 and other strains are detailed in Table S1 with primer sequences used in the constructions shown in Table S2.

Determination of recombination rates

For each experiment, yeast strains were colony-purified on plates containing rich growth medium (YEPD), and then inoculated in liquid YPD cultures. After growing for two days at 30°C, various dilutions of the cultures were plated on Synthetic-Dextrose complete (SD-complete) solid medium to measure the number of cells in the cultures (N_t) or on SD-uracil plates to measure the number of Ura^+ isolates (*r*). Since Ura^+ derivatives resulting from recombination between centromeres grew slowly in SD-uracil medium, cells were incubated for nine days before counting the number of colonies. For each genotype, the measurements were performed 3-4 times with more than twenty cultures per experiment. From the frequencies observed in independent cultures, we calculated the rates of recombination (and their 95% confidence limits) according to the Lea-Coulson maximum-likelihood method using webSalvador 0.1 software (https://websalvador.eeeeeric.com/) (Zheng, 2017). The significance of the differences in Ura^+ rates observed in strains of different genotypes was evaluated by comparison of all Ura^+ frequencies (r/N_t) observed for each genotype by the Wilcoxon-Mann-Whitney *U*-test using Prism 9 program (GraphPad Software, USA).

Physical characterization of CEN-CEN recombinants

Individual randomly-chosen Ura^+ recombinant colonies (one colony per culture) were colony-purified on SC-URA plates, inoculated into 5 ml of the same liquid medium, and incubated to stationary phase. Genomic DNA was isolated from each isolate using Zymolyase (Rose *et al.*, 1990). Two μl of genomic DNA from each sample were added to PCR mixtures containing the specific primer pairs described in Tables S1 and S2 and MyTaq Mix (Meridian Bioscience, USA). PCR were performed accordingly to manufacturer's protocol. PCR products were analyzed using agarose gel electrophoresis. PCR products obtained with ura3-intF2 and ura3-intR2 primers pair were then column-purified using the GeneJet PCR purification kit (Thermo Fisher Scientific, USA) and sequenced.

A variety of other procedures were used to characterize the SGK169 isolates (results summarized in Table S3). There are four types of centromeric configurations that we detect with the primers shown in Fig. 2: YP (sequence configuration of the YJM789 parental centromere of SGK169), WP (sequence configuration of the W303 parental centromere of SGK169), RCU (configuration of the Ura^+-containing recombinant centromere), and RU (configuration of the Ura^+ sequence devoid of centromeric sequences). In addition, we expected, but did not observe, a 270 bp PCR product expected for a reciprocal crossover within the centromere (RC, Fig. 2). We showed that isogenic diploids without the centromere-associated *URA3* and drug resistance genes produced the 270 bp PCR fragment when primers 1 and 2 were used, indicating that the RC product would have been detected if the reciprocal product was formed. The primers and

diagnostic fragments sizes for these configurations are: WP (cen3-verR and ura3-intF3, 274 bp), YP (cen3-verF and ura3-intR3, 462 bp), RCU (ura3-intF2 and ura3-intR2, 489 bp), and RU (ura3-intF2 and ura3-intR2, 372 bp). The expected size of the RC PCR product generated with primers cen3-verF and cen3-verR was 270 bp. The presence of the *MAT***a** and *MATalpha* loci was examined in the SGK169 derivatives using the primers MAT-R, MATalpha-F, and MATa-F (Table S2). Drug resistance of the isolates was determined using medium containing geneticin (for Kan^R) and nourseothricin (for Nat^R). The presence of the *leu2* gene of W303 in strains with more than one copy of chromosome III was detected by PCR using the primers Ty2-F and LEU2-R (Table S2).

As described in the main text, in order to determine the coupling of markers flanking the centromere (*MAT* and *LEU2*), in SGK169 isolates with two copies of chromosome III, we used 5-FOA to isolate monosomic derivatives that had lost the copy with the wild-type *URA3* gene. These monosomic derivatives were examined by PCR and other methods (summarized in Table S3), allowing us to determine marker coupling. The genotype at the *MAT* locus was determined using the primers MATa-F and MAT-R for the *MAT***a** locus, and MATalpha-F and MAT-R for the *MATalpha* locus (sequence of primers in Table S2).

For Southern blot analysis, Ura^+ recombinant cells were first spread on SD-URA plates, incubated 4 days at 30° C, inoculated in 7 ml of YEPD, and grown for 2 days at 30° C. Sample preparations, clamped homogeneous electric

fields gel electrophoresis, and Southern blot hybridization were performed according to McCulley and Petes (2010). Primers used to generate PCR products for chromosome III and XI hybridization probes are listed in Table S2.

For whole-genome sequence analysis, Ura^+ recombinant isolates derived from SGK169 were grown in SD-URA liquid medium to saturation. Cells were collected by centrifugation, and approximately 0.2 ml of the resulting cell pellets were sequenced on an Illumina HiSEq. 2500 sequencer using the paired-end indexing method. The mapping of the reads was done as described in Zheng *et al.* (2016) and visualized with the Prism 9 program (GraphPad Software, USA). The whole-genome SNP-microarray procedure was performed as described by St. Charles *et al*. (2012).

Determination of CEN3-modified chromosome III stability

Chromosome III stability tester strains (Table S1) were colony-purified on YPD plates, 25-30 independent 3-ml cultures per genotype were initiated from single colonies and incubated for 2 days at 30°C on rotating drum. 50 μl of 10^5-fold diluted cultures were plated on YPD plates (to determine number of viable cells per culture) and 50-100 μl of 10^2 - 10^5-fold (depending on genotype) diluted cultures were plated on FOA plates (to determine number of Ura^- clones per culture). FOA-resistant colonies were counted, and the plates were then replica-plated on additional tester plates, to score loss of a second marker on chromosome III (*MAT*alpha in SGK252; *LEU2* in SGK276; *natMX4* in SGK268; *kanMX4* in SGK274, SGK319, and SGK325).

For most of the strains described above, we could measure loss of the second marker on III by using omission medium or media that contain nourseothricin or geneticin. To measure the rate of loss of *MATalpha* in FOA^R clones of SGK252 (*ura3 LYS2*), we first replica-plated the FOA^R isolates to YPD plates containing a lawn of SGK42 cells (*MATalpha URA3 lys2*). After two days incubation, these cells were replica-plated to medium lacking lysine and uracil to determine the fraction of cells that lost the chromosome containing the *MATalpha* locus.

For all strains, using the methods described above, we determined the median frequency of 5-FOA^R isolates that lost the *URA3*-containing chromosome (f) and the total number of cells in each culture (N_t). The chromosome loss rate per generation (μ) was calculated by the Drake equation: $\mu = f/\ln(N_t\mu)$ (Drake, 1991). The 95% non-parametric confidence limits for the median f values were calculated as described by Altman (1991) and were transformed to confidence limits on the rates using the Drake formula.

Mechanisms associated with the various classes of recombinant centromeres

As described in the main text, there were five classes of centromere recombination events associated with the Ura^+ isolates of SGK169. The initial classification was based on a PCR analysis of the centromeric regions of the two homologs. The classes were then further sub-divided based on the coupling of *LEU2* and *MAT*, loci on the left and right arms of chromosome III, respectively. Although most of these classes can be explained by small modifications of the

DSBR, BIR or SDSA models shown in Figs. 1 and 5, there are several classes that involve alternative explanations. In most studies of mitotic recombination, it is assumed that two paired sister chromatids will disjoin into separate cells following recombination. In our studies, as described below, some of the events (for example, Classes 1C, 2B, and 2D) require that sister chromatids segregate into the same daughter cell (Fig. S4). In addition, although most of the events can be explained by the repair of a single DSB generated in G2, a few events (for example, Classes 1D, 1E, 2F, 2G, and 2H) appear to reflect the repair of two chromatids broken at the same position. Such isolates (for example, Class 1D, Fig. S5) have two recombinant chromosomes, one of which has a crossover and one of which has a conversion unassociated with a crossover. Analogous events have been described previously in yeast (for example, Lee and Petes, 2010) and reflect DSBs generated in G1 of the cell cycle that are replicated to yield two broken sister chromatids.

The summary of all recombinant sub-classes is shown in Fig. 4. The depictions of all sub-classes for Classes 1 and 2 are shown in Fig. 5 and Figs. S3-S15. The descriptions of Class 3-5 events are given below.

In all of the Class 3 events, there is a recombinant *URA3* gene that contains the *ACT1* intron, but lacks the recombinant centromere. Such isolates are likely to be a consequence of an ectopic recombination between the *ACT1* intronic sequences on chromosome III and the *ACT1* intron located on chromosome VI. Possible intermediates for these events are shown in Fig. S16.

Class 3A*.* This pattern could reflect the repair of a DSB on the W303-derived homolog, followed by invasion of the processed broken end into the *ACT1* intron on chromosome VI as shown in Fig. S16. After DNA synthesis extending into the 3' portion of the intron, the broken end is extruded and pairs with intronic sequences on the YJM789 chromosome. Replication of the right arm of the YJM789 chromosome by BIR would result in the observed recombinant chromosome. This chromosome segregates with an unrecombined W303 chromosome.

Class 3B. This pattern likely reflects the repair of a DSB on the W303 homolog, followed by invasion of the processed broken end into the *ACT1* intron on chromosome VI. After DNA synthesis extending into the 3' portion of the intron, the broken end is extruded and re-pairs with the other broken end of III, forming a dHJ. The resulting structure is resolved to generate a crossover, and the two crossover chromatids co-segregate.

Class 3C*.* This pattern can be explained by a broken end derived from a DSB on the YJM789 chromosome that invades the *ACT1* intron sequences on chromosome VI. Following extrusion of the invading end, it invades the W303 chromosome and copies the 5' *URA3* sequences. This end is extruded again and copies the left arm of the YJM789 chromosome by BIR. The recombinant chromosome segregates with the non-recombinant YJM789 chromosome.

Class 4 isolates are explicable by the same events described for Classes 1 and 2, followed by loss of one homolog.

Class 4A. In this sub-class, we hypothesize that the recombinant was formed by the same SDSA pathway as in Class 1A (Fig. S3). Following the recombination event, the unrecombined W303 homolog was lost.

Class 4B*.* This sub-class could be formed by a variety of pathways. One possibility is that it reflects a Class 1B event followed by loss of the unrecombined chromosome (Fig. 5B).

Class 4C. This sub-class likely arose by an event similar to that in Class 2A (Fig. 5A), followed by loss of the unrecombined YJM789 homolog.

Class 5. In this class, there are three copies of chromosome III. Because we cannot unambiguously determine the coupling relationships on all three chromosomes, we did not sub-divide this class further.

Supplemental Tables

(except Tables S3, S5, and S6 deposited as individual excel files)

Table S1. Strain construction and strain list.

Strain Name (Background)	Genotype	Construction or source
S288c	*MATα SUC2 gal2 mal2 mel flo1 flo8-1 hap1 ho bio1 bio6*	Mortimer and Johnston (1986).
W303-1B (W303)	*MATα leu2-3,112 his3-11,15 ura3-1 ade2-1 trp1-1 can1-100*	Thomas and Rothstein (1989).
YJM809 (YJM789)	*MAT***a** *ho::hisG lys2 ura3 his3Δ−200*	Obtained from J. McCusker (Duke University; isogenic with YJM789 (Wei *et al.*, 2007).
SGK6 (W303)	*MATα leu2-3,112 his3-11,15 ade2-1 trp1-1 can1-100 ura3Δ::hphMX4*	One-step transplacement of W303-1B with PCR product obtained with primers ura3reg-kanF and ura3reg-kanR and pAG32 as a template.
SGK31 (W303)	*MATα leu2-3,112 his3-11,15 ade2-1 trp1-1 can1-100*	One-step transplacement of SGK6 using PCR product

	ura3Δ::hphMX4 (*5'ura3/5'act1i/CEN3$_{W303}$/3'act1i/3'ura3)* inserted replacing wild-type *CEN3*	obtained with primers cen3-ura3-ampF and cen3-ura3-ampR, and pMK-RQ-CEN3URA3 as a template.
SGK42 (S288c)	*MAT***α** *SUC2 gal2 mal2 mel flo1 flo8-1 hap1 ho bio1 bio6 lys2Δ::hphMX4*	One-step transplacement of S288c with PCR fragment obtained with primers LYS2-kanF and LYS2-kanR using pAG32 as a template.
SGK123 (W303)	*MATα leu2-3,112 his3-11,15 ade2-1 trp1-1 can1-100 ura3Δ::hphMX4* (*kanMX4/5'ura3/5'act1i/CEN3$_{W303}$/3'act1i/3'ura3)* replacing wild-type *CEN3*	One-step transplacement of SGK31 using PCR product obtained with primers cen3-ura3-kanF and cen3-ura3-kanR, and pFA6-kanMX4 as a template.
SGK127 (W303)	*MATα leu2-3,112 his3-11,15 ade2-1 trp1-1 can1-100 ura3Δ::hphMX4* (*5'ura3/5'act1i/CEN3$_{W303}$/3'act1i/3'ura3/natMX4)* replacing wild-type *CEN3*	One-step transplacement of SGK31 using PCR product obtained with primers cen3-ura3-natF and cen3-ura3-natR, and pAG25 as a template.
SGK135 (W303)	*MATα leu2-3,112 his3-11,15 ade2-1 trp1-1 can1-100 ura3Δ::hphMX4* (*kanMX4/5'ura3/5'act1i/CEN3$_{W303}$)* inserted replacing wild-type *CEN3*	One-step transplacement of SGK6 using PCR product obtained with primers cen3-R and cen3-ura3-ampF, and SGK123 genomic DNA as a template.
SGK147 (YJM789)	*MAT***a** *ho::hisG lys2 his3Δ–200 ura3Δ::hphMX4*	One-step gene replacement of YJM809 using PCR product obtained with primers ura3reg-kanF and ura3reg-kanR, and pAG32 as a template.
SGK155 (W303)	*MATα leu2-3,112 his3-11,15 ade2-1 trp1-1 can1-100 ura3Δ::hphMX4* (*kanMX4/5'ura3/5'act1i/CEN3$_{W303}$)* replacing wild-type *CEN3* and (*CEN11$_{W303}$/3'act1i/3'ura3/natMX4)* inserted replacing wild-type *CEN11*	One-step gene replacement of SGK135 using PCR product obtained with primers cen11-ura3-natF and cen11-ura3-natR, and SGK127 genomic DNA as a template.
SGK157 (YJM789)	*MAT***a** *ho::hisG lys2 his3Δ–200 ura3Δ::hphMX4* and (*CEN3$_{YJM789}$/3'act1i/3'ura3/natMX4)* inserted replacing wild-type *CEN3*	One-step gene replacement of SGK147 using PCR product obtained using primers cen3_yjm789-ura3-natF and cen3-ura3-ampR, and SGK127 genomic DNA as a template.
SGK169 (W303/YJM789)	*MAT***a***/MATα LEU2/leu2-3,112 TRP1/trp1-1 CAN1/can1-100 ADE2/ade2-1 lys2/LYS2 his3Δ–200/his3-11,15*	Cross of SGK135 and SGK157.

	ura3Δ::hphMX4/ura3Δ::hphMX4 (CEN3$_{YJM789}$-3'act1i/3'ura3/natMX4)/(*kanMX4/5'ura3/5'act1i/CEN3$_{W303}$)*	
SGK204 (YJM789)	*MAT***a** *ho::hisG lys2 his3Δ–200 ura3Δ::hphMX4 III-57kb::URA3*	Integration of *URA3* onto III at 57 kb in SGK147 using PCR product obtained with primers III-57-ura3-F and III-57-ura3-R, and S288c genomic DNA as a template.
SGK212 (W303)	*MATα leu2-3,112 his3-11,15 ade2-1 trp1-1 can1-100 ura3Δ::hphMX4 III-206kb-(kanMX/5'ura3/5'act1i/cen3-C106T$_{W303}$)*	Insertion of a non-functional centromere on III at position 206 kb on chromosome III by transformation of SGK6 with PCR fragment produced with primers III-206-kanR and III-206-cen3C106T-kanF, and SGK123 genomic DNA as a template.
SGK215 (W303)	*MATα leu2-3,112 his3-11,15 ade2-1 trp1-1 can1-100 ura3Δ::hphMX4* (*kanMX4/5'ura3/5'act1i/CEN3$_{W303}$)* inserted replacing wild-type *CEN3; III-57kb::URA3*	Integration of *URA3* onto chr III at 57 kb in SGK135 using PCR product obtained with primers III-57-ura3-F and III-57-ura3-R, and S288c genomic DNA as a template.
SGK218 (YJM789)	*MAT***a** *ho::hisG lys2 his3Δ–200 ura3Δ::hphMX4* and (*CEN3$_{YJM789}$/3'act1i/3'ura3/natMX4)* replacing wild-type *CEN3$_{YJM789}$; III-57kb::URA3*	Integration of *URA3* onto III at 57 kb in SGK157 using PCR product obtained with primers III-57-ura3-F and III-57-ura3-R, and S288c genomic DNA as a template.
SGK224 (YJM789)	*MAT***a** *ho::hisG lys2 his3Δ–200 ura3Δ::hphMX4 III$_{YJM789}$-206kb-(cen3-C106T$_{YJM789}$/3'act1/i/3'ura3/natMX4*)	Insertion of a non-functional centromere on III at position 206 kb on YJM789-derived III homolog by transformation of SGK147 with PCR fragment produced with primers III-206-cen3C106T-natF and III-206-natR, and SGK157 genomic DNA as a template.
SGK228 (W303/YJM789)	*MAT***a***/MATα LEU2/leu2-3,112 TRP1/trp1-1 CAN1/can1-100 ADE2/ade2-1 lys2/LYS2 his3Δ–200/his3-11,15 ura3Δ::hphMX4/ura3Δ::hphMX4 III$_{YJM789}$-206kb(cen3-*	Cross of SGK212 and SGK224

	C106T$_{YJM789}$/3’act1i/3’ura3/natMX4)/III$_{W303}$-206kb-(kanMX/5’ura3/5’act1i/cen3-C106T$_{W303}$)	
SGK235 (W303)	*MATα leu2-3,112 his3-11,15 ade2-1 trp1-1 can1-100*<br>*ura3Δ::hphMX4 III-57kb::URA3*	Integration of *URA3* onto chr III at 57 kb in SGK6 using PCR product obtained with primers III-57-ura3-F and III-57-ura3-R, and S288c genomic DNA as a template.
SGK239 (YJM789)	*MAT**a** ho::hisG lys2 his3Δ–200 ura3Δ::hphMX4 (CEN3$_{YJM789}$/3’act1i/3’ura3/natMX4)* replacing wild-type *CEN11*	One-step gene replacement of SGK147 using PCR product obtained with primers cen3-nat-cen11-L-yjm and cen3-chrXIyjm-ura3-natR, and SGK127 genomic DNA as a template.
SGK252 (W303/YJM789)	*MAT**a**/MATα LEU2/leu2-3,112 TRP1/trp1-1 CAN1/can1-100 ADE2/ade2-1 lys2/LYS2 his3Δ–200/his3-11,15 ura3Δ::hphMX4/ura3Δ::hphMX4 III$_{YJM789}$-57kb/III$_{W303}$-57kb::URA3*	Cross of SGK147 and SGK235
SGK260 (W303/YJM789)	*MAT**a**/MATα LEU2/leu2-3,112 TRP1/trp1-1 CAN1/can1-100 ADE2/ade2-1 lys2/LYS2 his3Δ–200/his3-11,15 ura3Δ::hphMX4/ura3Δ::hphMX4 CEN3$_{YJM789}$/ (kanMX4/5’ura3/5’act1i/CEN3$_{W303}$) (CEN3$_{YJM789}$/3’act1i/3’ura3/natMX4)/CEN11$_{W303}$*	Cross of SGK239 and SGK135
SGK268 (W303/YJM789)	*MAT**a**/MATα LEU2/leu2-3,112 TRP1/trp1-1 CAN1/can1-100 ADE2/ade2-1 lys2/LYS2 his3Δ–200/his3-11,15 ura3Δ::hphMX4/ura3Δ::hphMX4 (CEN3$_{YJM789}$/3’act1i/3’ura3 /natMX)/CEN3$_{W303}$) III$_{YJM789}$-57kb::URA3/III$_{W303}$-57kb*	Cross of SGK6 and SGK218
SGK274 (W303/YJM789)	*MAT**a**/MATα LEU2/leu2-3,112 TRP1/trp1-1 CAN1/can1-100 ADE2/ade2-1 lys2/LYS2 his3Δ–200/his3-11,15 ura3Δ::hphMX4/ura3Δ::hphMX4 CEN3$_{YJM789}$/(kanMX4/5’ura3/5’act1i/CEN3$_{W303}$) III$_{YJM789}$-57kb/III$_{W303}$-57kb::URA3*	Cross of SGK147 and SGK215
SGK276 (W303/YJM789)	*MAT**a**/MATα LEU2/leu2-3,112 TRP1/trp1-1 CAN1/can1-100 ADE2/ade2-1 lys2/LYS2 his3Δ–200/his3-11,15 ura3Δ::hphMX4/ura3Δ::hphMX4 III$_{YJM789}$-57kb::URA3/III$_{W303}$-57kb*	Cross of SGK6 and SGK204
II-171-4-6 (W303/YJM789)	*MAT**a**/MATα TRP1/trp1-1 CAN1/can1-100 ADE2/ade2-1 lys2/LYS2 his3Δ–200/his3-11,15 ura3Δ::hphMX4/ura3Δ::hphMX4 (kanMX4/5’ura3/5’act1i/CEN3$_{W303/YJM789}$*	Spontaneous Ura^{+} monosomic III derivative of SGK169. III$_{W303}$ homolog has RCU.

	3'act1i/3'URA3/natMX4)	
SGK307 (W303)	*MATα leu2-3,112 his3-11,15 ade2-1 trp1-1 can1-100 ura3Δ::hphMX4 III-57kb::URA3* (*kanMX4/5'ura3/5'act1i/CEN3*$_{W303/YJM789}$ *3'act1i/3'URA3/natMX4*) replacing (*kanMX4/5'ura3/5'act1i/CEN3*$_{W303}$) on III	One-step gene integration of SGK215 using PCR product obtained with primers ura3-intF and cen3-verR, and II-171-4-6 genomic DNA as a template.
SGK311 (YJM789)	*MAT***a** *ho::hisG lys2 his3Δ–200 ura3Δ::hphMX4 III-57kb::URA3* (*kanMX4/5'ura3/5'act1i/CEN3*$_{W303/YJM789}$ *3'act1i/3'URA3/natMX4*) replacing (*CEN3*$_{YJM789}$*/3'act1i/3'ura3/natMX4)*	One-step gene integration of SGK218 using PCR product obtained with primers ura3-intR and cen3-verF, and II-171-4-6 genomic DNA as a template.
SGK319 (W303/YJM789)	*MAT***a***/MATα LEU2/leu2-3,112 TRP1/trp1-1 CAN1/can1-100 ADE2/ade2-1 lys2/LYS2 his3Δ–200/his3-11,15 ura3Δ::hphMX4/ura3Δ::hphMX4 CEN3*$_{YJM789}$*/ (kanMX4/5'ura3/5'act1i/CEN3*$_{W303/YJM789}$ */3'act1i/3'URA3/natMX4) III*$_{YJM789}$*-57kb/III*$_{W303}$*-57kb::URA3*	Cross of SGK147 and SGK307
SGK325 (W303/YJM789)	*MAT***a***/MATα LEU2/leu2-3,112 TRP1/trp1-1 CAN1/can1-100 ADE2/ade2-1 lys2/LYS2 his3Δ–200/his3-11,15 ura3Δ::hphMX4/ura3Δ::hphMX4 (kanMX4/5'ura3/5'act1i/CEN3*$_{W303/YJM789}$ */3'act1i/3'URA3/natMX4)/ CEN3*$_{W303}$ *III*$_{W303}$*-57kb/III*$_{YJM789}$*-57kb::URA3.*	Cross of SGK6 and SGK311
SGK449 (YJM789)	*MAT***a** *ho::hisG lys2 his3Δ–200 ura3Δ::hphMX4 (CEN11*$_{W303}$*/3'act1i/3'ura3/natMX4)* replacing *CEN3*$_{YJM789}$	One-step transplacement of SGK147 using PCR product obtained with primers cen3-ura3-natR and cen3-cen11w-ura3-natF and SGK155 genomic DNA as template
SGK466 (YJM789)	*MAT***a** *ho::hisG lys2 his3Δ–200 ura3Δ::hphMX4 (CEN3*$_{W303}$*-3'act1i/3'ura3/natMX4*) replacing wild-type *CEN3*	One-step transplacement of SGK147 with PCR product obtained using primers yjm789-cen3_W303-natF and cen3-ura3-ampR and SGK127 genomic DNA as a template
SGK470 (W303/YJM89)	*MAT***a***/MATα LEU2/leu2-3,112 TRP1/trp1-1 CAN1/can1-100 ADE2/ade2-1 lys2/LYS2 his3Δ–200/his3-11,15 ura3Δ::hphMX4/ura3Δ::hphMX4 (CEN3*$_{W303}$*-3'act1i/3'ura3/natMX4*)/(*kanMX4/5'ura3/5'act1i/CEN3*$_{W303}$)	Cross of SGK135 and SGK466

SGK479 (W303/YJM789)	*MAT**a**/matα::HIS3 LEU2/leu2-3,112 TRP1/trp1-1 CAN1/can1-100 ADE2/ade2-1 lys2/LYS2 his3Δ-200/his3-11,15 ura3Δ::hphMX4/ura3Δ::hphMX4 (CEN3*$_{YJM789}$*/3'act1i/3'ura3/natMX4)/(kanMX4/5'ura3/5'act1i/CEN3*$_{W303}$*)*	One-step gene replacement of SGK169 using PCR product obtained with primers mat-his3F and mat-his3R, and s288c genomic DNA as a template.
SGK516 (W303/YJM789)	*MAT**a**/MATα lys2/LYS2 LEU2/leu2-3,112 his3Δ–200/his3-11,15 ura3Δ::hphMX4/ura3-1 ADE2/ade2-1 TRP1/trp1-1 CAN1/can1-100 CEN11*$_{YJM789}$*/(CEN11*$_{W303}$*/3'act1i/3'ura3/natMX4) CEN3*$_{YJM789}$*/(kanMX4/5'ura3/5'act1i/CEN3*$_{W303}$*)*	Cross of SGK147 and SGK155
SGK520 (W303/YJM789)	*MAT**a**/MATα LEU2/leu2-3,112 his3Δ–200/his3-11,15 ura3Δ::hphMX4/ura3Δ::hphMX4 ADE2/ade2-1 TRP1/trp1-1 CAN1/can1-100 (CEN3Δ::CEN11*$_{W303}$*/3'act1i/3'ura3/natMX4)/ (kanMX4/5'ura3/5'act1i/CEN3*$_{W303}$*)*	Cross of SGK449 and SGK135

Table S2. Names and sequences of oligonucleotides used in the study.[1]

**Names of oligonucleotides**	**Sequences of oligonucleotides (5′ to 3′)**
ura3reg-kanF	ATTTATGGTGAAGGATAAGTTTTGACCATCAAAGAAGGTTAATGTGGCTGCAGCTGAAGCTTCGTACGC
ura3reg-kanR	AGCTTTTTCTTTCCAATTTTTTTTTTTTCGTCATTATAGAAATCATTACGACCGAGATTCCAGGCCACTAGTGGATCTG
cen3-ura3-ampF	AGCGCCAAACAATATGGAAAATCCACAGAAAG
cen3-ura3-ampR	AGTAAACGTTTCATATATCCATTCAATG
cen3-ura3-kanF	TCAAAAAAAAGAACAAAAAAAAAAAAGATGAATTGAAAAGCTTTATGGACCCTGAAACCACAGCTGAAGCTTCGTACGC
cen3-ura3-kanR	AGCGCCAAACAATATGGAAAATCCACAGAAAGCTATTCATTGAAAAAATAGTACAAATAAAGGCCACTAGTGGATCTG
cen3-ura3-natF	ATGCATGTATACTAAACTCACAAATTAGAGCTTCAATTTAATTATATCAGTTATTACCCGCAGCTGAAGCTTCGTACGC
cen3-ura3-natR	AGTAAACGTTTCATATATCCATTCAATGAAATATATATTTCTTACTATTTCTTAGGCCACTAGTGGATCTG
cen3-R	TTTTAACTTTCGGAAATCAAATAC
cen11-ura3-natF	TTATATTTTTTTAATTACATAATCATAAAAATAAATGTTCATGATTTCCGAACGTATAAAGAGAGATTTCTCTTTTACCT
cen11-ura3-natR	TTTCTATGTGTATTTGATTATTTTAATTACCAAGAAAACAAATTCTCGTAACATTCTTATAGGCCACTAGTGGATCTG
cen3_yjm789-ura3-natF	AGTTTATTTTCAGAAAATAAAATGTAAAATATTAGTGTATTTGTTTTCCGAAAGTTAAAAGAGAGATTTCTCTTTTACCT

III-57-ura3-F	AAAAAGCAGGTGGTTTAAGAATTGCGCAGGGAGAAGAAAGAATATCATATCTCATGATGGTGGTTTCAGGGTCCATAAAG
III-57-ura3-R	TTTTAAGATATAAAAATACTAGCCTCTTCTCACTTCGTTTTCTCTCTGTCTAAGTGGCTACCGGGTAATAACTGATATAAT
III-206-cen3C106T-kanF	GTTGAAAATAATAAAATGATTAACTATTGACAGAGGGGTAAGTGGGGTTGTTACCTTTTTTTTAACTTTCAGAAATCAAATAC
III-206-kanR	TTTATATAACTATAGCTTTTTTTATGTTATAAGAAAACATTTATTCCTTTAACCAAAAGGAGGCCACTAGTGGATCTG
III-206-cen3C106T-natF	TATAGCTTTTTTTATGTTATAAGAAAACATTTATTCCTTTAACCAAAAGGGTCACATGATAAAATTTGATTTTTTTATATTT TTAAAAAAAGTAAAAAATAAAAAGTAGTTTATTTTCAGAAAATAAAATGTAAAATATTAGTGTATTTGTTTTCTGAAAGTTAAAA
III-206-natR	GTTGAAAATAATAAAATGATGAACTATTGACAGAGGGGTAAGTGGGGTTGTTACCTTTTTAGGCCACTAGTGGATCTG
cen3-nat-cen11-L-yjm	CATTGTATTGATTGATTCTGCATTTATCTTTCTCTTGATCCGTAAATATCATTTTCATAAGTCACATGATAAAATTTGATTTTTT
cen3-chrXIyjm-ura3-natR	TTTCTATGGTTATTTGATTATTTTAATTACCAAGAAAACAAATTCTCGTAACATTCTTATAGGCCACTAGTGGATCTG
URA3-intF	TTGATGTTAGCAGAATTGTC
URA3-intR	CTAATGCTTCAACTAACTCC
CEN3-verF	ATAAACATGGCATGGCGATC
CEN3-verR	ATATGAGCAAAACTTCCACC
cen3-cen11w-ura3-natF	AGCGCCAAACAATATGGAAAATCCACAGAGAGCTATTCATAAAAAAAATAGTACAAATAAGTCACATGATAAAAACATATTT
yjm789-cen3_W303-natF	AACATGGCATGGCGATCAGCGCCAAACAATATGGAAAATCCACAGAGAGCTATTCATAAAAAAAATAGTACAAATAAGTCACATGAT
mat-his3F	CTTCACTTTTTATGAAATGTATCAACCATATATAATAACTTAATAGACGACATTCACAATTGGCCTCCTCTAGTACACTC
mat-his3R	ATAGCTATACTGACAACATTCAGTACTCGAAAGATAAACAACCTCCGCCACGACCACACTTACTTACTGACATTCATAGG
ura3-intF2	GGCGGAAGAAGTAACAAAGG
ura3-intF3	GTAACAAAGGAACCTAGAGG
ura3-intR2	CCCTTGCATGACAATTCTGC
ura3-intR3	ATTCTGCTAACATCAAAAGG
CEN11-verF	ACCTAATACCTCAATGGTCC
CEN11-verR	TCTGTAATCACTTGTCAACG
III-206-verF	ATAACCATGGAATATGTTTC
III-206-verR	ATATGTATATGTCTACATAGAG
MAT-R	ATGCACATCAAGATCGTTTATGG
MATalpha-F	GCACGGAATATGGGACTACTTCG
MATa-F	ACTCCACTTCAAGTAAGAGTTTG
Ty2-F	GACCAAGAAGAACATTGCTGATG
LEU2-R	CGATCTTCTTAGGGGCAGACAT
III-169-F	TAGAGAAACCACCAGTAGCG
FS1-S1	GTTTCTAAAACCAATATTCGC

SPB1F	CGAAGGTTGTTATTGATCTGTG
SPB1R	CGTCGTCCTTAACTTCGATGC
FIG2F	CAACTGGCGAAGTGCAGTATTC
FIG2R	GGTAATGACGATGTGGCGTCAAC
cen3-L-F	CCCATTGTTCTAGTTCTACA
cen3-L-R	GCAAAGAAGACAAGTAATCC
cen3-R-F	GCGATTTAGAGCAATCATTG
cen3-R-R	ATTATTTCAGGTACTCTGGC
cen11-L-F	GTGCTTGGAGAAATTATTCC
cen11-L-R	CTATGCATGTTTAGAGCAAG
cen11-R-F	CTAGCTTCCACGTATACAAG
cen11-R-R	AGTTATGGAAGAGAAATCAGG

[1]The specific oligonucleotides used for each strain construction are described in Table S1. Red font shows the names and oligonucleotides used to generate hybridization probes or analysis of phenotypes of 5-FOA-resistant isolates.

Table S4. Results of sequence and CHEF gel analyses of 18 SGK169 Ura$^+$ isolates.

Isolate	Class	PCR fragments	Approximate size(s) of chromosome III(s) (kb)	Conversion tract: size (kb) and directionality	CO/NCO
SGK169	Control	WP, YP	310, 340	No conversion tract	No crossover
1	2A	YP, RCU	310, 340	29 (BI, patchy)	NCO
5	5	WP, YP, RCU	310, 340	12 (UNI)	ND
15	3A	WP, RU	340, 430-440	Complex pattern	Complex CO
17	2C	YP, RCU	320, 330	15 (UNI)	CO
18	4B	RCU	320	17 (UNI, patchy)	CO
19	1A	WP, RCU	310, 340	5 (UNI)	NCO
22	1A	WP, RCU	310, 340	4 (UNI)	NCO
32	1A	WP, RCU	310, 340	5 (UNI)	NCO

35	2D	YP, RCU	310	4 (UNI)	NCO
46	2A	YP, RCU	310, 340	3 (UNI)	NCO
47	1B	WP, RCU	310, 340	25 (UNI, patchy)	NCO
62	2A	YP, RCU	310, 340	2 (UNI)	NCO
64	1A	WP, RCU	310, 340	5 (UNI)	NCO
71	3B	YP, RU	320, 330	15 (UNI)	CO
77	5	WP, YP, RU	340, 310	4 (UNI)	ND
81	5	WP, YP, RCU	340, 310	6 (UNI)	ND
87	1D	WP, RCU	310, 330	Complex pattern	ND
89	2D	YP, RCU	310	6 (UNI)	NCO

Key to abbreviations. The numbers in the first column correspond to the isolate (Clone) number in Table S3. The classes shown in the second column are shown in Fig. 4. The structure of the centromeres as defined by PCR (third column) are: WP (parental W303 centromere), YP (parental YJM789 centromere), RCU (recombinant centromere and recombinant *URA3* gene), RU (recombinant *URA3* gene lacking a centromere). Chromosome III sizes (fourth column) were measured by CHEF gel analysis. The abbreviations UNI and BI (fifth column) indicate whether the gene conversion tract extended from the centromere into only one chromosome arm (UNI, for example, Isolate 5 in Fig. S19) or into both chromosome arms (BI, for example, Isolate 1 in Fig. S19). The last column indicates whether the conversion event was associated with a crossover of flanking markers (CO) or unassociated with a crossover (NCO). For certain isolates, no unambiguous class could be assigned (ND).

Table S7. Numbers (frequencies in parentheses) of various classes of Ura$^+$ recombinants in SGK169, SGK228, SGK260, and SGK470.

**Strain**[1]	**Class 1 (WP, RCU)**	**Class 2 (YP, RCU)**	**Class 3 (RU)**	**Class 4 (RCU)**	**Class 5 (RCU + WP + YP)**	**Class 6 (RCU + RC)**[2]	**Total analyzed**
SGK169	31 (0.31)	39 (0.39)	3 (0.03)	9 (0.09)	18 (0.18)	0	100
SGK228	21 (0.21)	52 (0.51)	12 (0.12)	14 (0.14)	2 (0.02)	0	101
SGK260	8 (0.11)	21 (0.28)	5 (0.07)	4 (0.05)	35 (0.46)	3 (0.04)	76
SGK470	11 (0.22)	19 (0.37)	3 (0.06)	16 (0.31)	2 (0.04)	0	51

[1]SGK169: *kanMX4-5'ura3-5'act1-CEN3*$_{W303}$ on III$_{W303}$ and *CEN3*$_{YJM789}$*-3'act1-3'ura3-natMX4* on III$_{YJM789}$.
SGK228: *kanMX4-5'ura3-5'act1-cen3-C106T*$_{W303}$ at 206 kb on III$_{w303}$; *cen-C106T*$_{YJM789}$*-3'act1-3'ura3-natMX4* at 206 kb on III$_{YJM789}$.
SGK260: *kanMX4/5'ura3/5'act1i/CEN3*$_{W303}$ on III$_{W303}$; *CEN3*$_{YJM789}$*/3'act1i/3'ura3/natMX4* on XI$_{YJM789}$.
SGK470: *kanMX4/5'ura3/5'act1i/CEN3*$_{W303}$ on III$_{W303}$; *CEN3*$_{W303}$*-3'act1i/3'ura3/natMX4* on III$_{YJM789}$.

[2]Classes 1-5 defined in the text. Class 6 isolates have reciprocal products of *CEN-CEN* recombination (Fig. 2).

Supplemental Figures

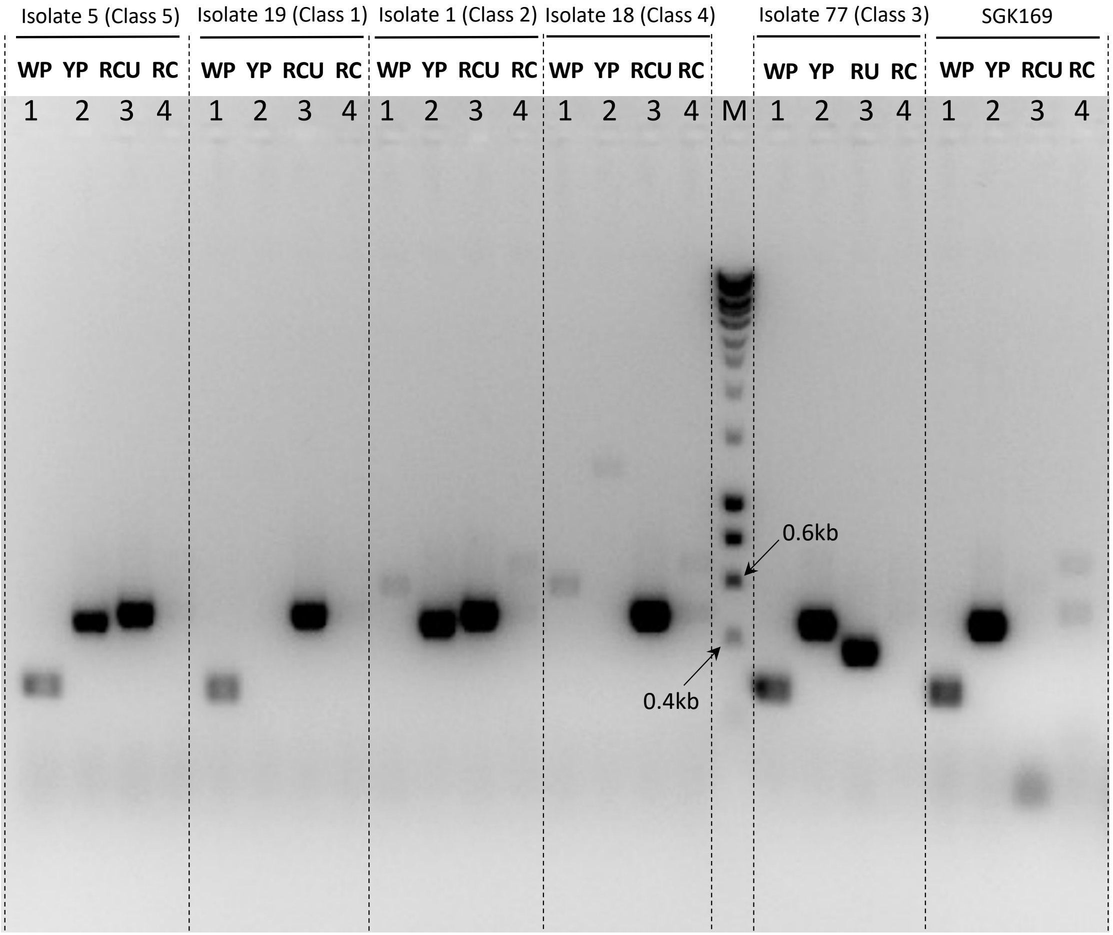

Fig. S1. PCR analysis of centromere configurations in SGK169 Ura^+ isolates. Using the primers described in the text and shown in Fig. 2, we performed PCR with genomic DNA of various Ura^+ isolates. The expected sizes of the five diagnostic PCR fragments are: WP (274 bp), YP (462 bp), RCU (489 bp), and RU (372 bp); the RC fragment, which was not observed in these isolates, has an expected size of 270 (bp). The Class 5 isolate (trisomic strain) shows fragments diagnostic of WP, YP, and RCU. Isolate 5 (Class 5 strain) has the fragments expected for the WP parental chromosome, and a chromosome with a recombinant centromere (RCU), whereas Isolate 19 (a Class 2 isolate) has the fragments expect for the non-recombinant YJM789 chromosome (YP) and a chromosome with the recombinant centromere (RCU). Isolate 18 (Class 4) is a monosomic strain that has a chromosome with the recombinant centromere (RCU). Isolate 77 (Class 5) is a trisomic strain in which the recombinant *URA3* gene lacks a centromere resulting in the RU fragment, in addition to the WP and YP fragments. Lastly, SGK169 (the parental diploid) has the WP and YP parental fragments and no recombinant fragment.

A. Configurations of markers in chromosomes without recombinant centromeres.

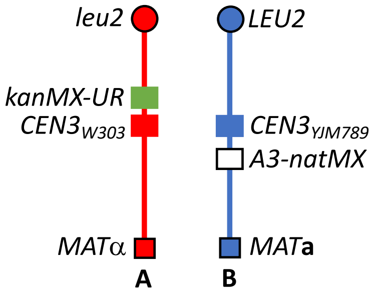

B. Configurations of markers in chromosomes with recombinant centromeres and/or recombinant *URA3* genes.

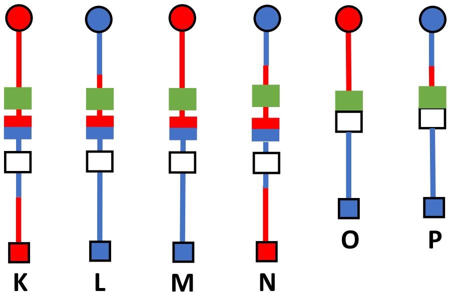

Fig. S2. Diagrams of chromosome III homologs in Ura^{+} derivatives of SJK169. We used PCR and other methods to examine the configuration of the centromeres and the coupling of flanking markers. W303 and YJM789 sequences are shown in red and blue, respectively. The *LEU2* and *MAT* loci are designated by circles and arrows, respectively. The green and white rectangles represent the 5' and 3' ends of the *URA3* gene, and the red and blue rectangles indicate the centromeric sequences; the *ACT1* intron is not shown in this figure.

A. The five types of chromosomes with the parental (non-recombinant) centromeres.

B. The six types of chromosomes with recombinant *URA3* gene with recombinant centromeres (K-N) or acentric recombinant *URA3* genes (O and P).

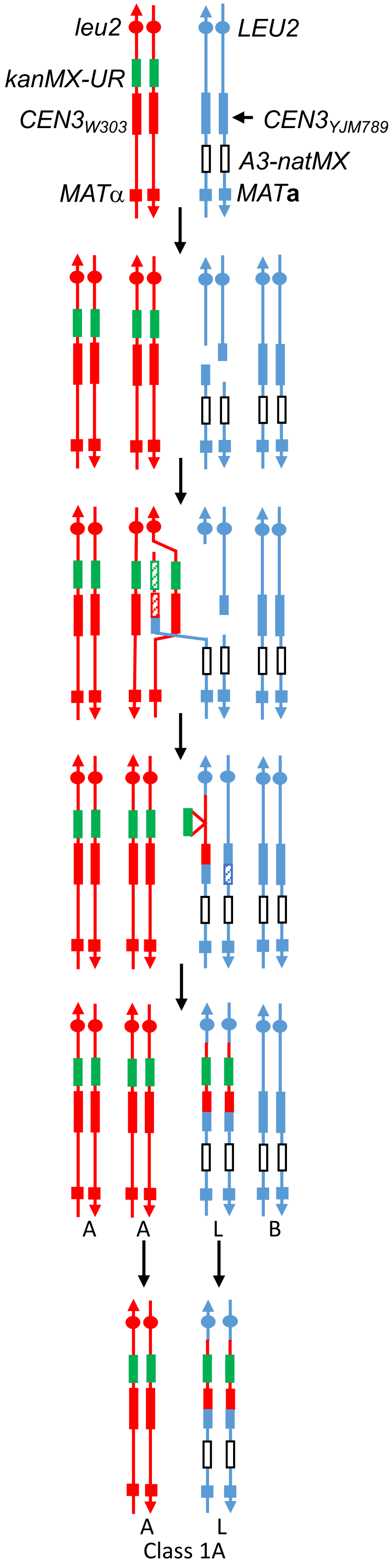

Fig. S3. Class 1A. In all Figs. S3-S15, the chromosomes are shown as double-stranded molecules with arrows marking the 3' ends. The W303- and YJM789-derived homologs are shown as red and blue lines, respectively. The *LEU2* and *MAT* loci are shown as circles and squares respectively, and the centromeres are represented by rectangles. The *kanMX-5' URA3* cassette is shown as a green rectangle and the *3' URA3-natMX* cassette is indicated by a blue rectangle; the *ACT1* intron is not depicted in this diagram.

In Class 1A, the DSB occurs at the centromere of the YJM789 homolog, followed by strand invasion into the intact W303 homolog. Following DNA synthesis and copying of a portion with the W303 centromere, the 5' portion of the *ACT1* intron, the 5' ura3 sequence, and the *kanMX4* gene, the invading strand is displaced and reassociates with the YJM789 homolog (SDSA pathway, Figs. 1A).

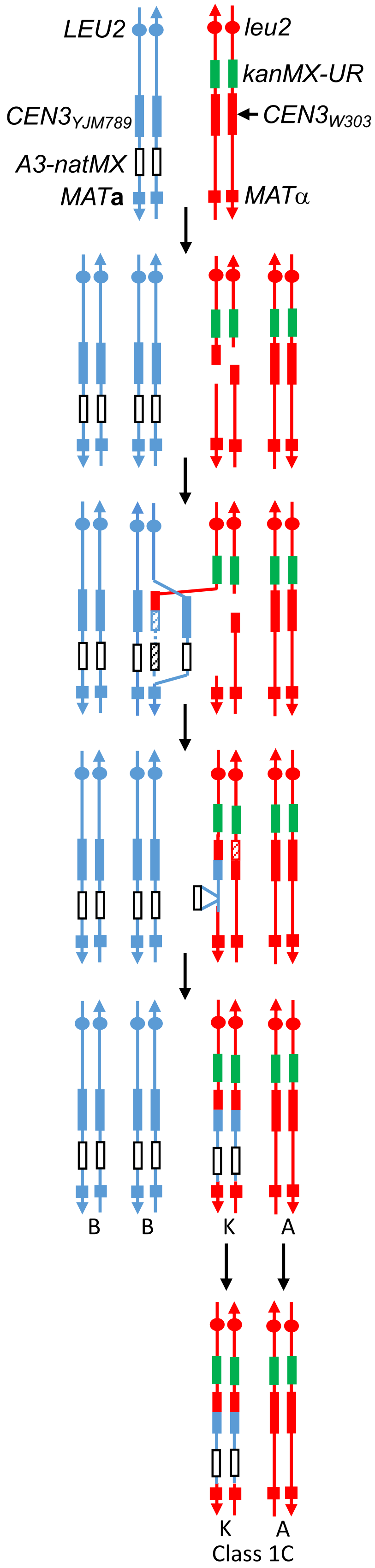

Fig. S4. Class 1C. This event is consistent with SDSA with the event initiated on the W303 homolog. This pathway results in a "K" chromosome (Fig. S2). The other chromosome has the markers of the W303 parental "A" chromosome. To obtain the co-segregation of these two chromosomes, we require that the unrecombined sister chromatid of W303 co-segregate with the recombinant chromatid. (SDSA pathway + non-disjunction).

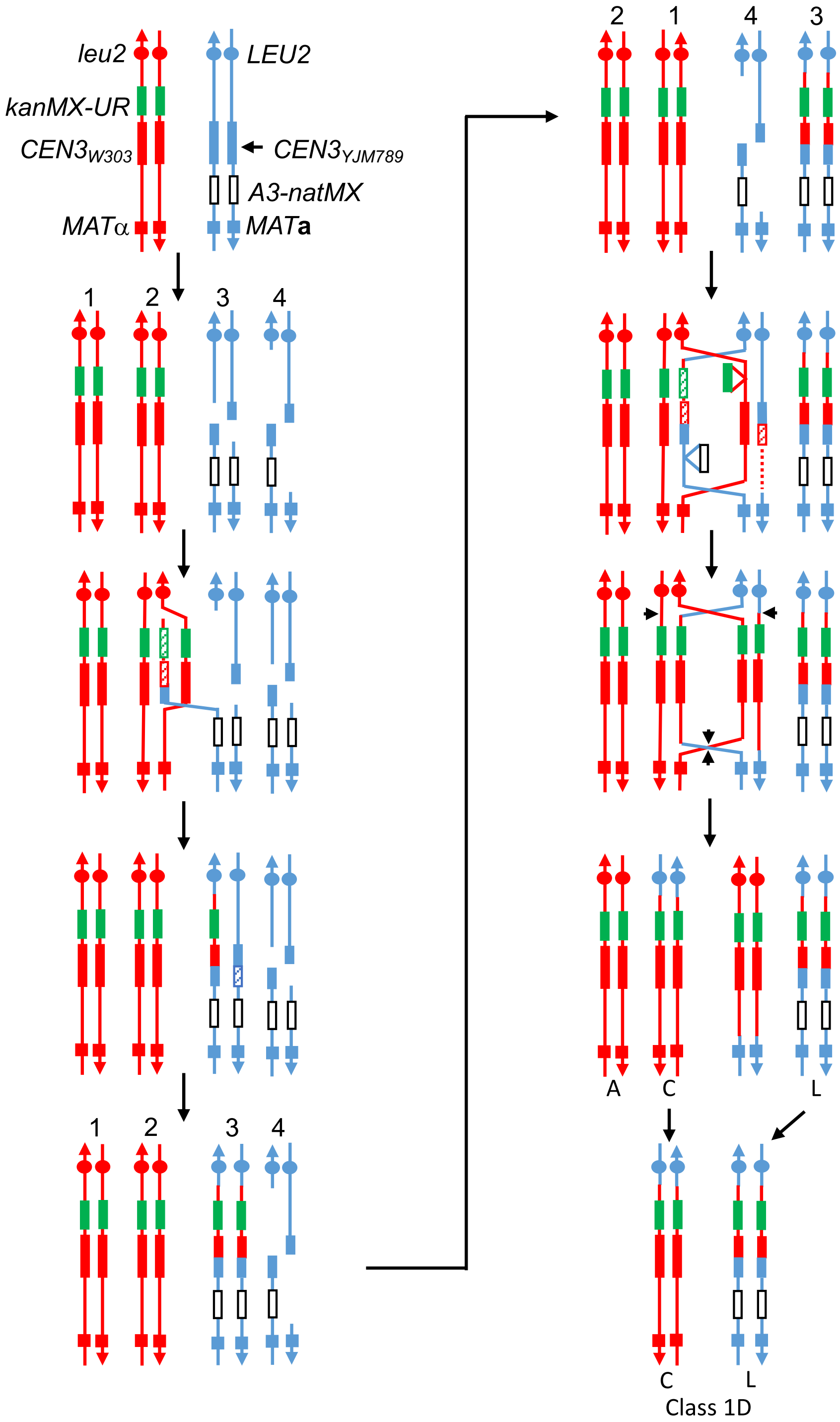

Fig. S5. Class 1D. In this class, the chromosome with the recombinant centromere has the flanking markers in the NCO configuration whereas the chromosome with the parental WP chromosome has the flanking markers in the recombinant configuration. This pattern could reflect repair of a G1-initiated DSB in the YJM789 chromosome that results in two YJM789 chromatids broken at the same positions. Repair of one chromatid (labeled "L") occurs by SDSA unassociated with a crossover (left side), and the repair of the second (labeled "C") occurs by the DSBR pathway associated with a crossover (right side). Chromosomes 1 and 3 disjoin into the same cell.

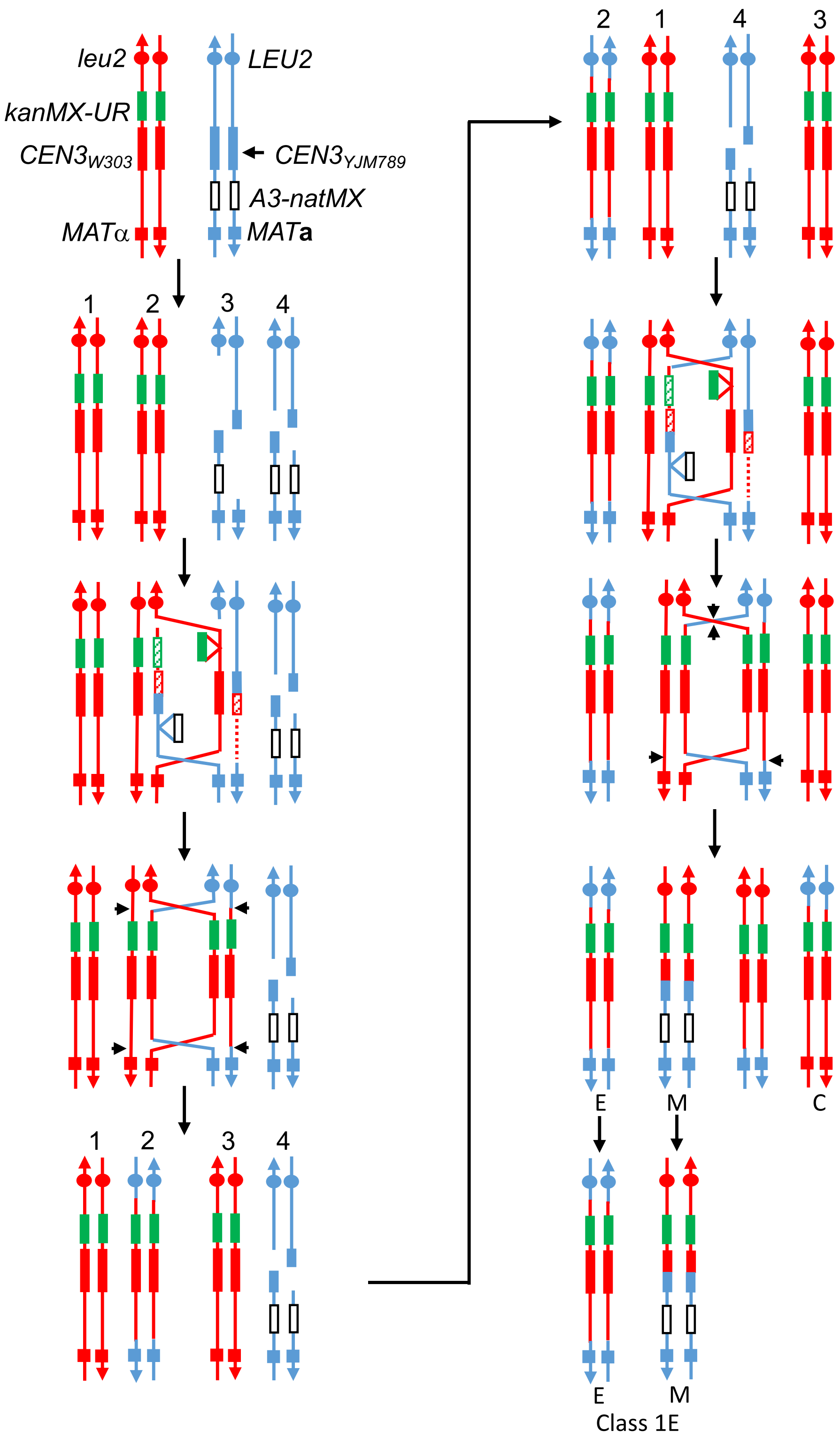

Fig. S6. Class 1E. In Class 1E, as in Class 1D, one of chromatids has a gene conversion unassociated with a crossover ("E" chromatid), and the second chromatid has an associated crossover ("M" chromatid). This class can be explained as a consequence of repair of two broken sister chromatids formed by replication of a YJM789 chromosome with a G1-associated DSB.

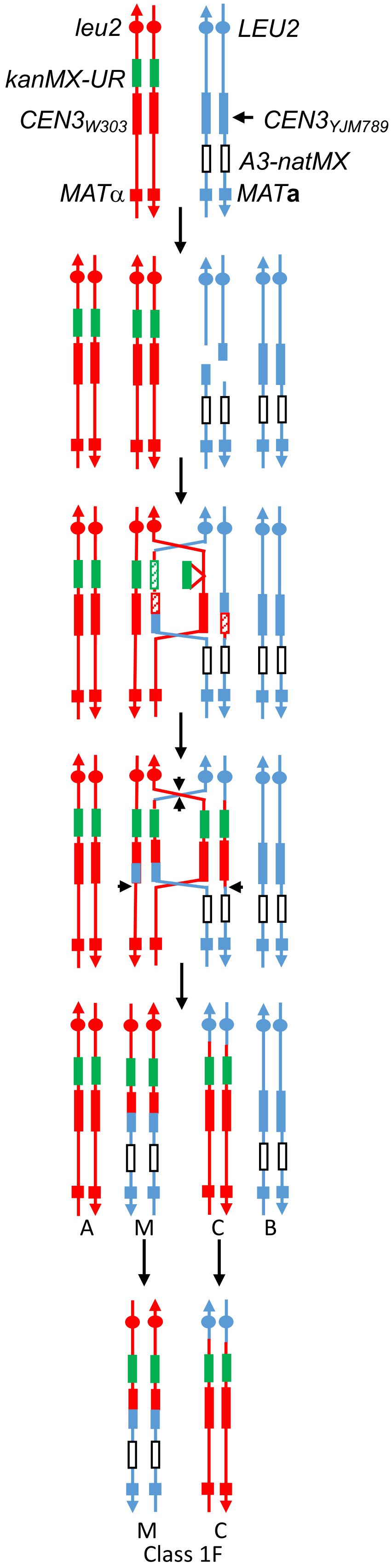

Fig. S7. Class 1F. This class is similar to Class 1A, except in this class, the heteroduplex intermediate is resolved as a crossover.

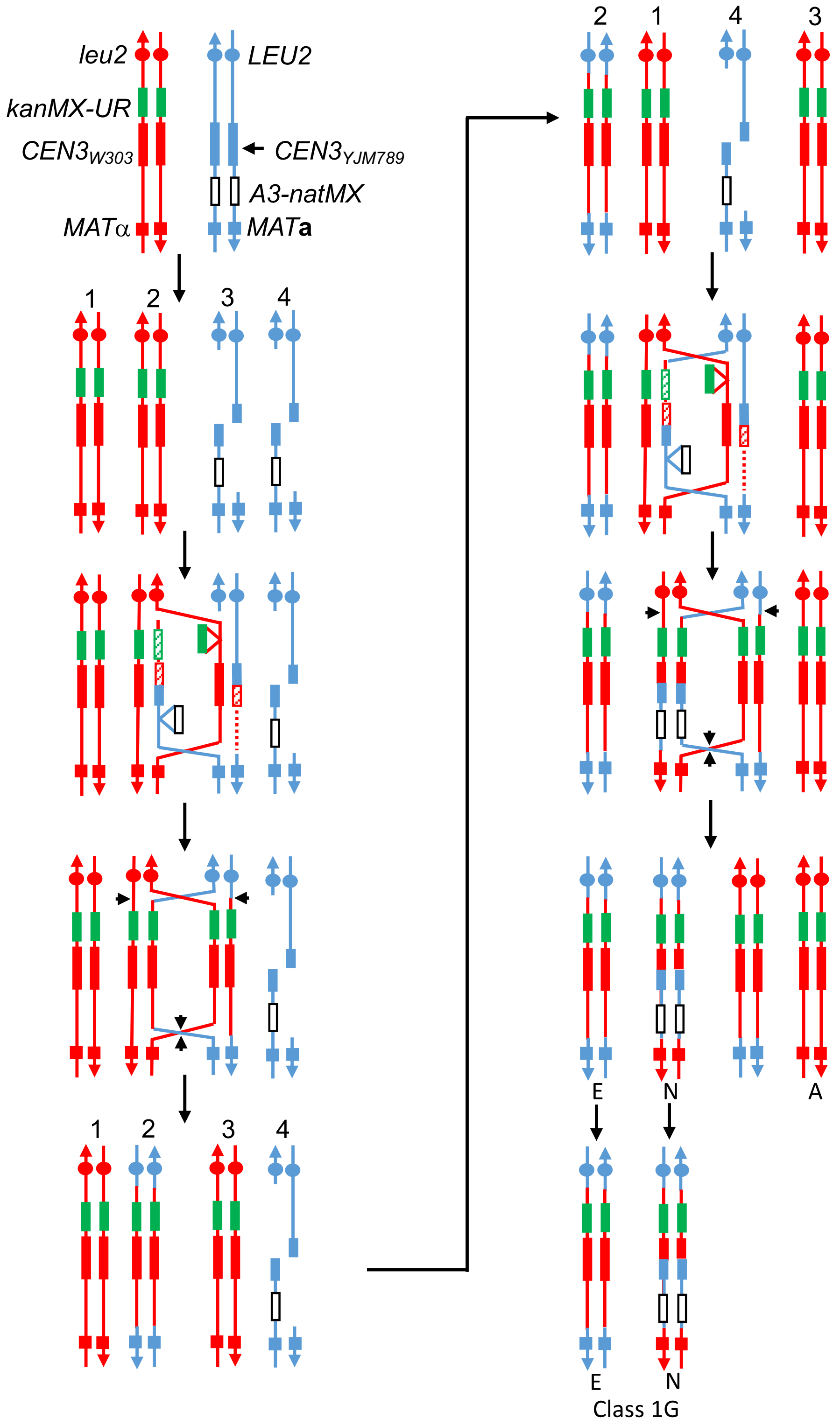

Fig. S8. Class 1G. In this class, the event is initiated by a DSB on the YJM789-derived homolog in G1. One of the resulting DSBs is repaired to produce a single crossover, and the other is repaired to produce a double crossover.

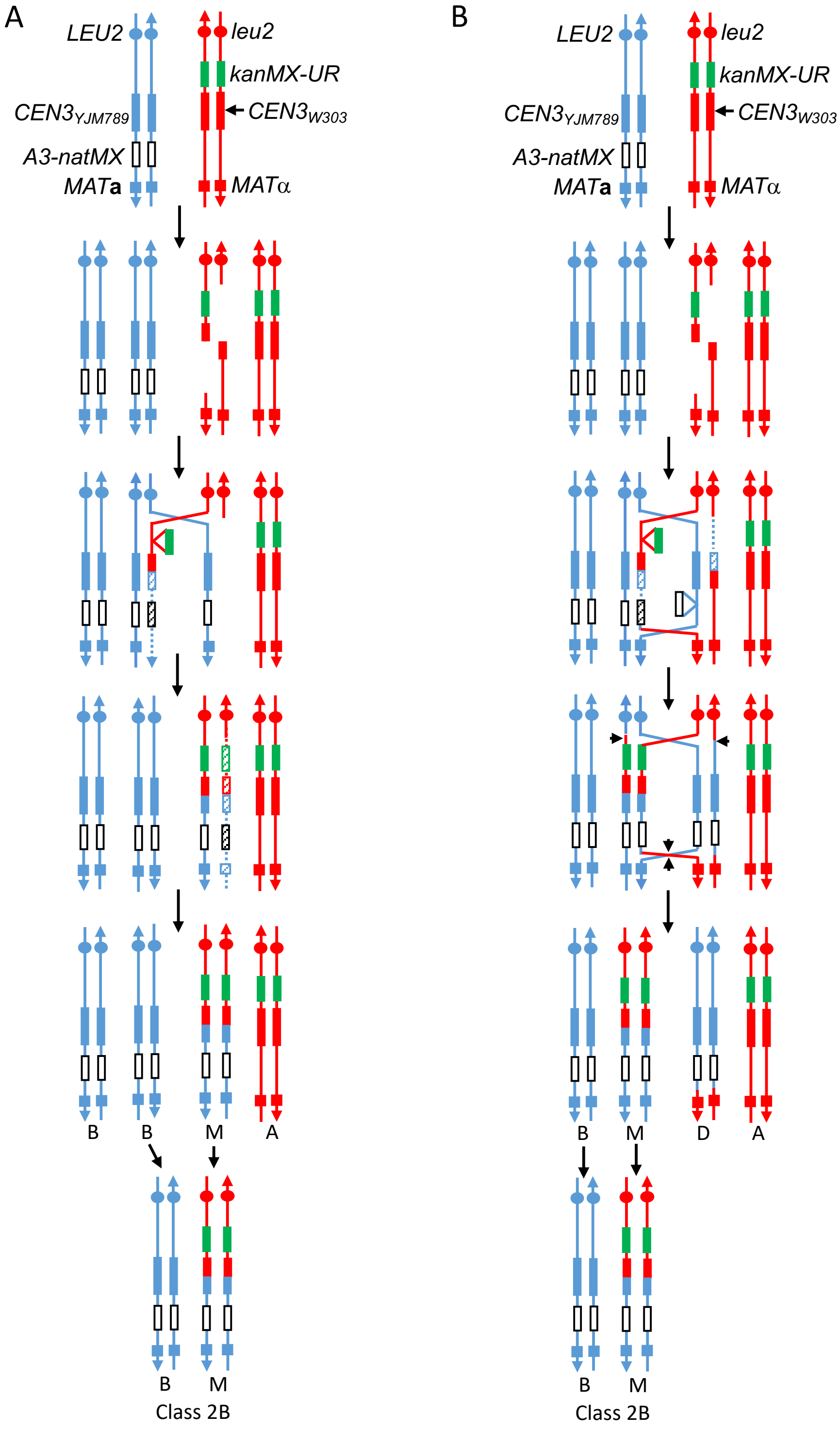

Fig. S9. Class 2B. This sub-class is similar to Class 1B (Fig. 5B,C). As 1B, 2B might be generated either *via* BIR (A) or DSBR (B). Note that in DSBR scheme, RCU recombinant chromosome (M) disjoins into the same cell as its unrecombined sister chromatid (B). RCU recombinant chromosome (M) disjoins into the same cell as its unrecombined sister chromatid (B).

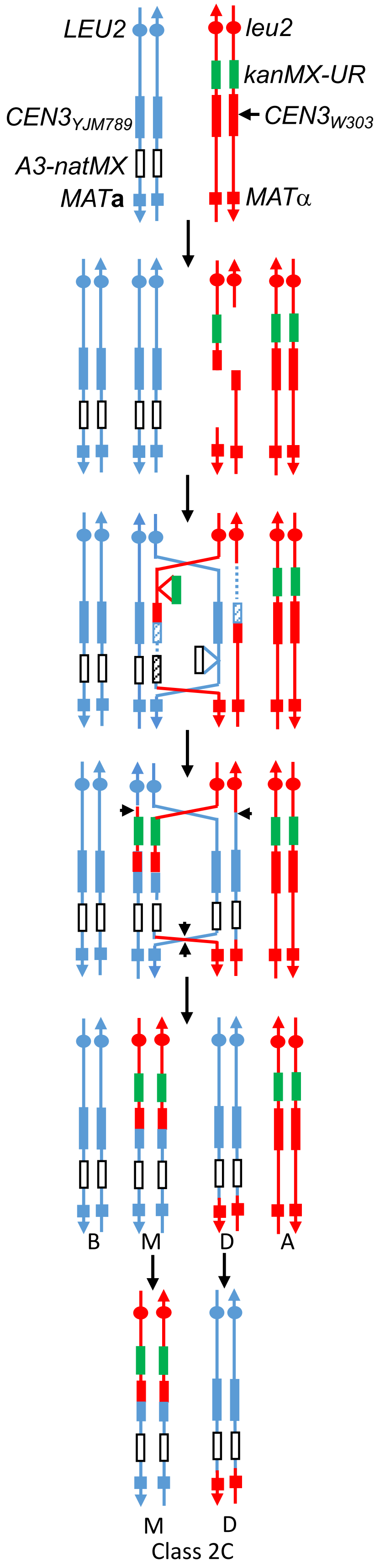

Fig. S10. Class 2C. This sub-class has the pattern of markers expected for repair of a DSB on the W303 homolog by the DSBR pathway; the repair is associated with a CO and the recombinant chromosomes co-segregate. The recombinant events are identical to those shown in Fig. 5B, however, the segregation pattern is different (chromosomes M and D instead of M and A).

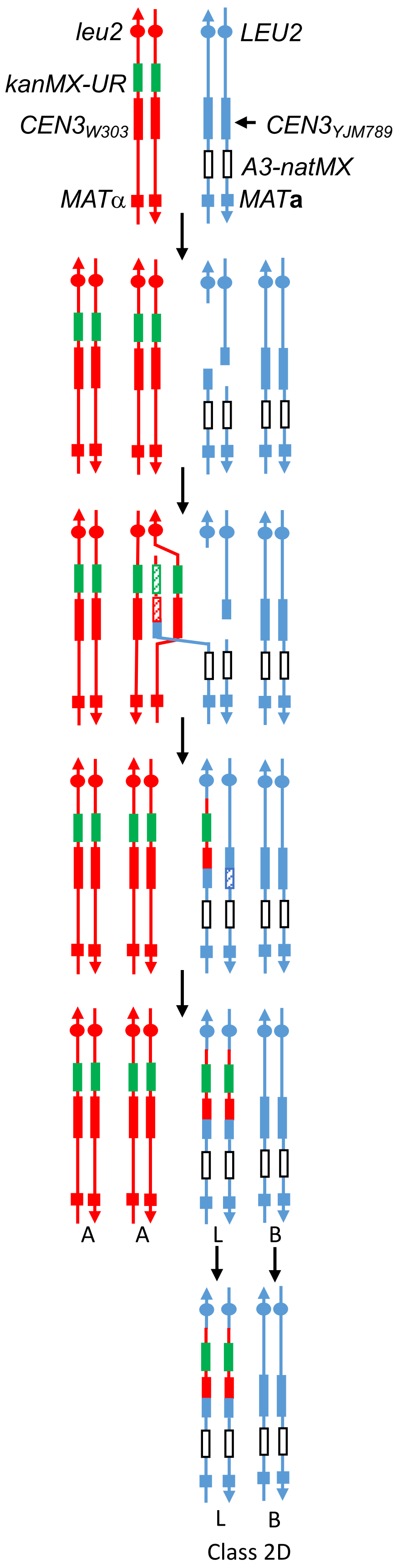

Fig. S11. Class 2D. This sub-class is consistent with a DSB in the centromere of the YJM789 chromosome, followed by a conversion using the SDSA pathway. The recombination event is identical to that shown in Fig. S3 to explain Class 1A, except for the pattern of chromosome segregation. The recombined chromosome co-segregated with the sister unrecombined YJM789 chromosome.

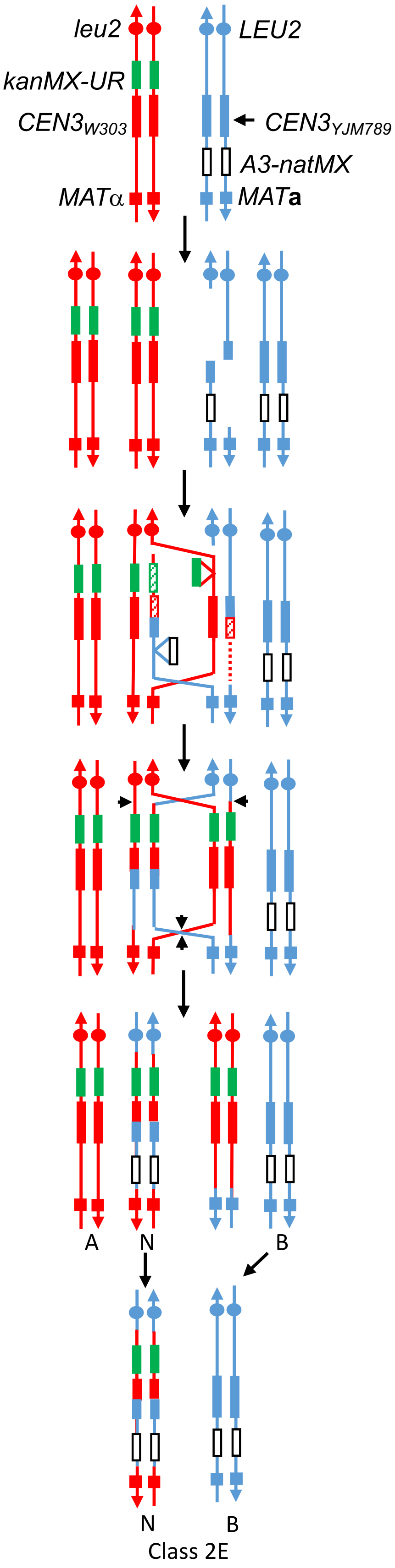

Fig. S12. Class 2E. In this sub-class, a DSB in the YJM789 chromosome is repaired by the DSBR pathway and is associated with a crossover. The recombinant chromosome co-segregates with an unrecombined YJM789 chromatid.

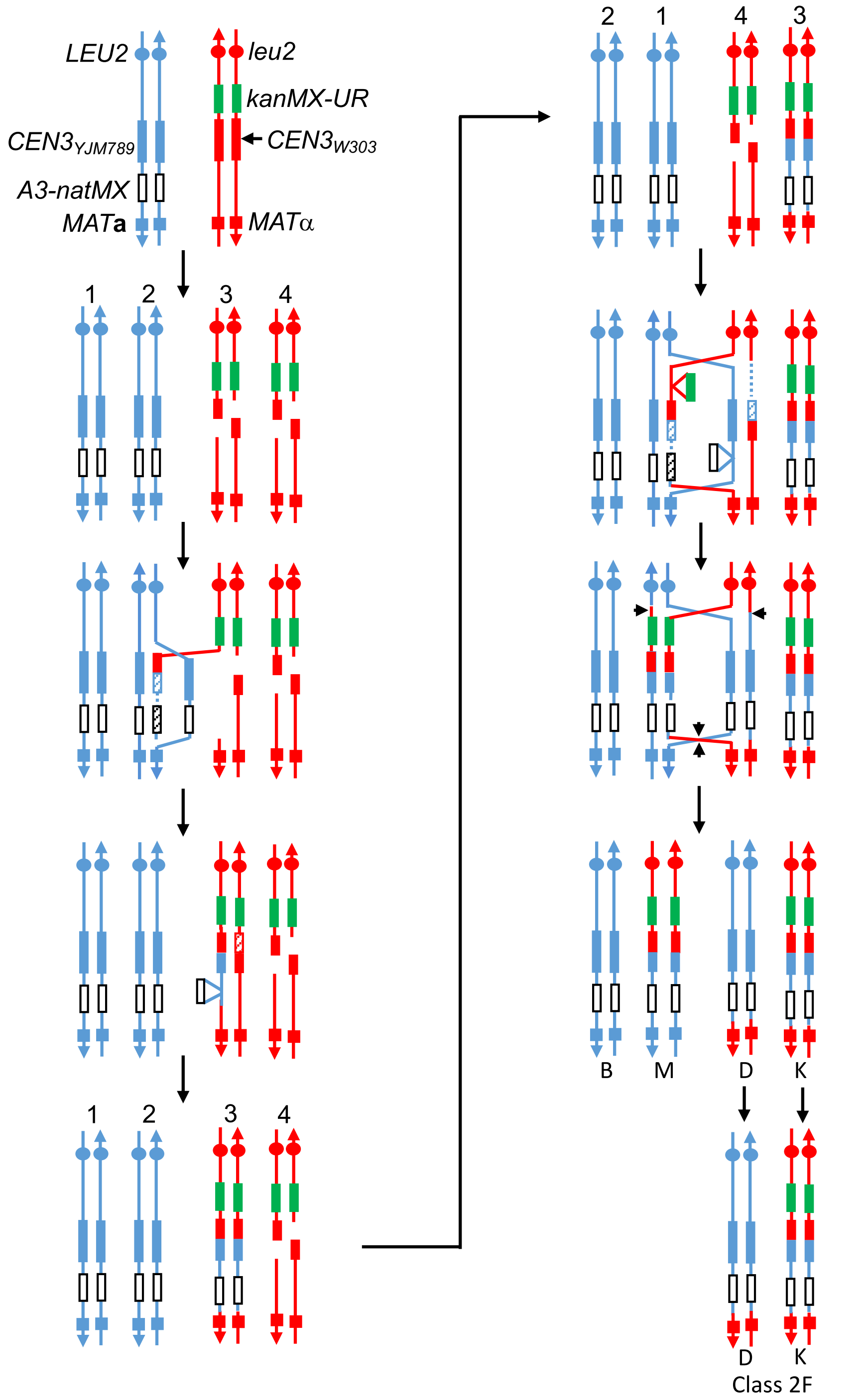

Fig. S13. Class 2F. In this sub-class, there is one chromosome with a crossover without a recombinant centromere (D chromosome) and a second chromosome with a conversion event without a crossover (K chromosome). The event could be a consequence of the repair of a DSB in the W303 chromosome in G1, resulting in two sister chromatids broken at the same position. One is repaired using the SDSA pathway (producing the K chromosome) and the second is repaired in the DSBR pathway (producing the D chromosome).

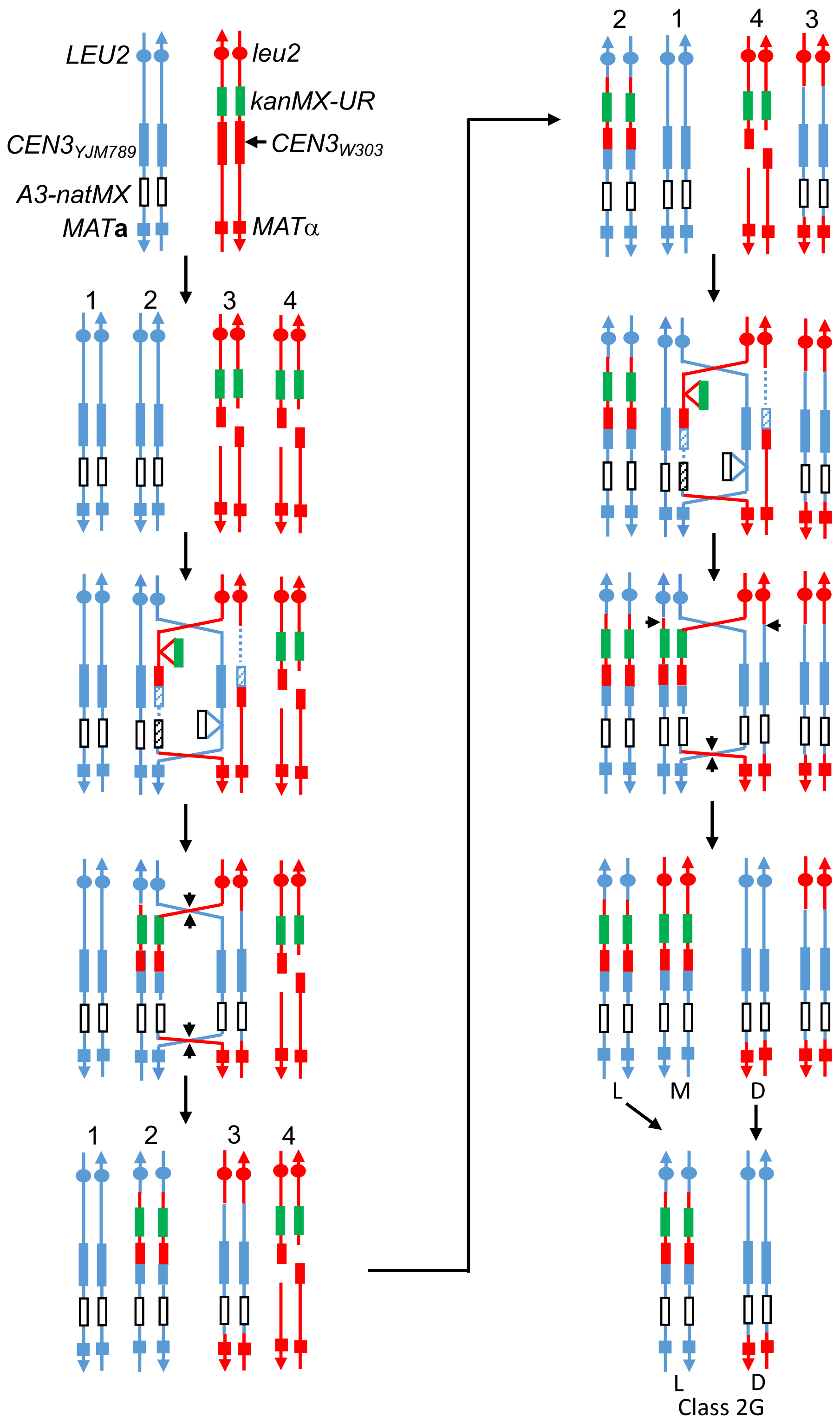

Fig. S14. Class 2G. This pattern is likely a consequence of a G1 DSB in the W303 chromosome, resulting in two broken chromatids that are repaired in the DSBR pathway. One of the chromosomes (L) is not associated with a crossover, and the other (D) is.

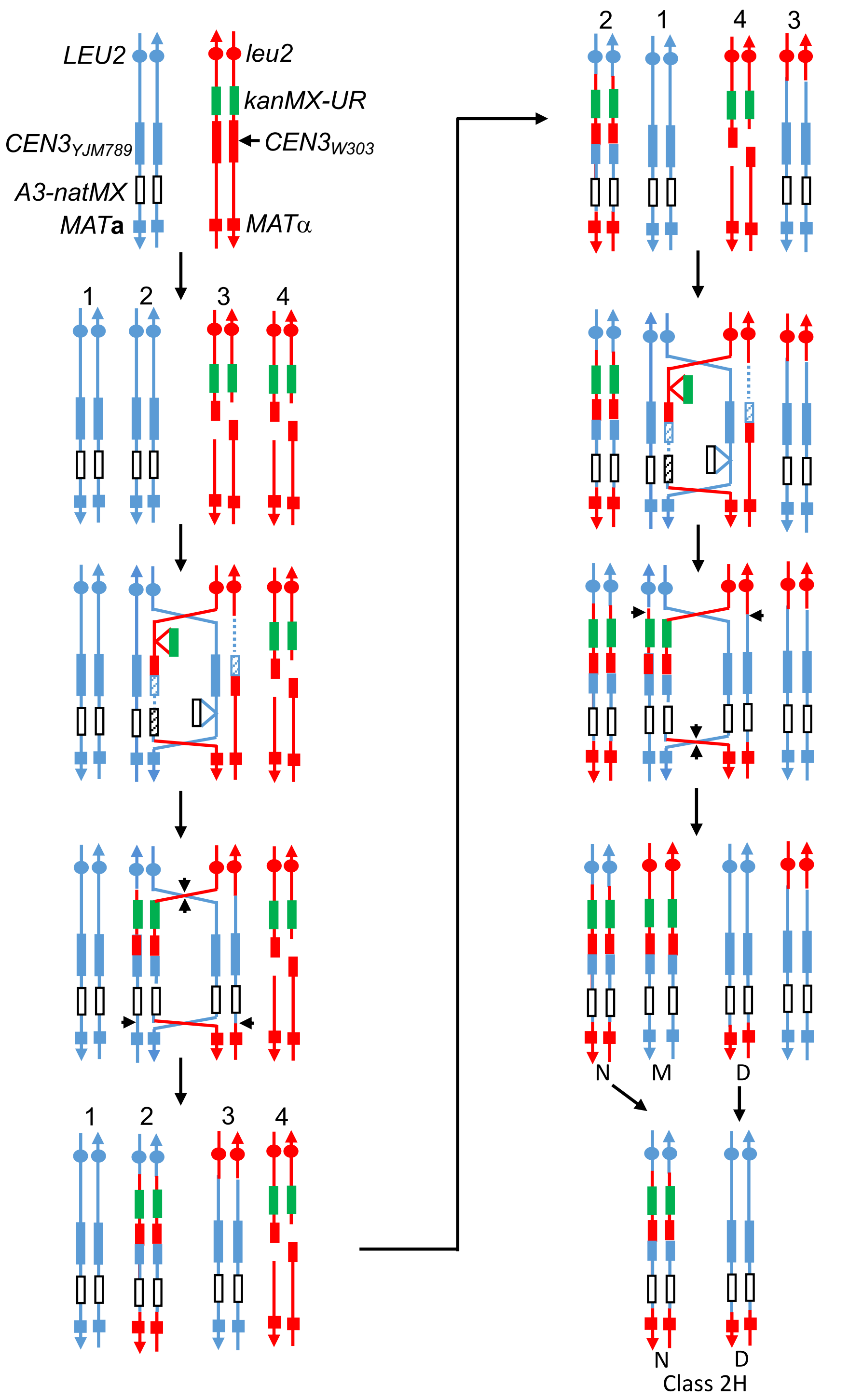

Fig. S15. Class 2H. In this sub-class, both homologs have flanking markers in the recombined configurations, but the two chromosomes do not contain reciprocal crossover products. As with several other of the sub-classes, the pattern of markers observed in Class 2H is consistent with repair of two broken sister chromatids that are the products of a G1-associated break. Both repair events involve the DSBR pathway.

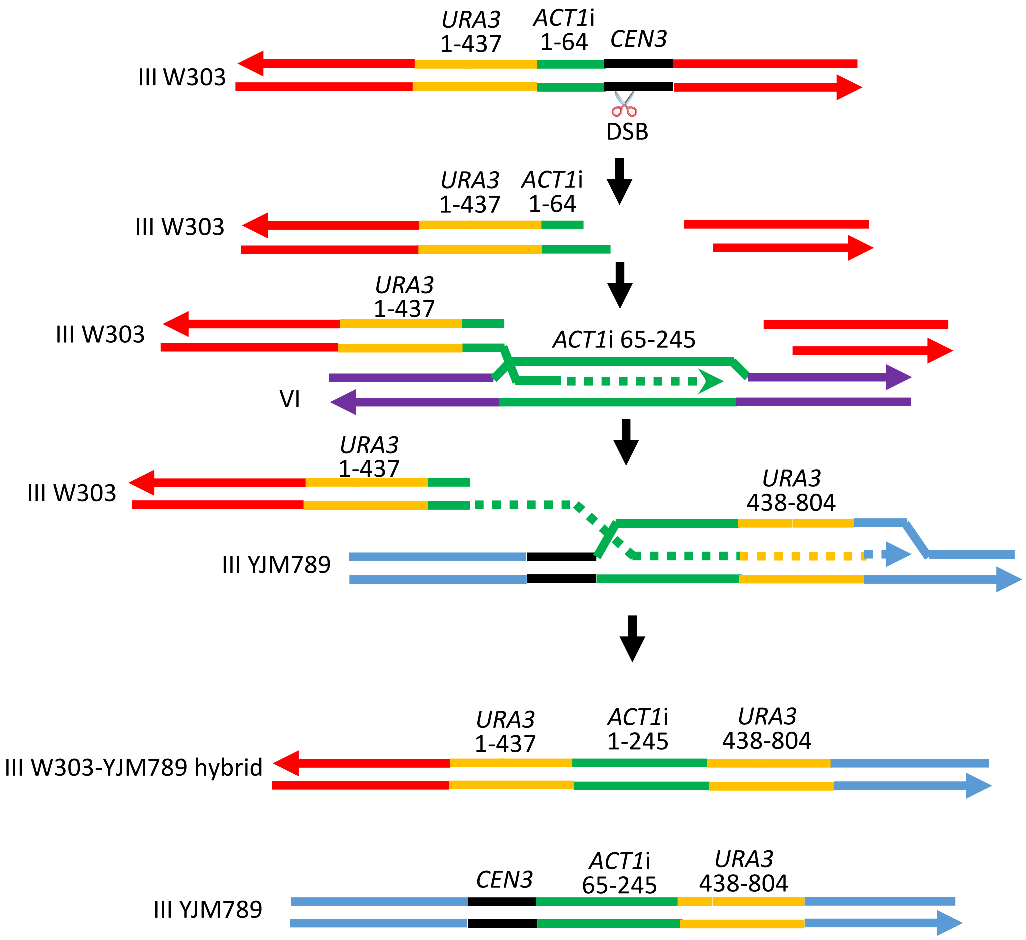

Fig. S16. *URA3* recombinant with deleted *CEN*. In this figure, we show the generation of a recombinant *URA3* gene that is not associated with a recombinant centromere. A DSB within the centromere is processed to produce a chromosome fragment with the left arm of chromosome III and homology to the 5' portion of the *ACT1* intron at its end. This end invades the *ACT1* intron in the *ACT1* gene on chromosome VI. Following a small amount of synthesis duplicating the 3' end of the *ACT1* intron, the end disassociates and invades the *ACT1* intron on the YJM789-derived chromosome III homolog. Continued DNA synthesis would produce an acentric chromosome with the recombinant *URA3* gene.

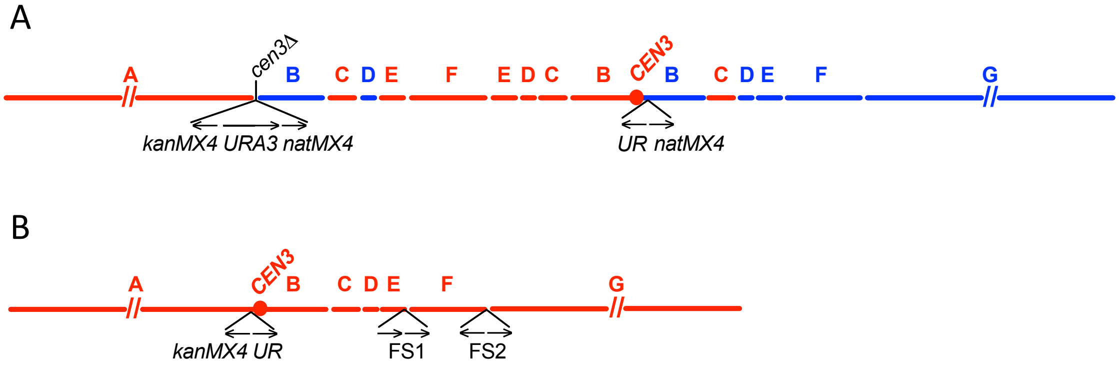

Fig. S17. Maps of the recombinant chromosome III of isolate 15 and the parental chromosome derived from W303. W303- and YJM789-derived sequences are in red and in blue, respectively. Isolate 15 contained a recombinant chromosome and the parental W303 homolog, but not the parental YJM789 homolog. The approximate coordinates for transitions between different segments are: 115 kb (A/B), 132 kb (B/C), 140 kb (C/D), 144 kb (D/E), 150 kb (E/F), and 170 kb (F/G).

A. Recombinant chromosome in isolate 15. Whole-genome sequencing of Isolate 15 indicated that several internal segments of the chromosome III were duplicated or triplicated (Fig. S18). In addition, a SNP-microarray analysis of the recombinant chromosome III purified from a CHEF gel confirmed the presence of a large internal duplication. We propose the following mechanism to produce the recombinant chromosome. First, a DSB occurred at or near *CEN3* on the W303-derived homolog, and DNA repair was initiated at the *ACT1* intron on chromosome VI (similar to the mechanism shown in Fig. S16). Following limited DNA synthesis, the broken end disengaged and copied segment B on the YJM789 homolog. Second, there were several cycles of short BIR events between the two homologs, producing a mosaic chromosome with both W303- and YJM789-derived sequences. Third, when the BIR event reached a pair of directly-oriented Ty elements between segments E and F (FS1, Lemoine *et al.*, 2005), the invading strand disengaged from the template and re-invaded a Ty element (one of an inverted pair of Ty elements located between segments F and G, FS2) oriented in the opposite direction from the Ty1 pair located between E and F; the location and orientation of Ty1 elements on chromosome III were established previously (Lemoine *et al.*, 2005). The invaded strand then copied segments F, E, D, C, and B, producing a large inversion/duplication. After the BIR replication fork reached the A/B junction, the end containing the *TEF* promoter of the *kanMX* cassette dissociated from the template and invaded the

TEF promoter of the *natMX* cassette. Synthesis continued, duplicating the “blue” B and “red” C segments. Lastly, the invading strand disassociated from the template and copied the segments D-G on the YJM789-derived homolog.

The proposed mechanism is based on the DNA sequence coverage shown in Fig. S18, microarray analysis of the purified recombinant chromosome, and sequence analysis of several unique PCR products. One of the PCR products demonstrating the inversion utilized a primer specific for the directly-oriented Ty1 pair (FS1) and a second primer located downstream of the inverted pair of Ty1 elements (FS2); sequencing confirmed the inversion. The inversion with the B/B junction was confirmed with the primers URA3-intR and CEN3-verR (Table S2).

B. Parental W303 chromosome III homolog.

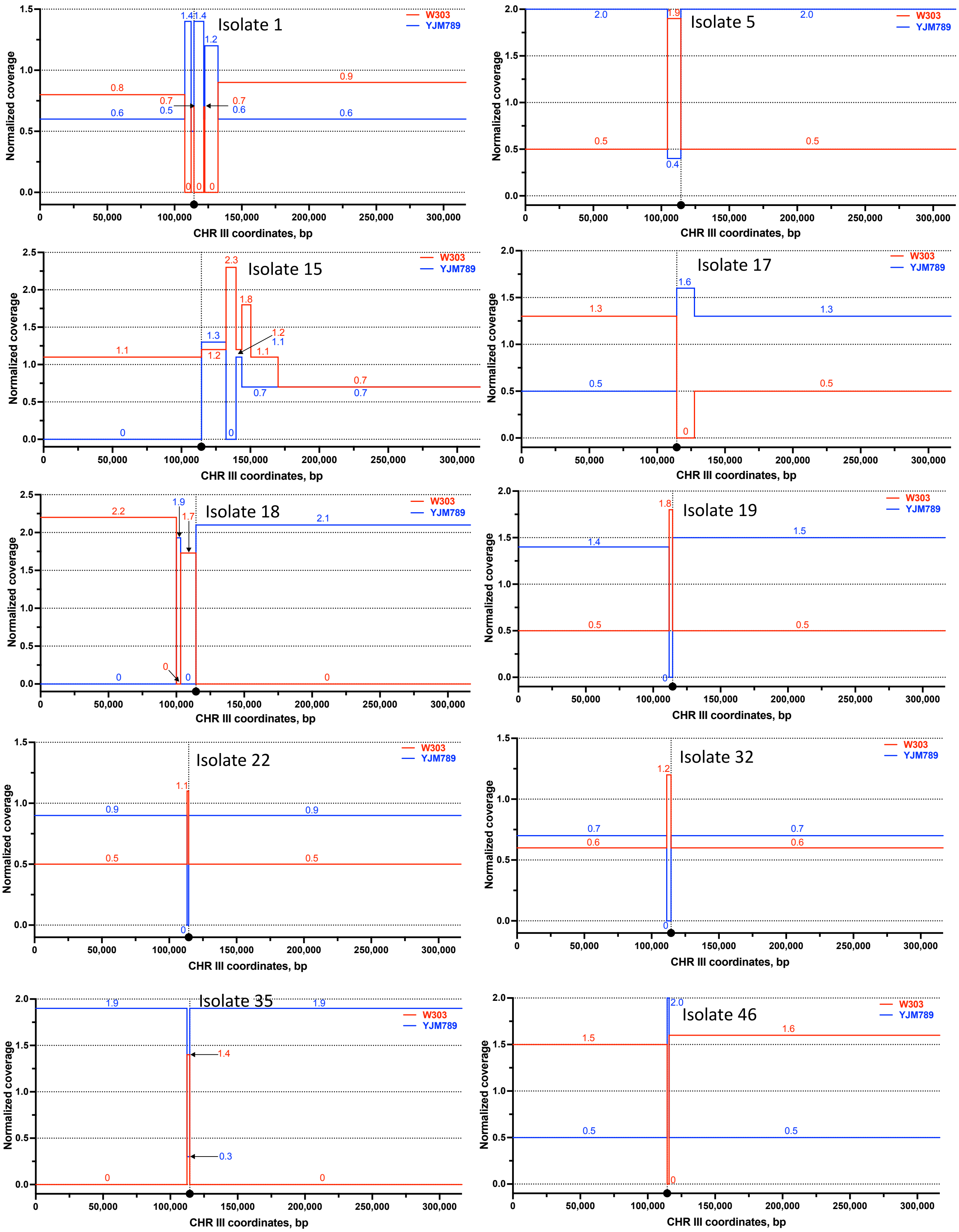

Fig. S18 (continued on next page)

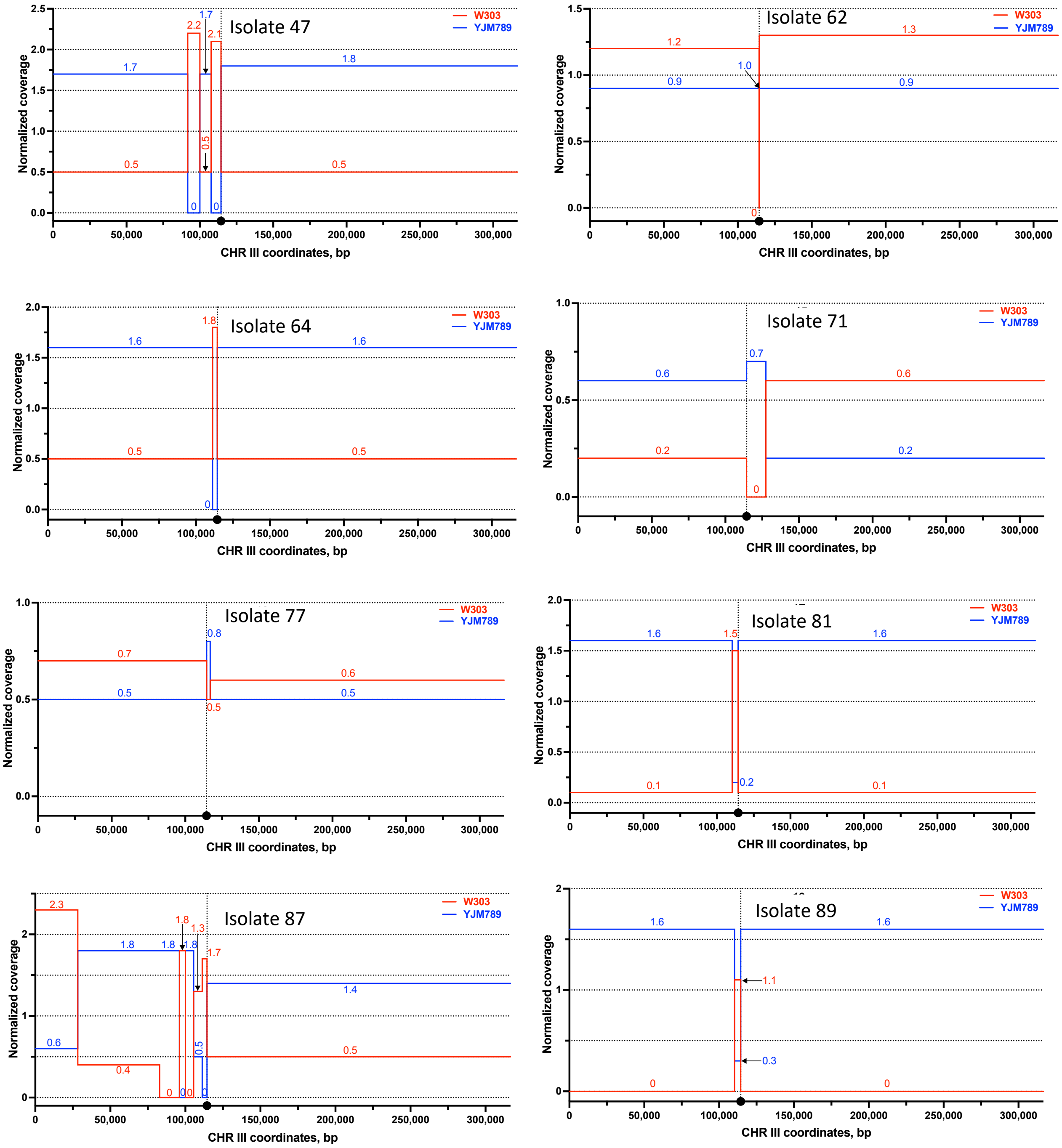

Fig. S18. Depictions of sequence coverage on chromosome III of SGK169. DNA samples were isolated from strains grown in medium lacking uracil to force retention of the chromosome with the recombinant *URA3* gene; since the recombinant *URA3* gene is weakly expressed, this growth condition likely selects for cells with more than one copy of the *URA3*-containing chromosome. In this figure, we show the normalized sequence coverage of SNPs derived from W303 and YJM789 on chromosome III in Ura^{+} strains derived from SGK169. Coverage was normalized to an average whole-genome coverage for all sequences as described in text; one copy per diploid has a normalized value of 0.5. These values are affected by both LOH events and subsequent loss or gain of chromosomes. These depictions are based on the data shown in Table S5. The black circle on the abscissa axis represents *CEN3*.

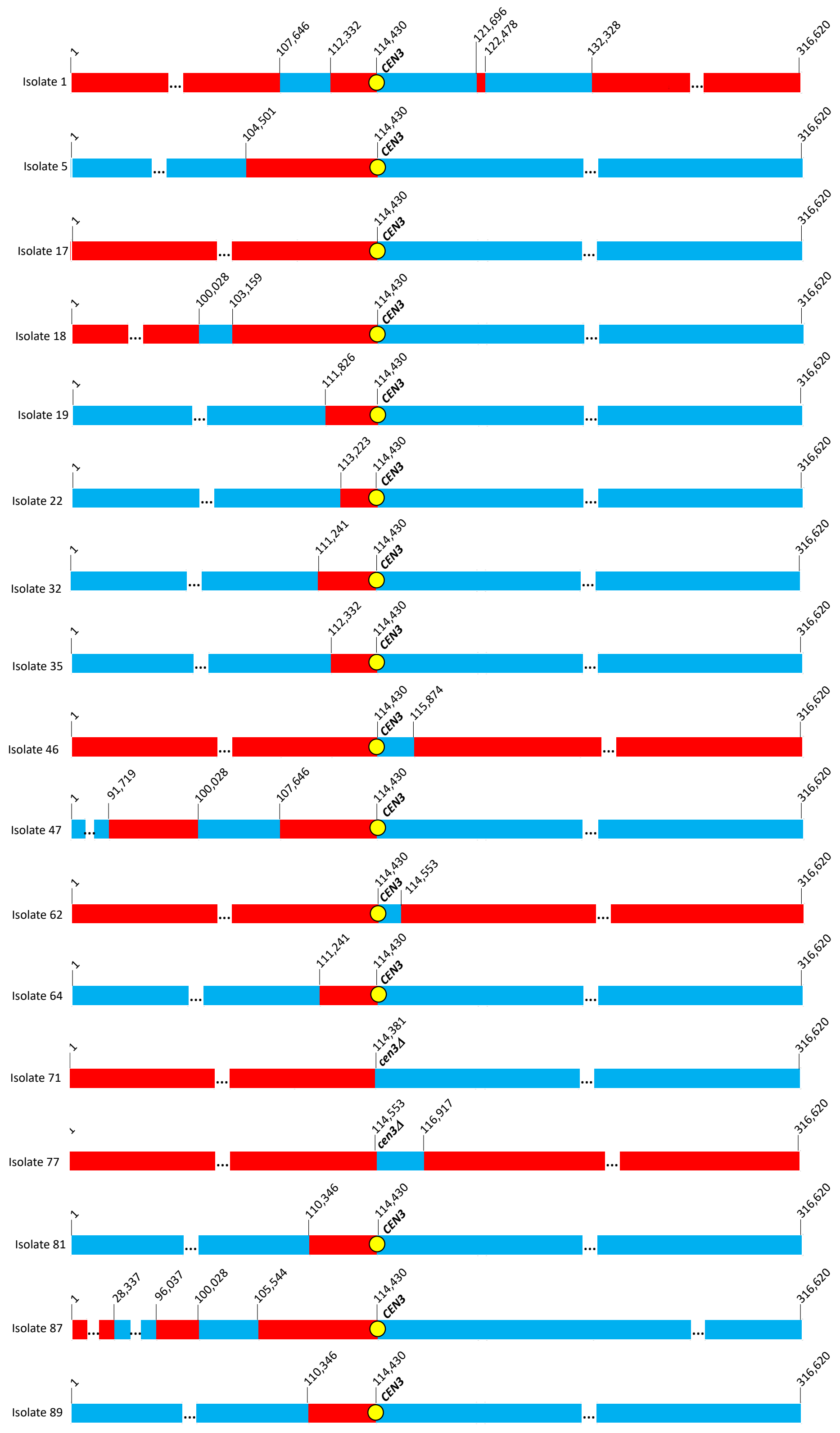

Fig. S19. Summary of gene conversion tracts in SGK169 Ura^+ isolates. Based on our whole-genome sequencing analysis, we show the contribution of W303- (red) and YJM789-derived (blue) sequences to gene conversion tracts on the chromosome III homolog that contains the recombinant centromere and *URA3* gene. The numbers at the breakpoints are SGD coordinates. Isolates 4 (meiotic product) and 15 (described in Fig. S17) are omitted from the figure.

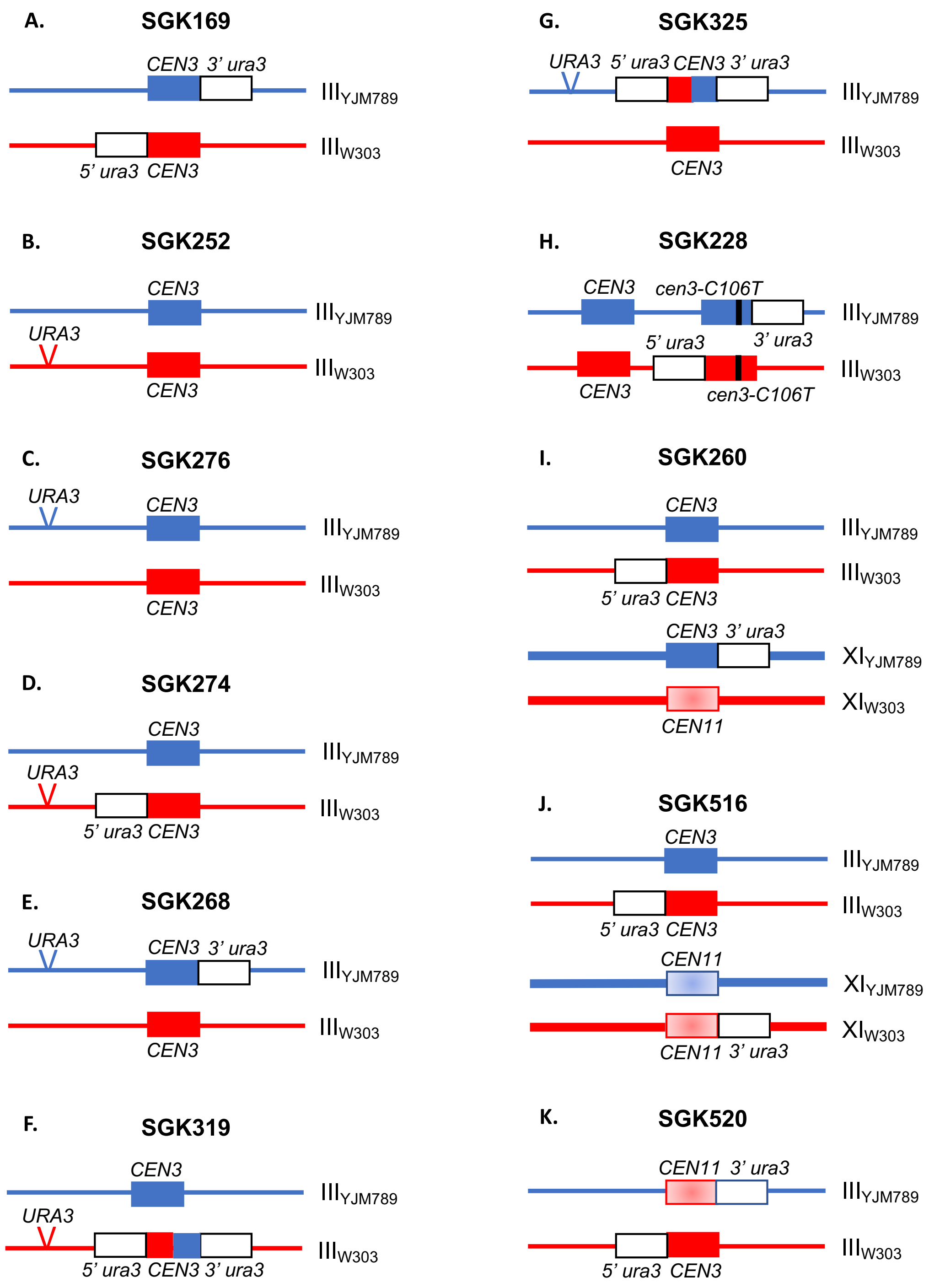

Fig. S20. Schematic diagrams of strains used to monitor chromosome loss rates, recombination of non-functional centromeres, or recombination between centromeres on non-homologs. As in other figures, red and blue lines indicate the W303- and YJM789-derived homologs, respectively. *ACT1* introns and drug

resistance genes flanking the centromeres are not shown. The ectopic positions of *URA3* genes on chromosome III are indicated by carets. The ectopic *URA3* gene (SGD coordinates 115926-117030 of chromosome V) was inserted onto the left arm of III between SGD coordinates 57000 and 57001. The location of *CEN3* in wild-type strains is between chromosome III SGD coordinates 114385 and 114501. Chromosome III and XI centromeres are shown as solid red and blue colors and shaded red and blue colors, respectively. Other details of the structures of these chromosomes are given in the text and Supplemental Information.

Supplemental References

Aksenova,A.Y., Greenwell,P.W., Dominska,M., Shishkin,A.A., Kim,J.C., Petes,T.D. and Mirkin,S.M. (2013) Genome rearrangements caused by interstitial telomeric sequences in yeast. *Proc. Natl. Acad. Sci. U. S. A.*, **110**, 19866-19871.

Altman,D.G. (1991) In: *Practical Statistics for Medical Research*. Chapman and Hall/CRC Publishers, Boca Raton, FL.

Drake,J.W. (1991) A constant rate of spontaneous mutations in DNA-based microbes. *Proc. Natl. Acad. Sci. U. S. A.*, **88**, 7160-7164.

Lee,P.S. and Petes,T.D. (2010) Mitotic gene conversion events initiated in G1-synchronized yeast cells by gamma rays are similar to spontaneous conversion events. *Proc. Natl. Acad. Sci. U. S. A.*, **107**, 7383-7388.

Lemoine,F., Degtyareva,N.P., Lobachev,K. and Petes,T.D. (2005) Chromosomal translocations in yeast induced by low levels of DNA polymerase alpha: a model for chromosome fragile sites. *Cell*, **120**, 587-598.

McCulley,J.L. and Petes,T.D. (2010) Chromosome rearrangements and aneuploidy in yeast strains lacking both Tel1p and Mec1p reflect deficiencies in two different mechanisms. *Proc. Natl. Acad. Sci. U. S. A.*, **107**, 11465-11470.

Mortimer,R.K. and Johnston,J.R. (1986) Genealogy of principal strains of the yeast genetic stock center. *Genetics*, **113**, 35-43.

Rose,M.D., Winston,F. and Hieter,P. (1990) In: *Methods in Yeast Genetics: A Laboratory Course Manual*. Cold Spring Harbor Laboratory Press, NY.

St. Charles,J., Hazkani-Covo,E., Yin,Y., Andersen,S.L., Dietrich,F.S., Greenwell,P.W., Malc,E., Mieczkowski,P. and Petes,T.D. (2012) High-resolution genome-wide analysis of irradiated (UV and gamma-rays) cells reveals a high frequency of genomic loss of heterozygosity (LOH) events. *Genetics*, **190**, 1367-1284.

St. Charles,J. and Petes,T.D. (2013) High-resolution mapping of spontaneous mitotic recombination hotspots on the 1.1 Mb arm of yeast chromosome IV. *PLoS Genet*., **9**, e1003434.

Thomas,B.J. and Rothstein,R. (1989) Elevated recombination rates in transcriptionally active DNA. Cell, 56, 619-630.

Wei,W., McCusker,J.H., Hyman,R.W., Jones,T., Ning,Y., Cao,Z. *et al.* (2007) Genome sequencing and comparative analysis of *Saccharomyces cerevisiae* strain YJM789. *Proc. Natl. Acad. Sci. U. S. A*., **104**, 12825-12830.

Zheng,D.Q., Zhang,K., Wu,X.C., Mieczkowski,P.A. and Petes,T.D. (2016) Global analysis of genomic instability caused by DNA replication stress in Saccharomyces cerevisiae. *Proc. Natl. Acad. Sci. U. S. A*., **113**, e2119588119.

Zheng,Q. (2017) rSalvador: an R package for the fluctuation experiment. *G3 (Bethesda)*, **7**, 3849-3856.
